# The Roles of NOTCH3 p.R544C and Thrombophilia Genes in Vietnamese Patients With Ischemic Stroke: Study Involving a Hierarchical Cluster Analysis

**DOI:** 10.2196/56884

**Published:** 2024-05-07

**Authors:** Huong Thi Thu Bui, Quỳnh Nguyễn Thị Phương, Ho Cam Tu, Sinh Nguyen Phuong, Thuy Thi Pham, Thu Vu, Huyen Nguyen Thi Thu, Lam Khanh Ho, Dung Nguyen Tien

**Affiliations:** 1 Department of Biochemistry Thai Nguyen University of Medicine and Pharmacy Thai Nguyen Vietnam; 2 Department of Immunology Molecular Genetic Thainguyen National Hospital Thai Nguyen Vietnam; 3 Department of Clinical Pharmacy Thai Nguyen University of Medicine and Pharmacy Thai Nguyen Vietnam; 4 Center of Gene and Protein Research Hanoi Medical University Hanoi Vietnam; 5 Institute of Virology School of Medicine Technical University of Munich Munich Germany; 6 Department of Rehabilitation Thai Nguyen University of Medicine and Pharmacy Thai Nguyen Vietnam; 7 Department of Internal Medicine Thai Nguyen University of Medicine and Pharmacy Thai Nguyen Vietnam; 8 Department of Telecomunication Hung Yen University of Technology and Education Hung Yen Vietnam

**Keywords:** Glasgow Coma Scale, ischemic stroke, hierarchical cluster analysis, clustering, machine learning, MTHFR, NOTCH3, modified Rankin scale, National Institutes of Health Stroke Scale, prothrombin, thrombophilia, mutations, genetics, genomics, ischemia, risk, risk analysis

## Abstract

**Background:**

The etiology of ischemic stroke is multifactorial. Several gene mutations have been identified as leading causes of cerebral autosomal dominant arteriopathy with subcortical infarcts and leukoencephalopathy (CADASIL), a hereditary disease that causes stroke and other neurological symptoms.

**Objective:**

We aimed to identify the variants of *NOTCH3* and thrombophilia genes, and their complex interactions with other factors.

**Methods:**

We conducted a hierarchical cluster analysis (HCA) on the data of 100 patients diagnosed with ischemic stroke. The variants of *NOTCH3* and thrombophilia genes were identified by polymerase chain reaction with confronting 2-pair primers and real-time polymerase chain reaction. The overall preclinical characteristics, cumulative cutpoint values, and factors associated with these somatic mutations were analyzed in unidimensional and multidimensional scaling models.

**Results:**

We identified the following optimal cutpoints: creatinine, 83.67 (SD 9.19) µmol/L; age, 54 (SD 5) years; prothrombin (PT) time, 13.25 (SD 0.17) seconds; and international normalized ratio (INR), 1.02 (SD 0.03). Using the Nagelkerke method, cutpoint 50% values of the Glasgow Coma Scale score; modified Rankin scale score; and National Institutes of Health Stroke Scale scores at admission, after 24 hours, and at discharge were 12.77, 2.86 (SD 1.21), 9.83 (SD 2.85), 7.29 (SD 2.04), and 6.85 (SD 2.90), respectively.

**Conclusions:**

The variants of *MTHFR* (C677T and A1298C) and *NOTCH3* p.R544C may influence the stroke severity under specific conditions of PT, creatinine, INR, and BMI, with risk ratios of 4.8 (95% CI 1.53-15.04) and 3.13 (95% CI 1.60-6.11), respectively (*P_fisher_*<.05). It is interesting that although there are many genes linked to increased atrial fibrillation risk, not all of them are associated with ischemic stroke risk. With the detection of stroke risk loci, more information can be gained on their impacts and interconnections, especially in young patients.

## Introduction

Stroke is a medical condition involving the disruption of blood flow, which leads to brain cell death. There are several risk factors for stroke, including high blood pressure, smoking, diabetes, and increased cholesterol levels. In 2019, the Global Burden of Disease analysis assessed that there were 12.2 million incident cases of stroke and 101 million prevalent cases of stroke, with 6.55 million deaths [1]. The burden of stroke is the highest in low- and middle-income countries, where risk factors, such as high blood pressure, smoking, and insufficient diet, are more prevalent [1].

The overall population of Vietnam was estimated to be 98.32 million in 2021, with young people accounting for the majority of the population and people aged older than 65 years accounting for only 7.7% of the population. This phenomenon is the leading cause of death and disability in Vietnam. The incidence and prevalence of stroke have been reported to be 161 and 415 per 100,000 people, respectively [2]. Stroke is broadly classified into the following 3 types: ischemic stroke, hemorrhagic stroke, and subarachnoid hemorrhage. Ischemic stroke results from the blockage of blood vessels, which limits blood flow to the brain. Approximately 60%-80% of all stroke cases are ischemic. This study focused on acute ischemic stroke and its genetic features. The unmodifiable risk factors include age, race, sex, ethnicity, history of migraine headaches, and fibromuscular dysplasia. Moreover, the hereditary factors include a family history of stroke or transient ischemic attacks. Furthermore, the modifiable risk factors include hypertension, diabetes mellitus, cardiac disease, high cholesterol levels, previous stroke, carotid stenosis, hyperhomocysteinemia, and lifestyle issues. The majority of ischemic strokes seen in patients with cardiovascular disease are embolic [3].

The etiology of ischemic stroke is multifactorial. Although receiving a minor focus, genetic factors considerably contribute to the occurrence of ischemic stroke, particularly in cases of early-onset stroke. Several stroke classification systems have been proposed based on genetic information corresponding to various stroke phenotypes. Twin and family history studies and the candidate gene approach are standard methods to discover genetic causes of stroke. However, both methods have their limitations. Some monogenic disorders (7% of stroke etiology) may generate well-known clinical indications that include stroke. Polygenic disorders are more frequent, causing 38% of ischemic stroke cases, and their designation is a rapidly evolving field of current stroke genetics. Recent advances in human genetics provide opportunities for personalized stroke prevention and unknown cure options. Some authors have boosted the application of stroke gene panels for stroke hazard evaluation and stroke research. Ilinca et al [4] have created stroke gene panels for research and clinical practice. The clinical panel includes 61 genes related to stroke directly and 27 additional genes related to disorders causing stroke, and it might be relevant to consider their evaluation in clinical practice. The authors encourage the use of their panels for stroke risk evaluation and further stroke research [4]. Another benefit of detecting stroke risk genes is that they could be potential targets for gene therapy in the future. Histone deacetylase (HDAC) inhibitors have been postulated as a treatment for stroke [5]. A study in knock-out mice suggested a new strategy for acute stroke treatment by suppressing HDAC2 in the peri-infarct zone [6]. The authors claim that application of HDAC inhibitors from 5 to 7 days after stroke enhances cell survival and neuroplasticity as well as reduces inflammation, which could potentially provide a wider therapeutic window for stroke recovery [6]. Systemic administration of an agonist *NOTCH3* antibody was studied in transgenic mice and showed protective effects against impaired cerebral blood flow [7]. Transcriptome-wide colocalization analyses showed an association of white matter hyperintensity-volume with the expression of 39 genes, of which 4 encode known drug targets [8]. Moreover, unknown biomarkers for stroke hereditary causes and novel markers for gene therapy are on the horizon [9].

Machine learning–based models performed better in predicting poststroke outcomes than regression models using the items of conventional stroke prognostic scores, although they required additional variables, such as laboratory data, to attain improved performance, and further studies are warranted to validate the usefulness of machine learning in clinical settings [10].

Following our previous hierarchical cluster analysis (HCA) study [11], we assessed the overall preclinical characteristics, cumulative cutpoint values, and factors associated with thrombophilia genes and the *NOTCH3* p.R544C variant in unidimensional and multidimensional analyses involving ischemic stroke patients from Vietnam.

## Methods

### Study Design

We used convenience sampling to include 100 patients with cerebral infarction (ischemic stroke) who were diagnosed as having acute ischemic stroke according to the clinical standards of the World Health Organization and the results of diagnostic imaging (computed tomography [CT], magnetic resonance imaging [MRI], or computed tomography angiography [CTA]) and who had been or are being treated at the Stroke Center, Thai Nguyen Central Hospital. Patients who were residents of the northern mountainous provinces, were ≤60 years old at the time of the first stroke, and were willing to participate in the research were considered for inclusion. Patients with cerebral venous sinus thrombosis, intracranial hemorrhage, and subarachnoid hemorrhage were excluded. We collected information on stroke risk factors from the medical history of patients, including hypertension, diabetes, coronary artery disease, history of stroke, atrial fibrillation, smoking, headache, hyperlipidemia, valve replacement, thyroid dysfunction, history of abortion, vascular disease, blood disorders, chronic alcohol consumption, and use of oral contraceptives. Patients were required to undergo routine biochemical and hematological tests, Doppler ultrasound of the carotid and vertebral arteries, MRI or CTA of the brain, coagulation tests, fibrinogen tests, and homocysteine tests. Based on the findings of a previous study [2], we suppose that in 100 ischemic patients with a confidence level of 95%, the margin of error will be ±7.84% of the population size (stroke in general), with 80% ischemic type. The margin of error formula is as follows:



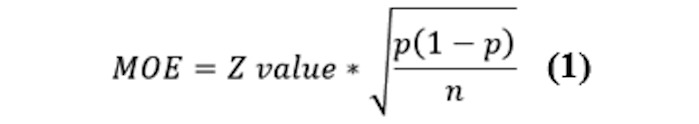



where *Z* value is the critical *Z* value that corresponds to the confidence level, *p* is the sample proportion or percentage, and *n* is the sample size.

A sample size with sufficient statistical power is critical to the success of genetic association studies for detecting causal genes of human complex diseases, especially in the case of ischemic stroke. We selected a 2-tailed test with a type I error of 0.05 as we wanted to assess the average continuous levels (preclinical factors) of patients from different cutpoints. In clinical and biological studies, the effect size *d* following Cohen criteria (the degree of difference between two or more groups) is important. Cohen *d* is the ratio of ∆ and σ (*d*=∆/σ), where σ is the standard deviation and ∆ is an influence index of the risk factors (treatment, genotype, etc) on the population phenotype. In our study, we calculated Cohen *d* according to the supposed sample size of 50-100. With a power of 80% and using a 2-sided *t* test, we estimated that *d* could be from 0.4 (sample size of each group is 99) to 0.7 (sample size of each group is 45). The sample size calculation formula is as follows:



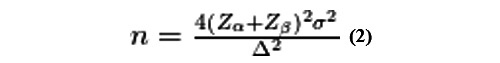



In this formula, the 2-sided confidence level is Z_α/2_, α is the possibility of making a type I error, and β is the possibility of making a type II error. The power of the study is 1-β.

Thus, screening all risk factors may have a medium or higher level of influence on the phenotype (*P*<.05 indicates statistical significance) (Table 1).

**Table 1:** Two-sample t test power calculation results.

Sample size for each group	Cohen *d*^a^
99.08	0.4
63.76	0.5
44.58	0.6
33.02	0.7

^a^The general guidelines for interpreting the effect size are as follows: 0.2-0.49, small effect; 0.5-0.79, moderate effect; 0.8-1.0, large effect; >1.0, very large effect.

### Genetic Testing

Polymorphisms of *NOTCH3* p.R544C, *FV-H1299R*, *MTHFR-C677T*, *MTHFR-A1298C*, *FII-Prothrombin*, *FV-Cambridge*, *PAI1 4G/5G*, and *FXIII Val34Leu* were analyzed using polymerase chain reaction with confronting 2-pair primers (PCR-CTPP) and the thrombophilia genetic assay. The peripheral blood of study participants was collected in EDTA-containing tubes using a standard blood collection procedure. Whole-genome DNA was extracted from 2-3 mL of peripheral venous blood from EDTA-containing tubes. The QIAamp DNA Mini Blood Kit (Qiagen) was used for DNA extraction. The quality of the total DNA was checked by electrophoresis on agarose gel and by measuring the absorbance at 260/280 nm, and then, samples were stored at −80 °C until use. The *NOTCH3* mutation p.R544C was identified by PCR-CTPP. DNA was amplified with the primers 5′-GTGGGGTGGAGTGGAAGTAAGTGG (F1) and 5′-GAGCAGTCGTCCACGTTGCA (R1) for the C allele, and 5′-TTGAGGGCACGCTGTGTGATC (F2) and 5′-CTAGATGCACCATTCCCAAACCC (R2) for the T allele. The PCR amplification was performed for 40 cycles (denaturation at 95 °C for 30 s, annealing at 62 °C for 30 s, extension at 72 °C for 1 min, and final extension at 72 °C for 10 min). PCR products of 479 and 216 bp for the TT genotype; 479, 303, and 216 bp for the TC genotype; and 479 and 303 bp for the CC genotype were shown on 2% agarose gel stained with ethidium bromide. Once the sequence variants were identified, additional steps were taken to confirm the sequence changes of the amplicons. A real-time PCR system (SNP Biotechnology) was used for detecting *FV-H1299R*, *MTHFR-C677T*, *MTHFR-A1298C*, *FII-Prothrombin*, *FV-Cambridge*, *PAI1 4G/5G*, and *FXIII Val34Leu*. 

### Ethical Considerations

This study was conducted according to the guidelines of the Declaration of Helsinki and was approved by the ethics committee of Thai Nguyen National Hospital (reference number: #59/HĐĐĐ-BVTWTN#; January 18, 2021). This study obtained informed consent from all participants or their legal representatives and ensured that they understood the study’s purpose, risks, benefits, and procedures.

### Statistical Analysis and HCA

Conventional statistical analyses were performed on our data set, including medical test parameters, using IBM SPSS Statistics 20 (IBM Corp). The relationship between clinicopathological factors and the presence of *NOTCH3* p.R544C, *FV-H1299R*, *MTHFR-C677T*, *MTHFR-A1298C*, *FII-Prothrombin*, *FV-Cambridge*, *PAI1 4G/5G*, and *FXIII Val34Leu* variants were analyzed using the Pearson chi-square test (group size >5) or Fisher exact test (group size ≤5), as appropriate. Bonferroni correction for multiple comparisons was applied. The results have been expressed as percentage or mean (SD).

Following our previous machine learning study [11], our multidimensional analysis was performed in R 4.1.0 (R Project for Statistical Computing). We focused on multivariate statistics, using several algorithms of HCA, matrix correlation, Nagelkerke R square, Kaplan-Meier, and the log-rank test. The chi-square statistics were computed using Yates correction for continuity, with the generation of *P*_yates_. The Pearson or product-moment correlation coefficient is frequently used as the outcome measure for analyses. The Pearson method has an advantage when all or most of the nonzero parameters share the same sign. The Pearson test has been shown to be useful in a genomic setting involving screening for age-related genes, which is our objective [12]. Two alternative criteria include a bias-corrected version of the correlation coefficient (*P*_uncor_) and the Fisher r-to-z transformed correlation coefficient (*P_fisher_*). HCA is a cluster analysis concept that creates a dendrogram hierarchy of clusters. The hierarchical clustering on principal components (HCPC) approach allows the combination of the following 3 standard methods used in multivariate data analysis: principal component methods (principal component analysis [PCA], correspondence analysis [CA], multiple correspondence analysis [MCA], factor analysis of mixed data [FAMD], and multiple factor analysis [MFA]), hierarchical clustering, and partitioning clustering, particularly the k-means method. We calculated the distance between each observation and estimated the cluster distance. The distance between the elements can be complete, single, average, ward, McQuitty, or centroid. The cluster tree was generated by computing the correlation between cophenetic distances and the initial distance data. The number of clusters was determined using k-means, which calculates clustering indexes and reallocates observations to the closest cluster. The k-means computation was optimized using 20 indexes for the PCA cluster plot, which visualizes the best cluster number. PCA is a dimensionality reduction method that is often used to reduce the dimensionality of large data sets by transforming a large set of variables into a smaller set that still contains most of the information in the large set.

## Results

### Overview of the Correlation Between Clinicopathological Factors and the Presence of *NOTCH3* p.R544C, *FV-H1299R*, *MTHFR-C677T*, *MTHFR-A1298C*, *FII-Prothrombin*, *FV-Cambridge*, *PAI1 4G/5G*, and *FXIII Val34Leu*

The study included 100 patients with cerebral infarction from the northern mountainous region of Vietnam. Of the 100 patients, 75 were from the Kinh ethnic group and 25 were from the Tay ethnic group. The average age of the patients was 60.1 years (range: 24-91 years) (Table 2). Of the 100 patients, 22 were aged 24-49 years, 23 were aged 50-59 years, 37 were aged 60-69 years, and 18 were aged 70-91 years.

There were 62 male patients and 38 female patients (male/female ratio of 1.63). The average BMI of the study patients was 22.62 kg/m^2^. Of the 100 patients, 3 had a BMI of <18.5 kg/m^2^, 56 had a BMI of 18.5-22.9 kg/m^2^, 27 had a BMI of 23-24.9 kg/m^2^, and 14 had a BMI of 25-29.9 kg/m^2^. Regarding the risk factors for stroke, of the 100 patients, 70 had hypertension, 44 had a family history of stroke, 31 had a history of smoking, 29 had a history of alcohol consumption, 20 had a history of diabetes, and 35 had a history of stroke (Table 2).

With regard to clinical symptoms, of the 100 patients, 97 had motor paralysis, 95 had difficulty speaking, 72 had mouth distortion, 49 had headache, 41 had numbness, 27 had dizziness or vertigo, 21 had circular muscle disorder, and 8 had nausea or vomiting. Among patients with motor paralysis, 52 had right hemiplegia, 39 had left hemiplegia, and 6 had total paralysis. Among patients with dysphasia, 86 had Broca-type dysphasia and 9 had Wernicke-type dyspraxia (Table 3).

**Figure 1:**
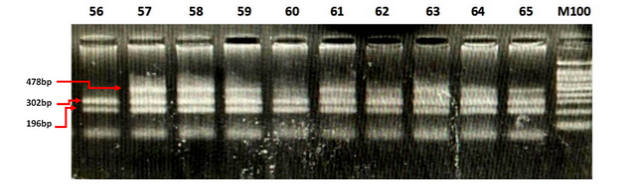
Identification of the *NOTCH3* p.R544C variant by polymerase chain reaction with confronting 2-pair primers.

**Figure 2:**
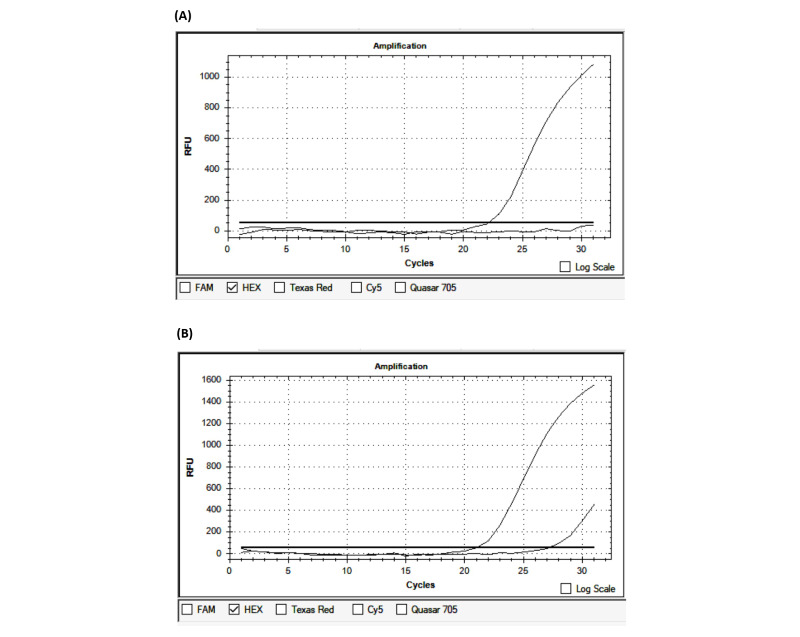
Identification of the FV-Leiden variant by real-time polymerase chain reaction. (A) Wildtype; (B) Heterozygous. RFU: relative fluorescence units.

**Figure 3:**
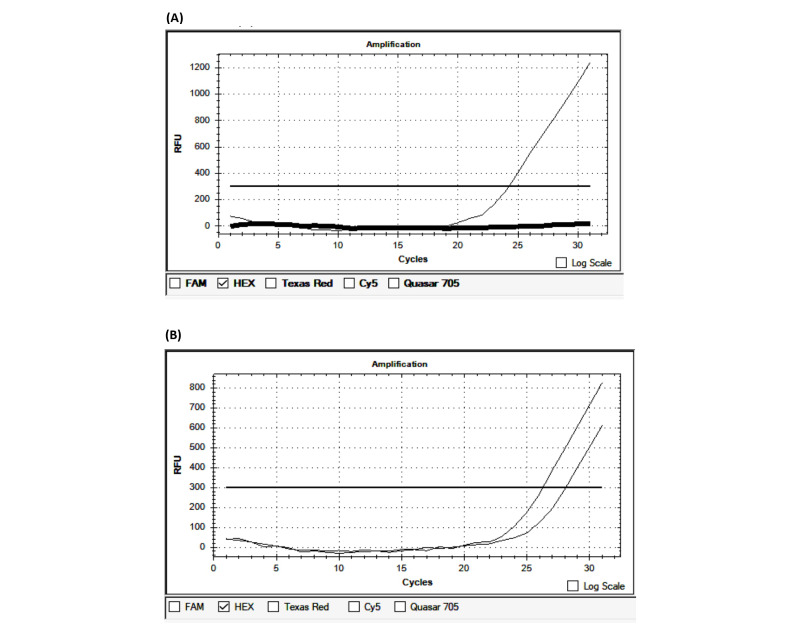
Identification of the *FV-H1299R* variant by real-time polymerase chain reaction. (A) Wildtype; (B) Heterozygous. RFU: relative fluorescence units.

**Figure 4:**
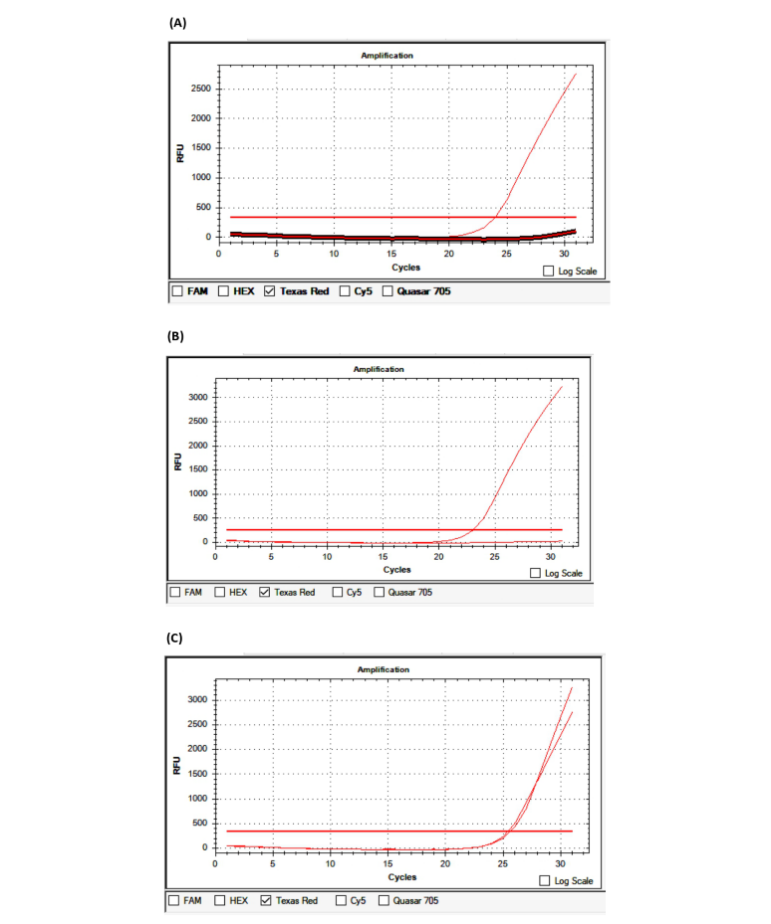
Identification of the *MTHFR-C677T* variant by real-time polymerase chain reaction. (A) Wildtype; (B) Homozygous; (C) Heterozygous. RFU: relative fluorescence units.

**Figure 5:**
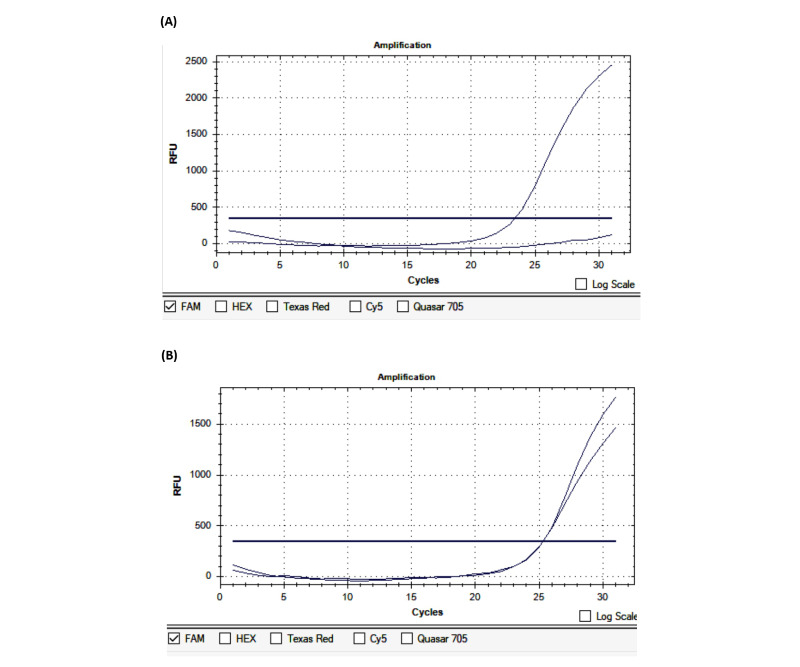
Identification of the *MTHFR-A1298C* variant by real-time polymerase chain reaction. (A) Wildtype; (B) Heterozygous. RFU: relative fluorescence units.

The average time from the onset of the first symptoms to patient admission was 10.94 hours. Of the 100 patients, 33 were admitted within the first 4.5 hours, 26 were admitted from 4.6 to 6 hours, and 41 were admitted outside the first 6 hours. Regarding the blood pressure at admission, the mean systolic blood pressure was 148.6 mmHg and the mean diastolic blood pressure was 88.06 mmHg. The average Glasgow Coma Scale (GCS) score at admission was 14.72. The average National Institutes of Health Stroke Scale (NIHSS) score was 7.14 at admission, 6.71 after 24 hours of hospital treatment, and 3.73 at discharge. The average Rankin score at discharge was 1.52. The average duration of treatment was 10.11 days (Table 4). PCR-CTPP identified *NOTCH3* p.R544C, and other gene variants were detected by real-time PCR (Table 1; Figure 1). The results of real-time PCR for the detection of *FV-H1299R*, *MTHFR-C677T*, *MTHFR-A1298C*, *FII-Prothrombin*, *FV-Cambridge*, *PAI1 4G/5G*, and *FXIII Val34Leu* are presented in Figures 2-5 and Table 2.

**Table 2:** Distribution of patients according to risk factors and genetic variants.

Factors		Value (N=100)
**Gender, n (%)**		
	Male	62 (62)
	Female	38 (38)
**Age group (years), n (%)**		
	24-49	22 (22)
	50-59	23 (23)
	60-69	37 (37)
	70-91	18 (18)
**Age (years)**		
	Mean (SD)	60.14 (12.63)
	Minimum-maximum	24-91
**BMI group (kg/m^2^), n (%)**		
	<18.5	3 (3)
	18.5-22.9	56 (56)
	23.0-24.9	27 (27)
	25.0-29.9	14 (14)
**BMI (kg/m^2^)**		
	Mean (SD)	22.62 (2.49)
	Minimum-maximum	12.4-29.4
**Ethnic group, n (%)**		
	Kinh	75 (75)
	Tay	25 (25)
Smoking history, n (%)		31 (31)
Alcohol consumption, n (%)		29 (29)
Blood pressure, n (%)		70 (70)
Diabetes, n (%)		20 (20)
Brain stroke, n (%)		35 (35)
Brain stroke cases in the family, n (%)		44 (44)
***PAI1 4G/5G* status, n (%)**		
	Wildtype	24 (24)
	Heterozygous	44 (44)
	Homozygous	32 (32)
**FV 1299 status, n (%)**		
	Wildtype	96 (96)
	Heterozygous	4 (4)
	Homozygous	0 (0)
***FV-Cambridge* status, n (%)**		
	Wildtype	100 (100)
	Heterozygous	0 (0)
	Homozygous	0 (0)
**MTHFR 1298 status, n (%)**		
	Wildtype	58 (58)
	Heterozygous	37 (37)
	Homozygous	5 (5)
***FII Prothrombin* status, n (%)**		
	Wildtype	98 (98)
	Heterozygous	1 (1)
	Homozygous	1 (1)
**FV-Leiden status, n (%)**		
	Wildtype	93 (93)
	Heterozygous	7 (7)
	Homozygous	0 (0)
**MTHFR 677 status, n (%)**		
	Wildtype	55 (55)
	Heterozygous	37 (37)
	Homozygous	8 (8)
***FXIII Val34Leu* status, n (%)**		
	Wildtype	98 (98)
	Heterozygous	1 (1)
	Homozygous	1 (1)
***NOTCH3* status, n (%)**		
	Wildtype	6 (6)
	Heterozygous	91 (91)
	Homozygous	3 (3)

**Table 3:** Symptoms at admission.

Symptom		Value (N=100), n (%)
**Vocal issue**		
	No	5 (5)
	Broca type	86 (86)
	Wernicke type	9 (9)
Headache		49 (49)
Dizziness		27 (27)
Nausea or vomiting		8 (8)
Mouth distortion		72 (72)
Circular muscle disorder		21 (21)
Numbness		41 (41)
**Movement paralysis**		
	No	3 (3)
	Paralysis of the right half of the body	52 (52)
	Paralysis of the left half of the body	39 (39)
	Paralysis of the whole body	6 (6)

**Table 4:** Important variables in this study.

Variable				Value (N=100)
**Age (years)**				
	Minimum-maximum			24-91
	Mean (SD)			60.14 (12.63)
**BMI (kg/m^2^)**				
	Minimum-maximum			12.4-29.4
	Mean (SD)			22.62 (2.49)
**Time to hospitalization (h)**				
	Minimum-maximum			1-120
	Mean (SD)			10.94 (15.98)
**Time to hospitalization groups, n (%)**				
	<4.5 h			33 (33)
	4.6-6 h			26 (26)
	>6 h			41 (41)
**Systolic blood pressure (mmHg)**				
	Minimum-maximum			90-210
	Mean (SD)			148.6 (23.66)
**Diastolic blood pressure (mmHg)**				
	Minimum-maximum			60-120
	Mean (SD)			88.06 (9.5)
**Glasgow Coma Scale score**				
	Minimum-maximum			8-15
	Mean (SD)			14.72 (1.06)
**NIHSS^a^ score**				
	**Admission**			
		Minimum-maximum	0-19	
		Mean (SD)	7.14 (4.33)	
	**After 24 h**			
		Minimum-maximum	0-16	
		Mean (SD)	6.71 (4.26)	
	**Discharge**			
		Minimum-maximum	0-16	
		Mean (SD)	3.73 (3.87)	
**Modified Rankin scale score at discharge**				
	Minimum-maximum			0-5
	Mean (SD)			1.52 (1.35)
**Duration of inpatient treatment at the hospital (days)**				
	Minimum-maximum			1-23
	Mean (SD)			10.11 (4.33)

^a^NIHSS: National Institutes of Health Stroke Scale.

**Figure 6:**
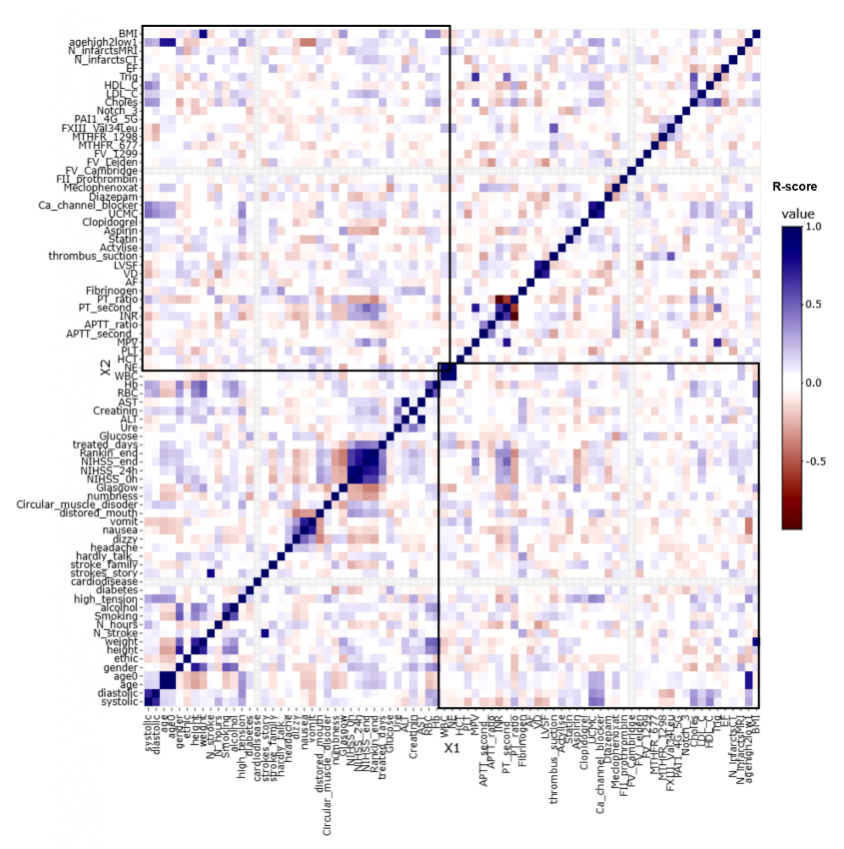
Correlation heatmap of 79 factors in the 100 patients with ischemic stroke.

**Figure 7:**
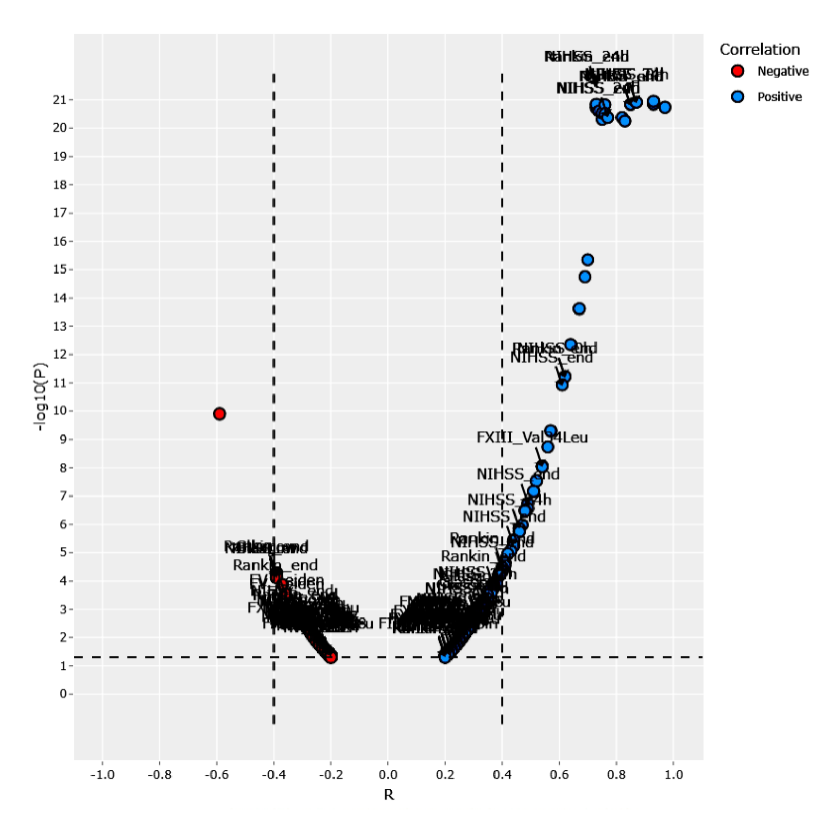
Volcano graph showing the most significant correlation pairs.

Figure 6, Table 1, and Table S1 in Multimedia Appendix 1 provide an overall view of gene prevalence and correlations in both negative and positive genes. We confirmed the presence of significant correlations of *NOTCH3* p.R544C, *FV-H1299R*, *MTHFR-C677T*, *MTHFR-A1298C*, *FII Prothrombin*, *FV-Cambridge*, *PAI1 4G/5G*, and *FXIII Val34Leu* with several factors in patients with ischemic stroke. The Pearson correlation coefficient (R) indicates the extent of the relationship between 2 variables. The relationship strength (effect size) varies according to the threshold of R, with thresholds of 0.5, 0.3, 0, −0.3, and −0.5 for strong positive, moderate positive, weak, moderate negative, and strong negative correlations, respectively(Figure 6; Interactive Graphs 1 [13], 2 [14], 3 [15], and 4 [16]; Table S1 in Multimedia Appendix 1). The volcano graph in Figure 7 shows the most significant correlation pairs, especially those containing the gene mutations mentioned above (Interactive Graph 5 [17]). Overall, a significant medium correlation between the prevalence of gene mutations and other factors was shown in the volcano graph. Compared with other genes, *FXIII Val34Leu* showed the highest positive correlation with thrombus suction ability (R=0.54; *P*<.001; -log_10_*p*=8.03).

In the clustering step, dendrograms were built based on the clustering metric “Euclidean,” and we selected “average” as the most appropriate linkage model, which had the best correlation between cophenetic distances and the original distance data (Table 5).

**Figure 8:**
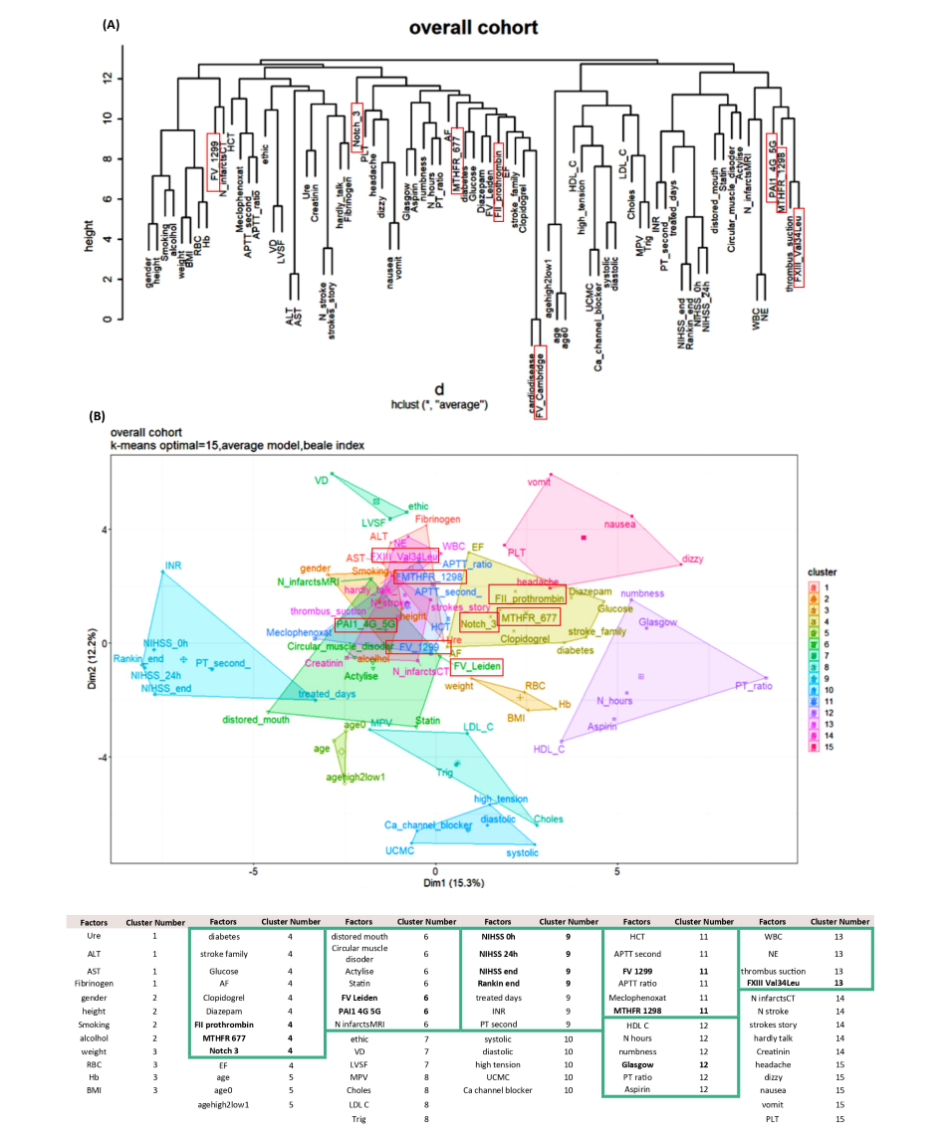
Results of hierarchical cluster analysis on the overall data set. (A) Dendrogram; (B) Principal component analysis map.

**Figure 9:**
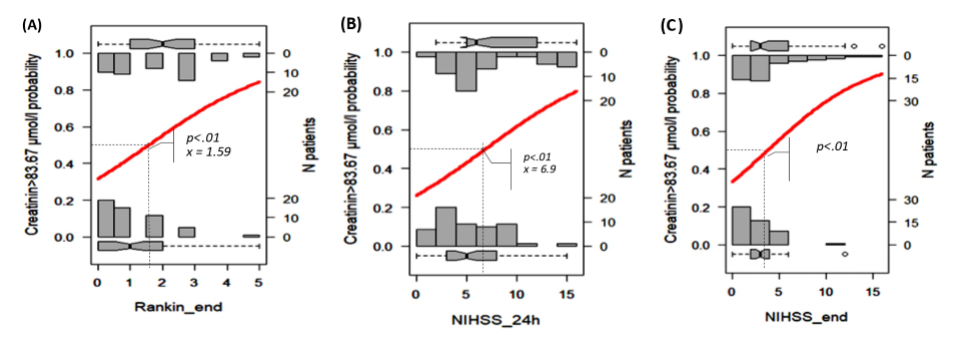
Significant cutpoint 50% of the modified Rankin scale score at discharge (A) and the National Institutes of Health Stroke Scale (NIHSS) scores after 24 hours (B) and at discharge (C) for creatinine levels >83.67 μmol/L.

**Figure 10:**
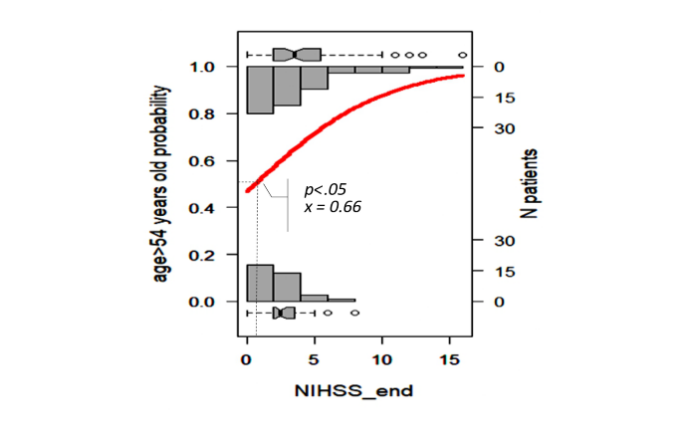
Significant cutpoint 50% of the National Institutes of Health Stroke Scale (NIHSS) score at discharge for patient age >54 years.

**Figure 11:**
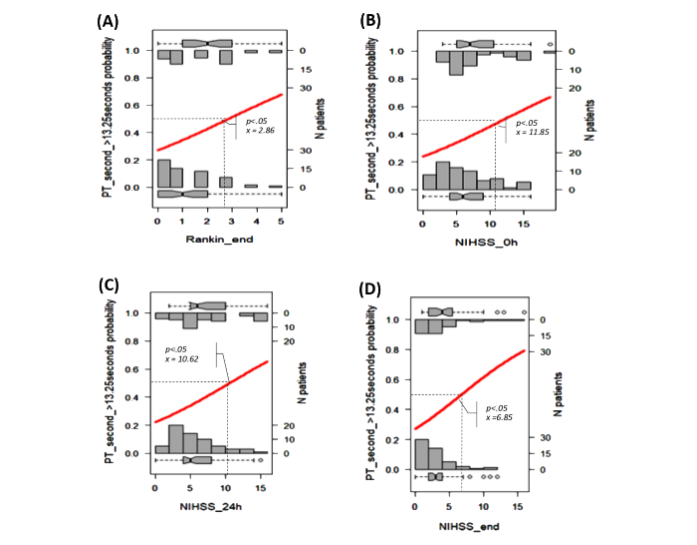
Significant cutpoint 50% of the modified Rankin scale score at discharge (A) and the National Institutes of Health Stroke Scale (NIHSS) scores at admission (B), after 24 hours (C), and at discharge (D) for prothrombin (PT) time >13.25 seconds.

**Figure 12:**
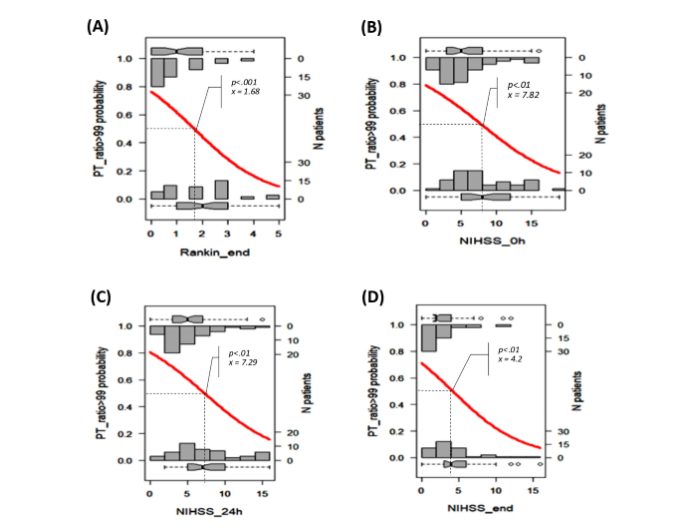
Significant cutpoint 50% of the modified Rankin scale score at discharge (A) and the National Institutes of Health Stroke Scale (NIHSS) scores at admission (B), after 24 hours (C), and at discharge (D) for prothrombin (PT) ratio >99.

**Figure 13:**
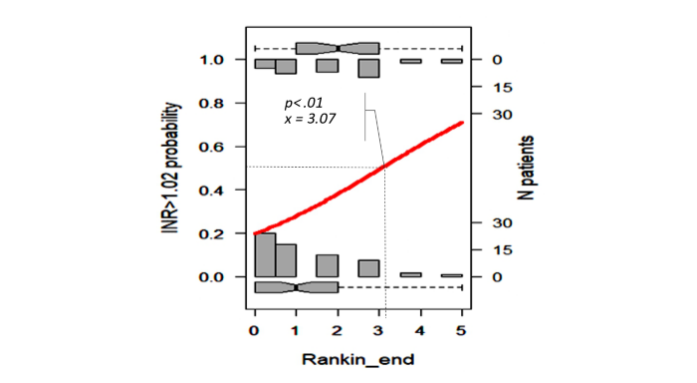
Significant cutpoint 50% of the modified Rankin scale score at discharge for international normalized ratio (INR) >1.02.

**Figure 14:**
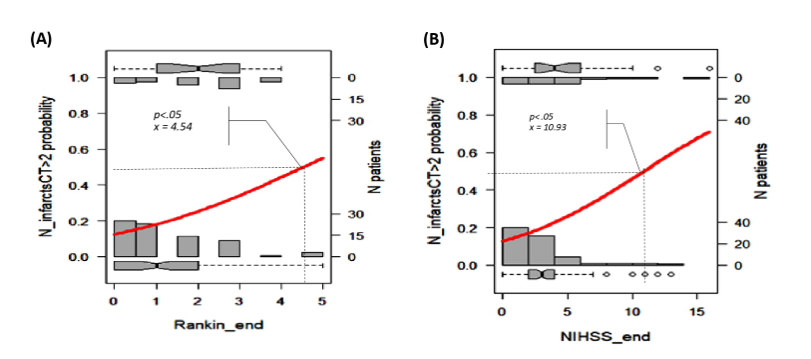
Significant cutpoint 50% of the modified Rankin scale score at discharge (A) and the National Institutes of Health Stroke Scale (NIHSS) score at discharge (B) for the number of infarcts on computed tomography (CT) >2.

**Figure 15:**
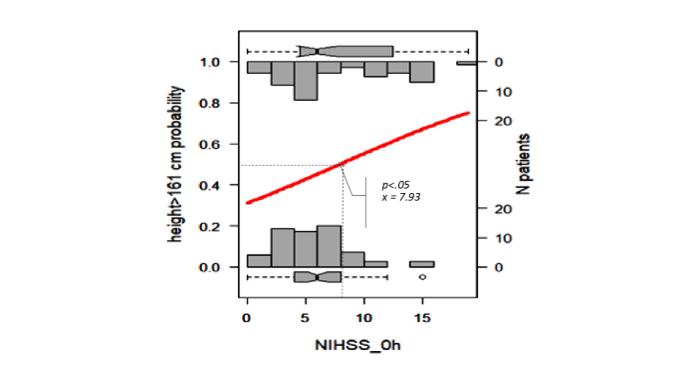
Significant cutpoint 50% of the National Institutes of Health Stroke Scale (NIHSS) score at admission for patient height >161 cm.

**Figure 16:**
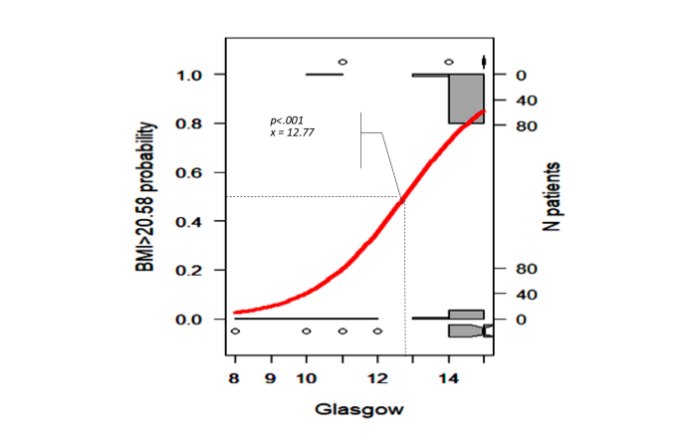
Significant cutpoint 50% of the Glasgow Coma Scale score for patient BMI >20.58 kg/m2.

We selected the results proposed by the Beale method from 20 different index values, and 15 clusters were presented as optimal (Table S2 in Multimedia Appendix 1 [18]). The PCA cluster plot showed that the cluster number mentioned above was the best number to distinguish the clusters and avoid overlap appropriately. The dendrogram and PCA map in Figure 8 complete the overall view of our database, and we can see where the studied genes could combine and might be associated with ischemic stroke outcomes (Interactive Graph 6 [19]). We found several clusters of variants that may have a synchronization impact on the outcomes of ischemic stroke. The PCA map in Figure 8B provides an initial idea of the potential markers that may be important for the ischemic stroke score. For example, the international normalized ratio (INR) and prothrombin (PT) time are in the same cluster with the NIHSS and Rankin scores (cluster 9 in Figure 8B, and clusters 3 and 14 in Interactive Graph 6 [19]), and the GCS score is in the same cluster as the PT ratio (cluster 12 in Figure 8B, and cluster 15 in Interactive Graph 6 [19]). The studied genes were separated into 4 different groups: *FII Prothrombin*, *MTHFR-C677T*, and *NOTCH3* p.R544C were in cluster 4 (Figure 8B; cluster 4 in Interactive Graph 6 [19]); *FV-Leiden* and *PAI1 4G/5G* were in cluster 6 (Figure 8B; cluster 7 in Interactive Graph 6 [19]); *FV-H1299R* and *MTHFR-A1298C* were in cluster 11 (Figure 8B; cluster 1 in Interactive Graph 6 [19]); and *FXIII Val34Leu* was in cluster 13 (Figure 8B; cluster 2 in Interactive Graph 6 [19]). We continued to split the data according to the significant cutpoints of PT time, INR, and ischemic stroke score. We applied the maximally selected rank statistic to define the optimal thresholds of several continuous factors (creatinine, age, PT time and ratio, INR, low-density lipoprotein cholesterol [LDL-C], number of infarcts on CT or MRI, patient height, and mean platelet volume [MPV]) based on the Rankin, NIHSS, and GCS scores and their related symptom statuses, such as numbness, dizziness, gender, circular muscle disorder, mouth distortion, and diabetes status (Table S3 in Multimedia Appendix 1). The optimal cutpoints were as follows: creatinine, 83.67 (SD 9.19) µmol/L; age, 54 (SD 5) years; PT time, 13.25 (SD 0.17) s; INR, 1.02 (SD 0.03); LDL-C, 4.23 (SD 0.89) mmol/L; number of infarcts on CT or MRI, 2; PT ratio, 99.00 (SD 1.96); and MPV, 7.27 (SD 1.09) fL (Table S3 in Multimedia Appendix 1). Using the Nagelkerke method, we assessed which factors could be associated with the cutpoint 50% values of ischemic stroke scores and identified creatinine, age, height, PT time, PT ratio, and number of infarcts on CT. The cutpoint 50% values of the GCS score; modified Rankin scale (mRS) score; and NIHSS scores at admission, after 24 hours, and at discharge were 12.77, 2.86 (SD 1.21), 9.83 (SD 2.85), 7.29 (SD 2.04), and 6.85 (SD 2.90), respectively. These findings allowed appropriate assessment of the possible influences, including those of the genotype variants (Figures 9-16)

**Table 5:** Correlation between cophenetic distances and the original distance data.

Linkage mode	Correlation
Ward.D	0.515
Ward.D2	0.623
Single	0.806
Complete	0.537
Average	0.813
McQuitty	0.694
Median	0.750
Centroid	0.797

### Gene Variants Might be Associated With the Patient Outcome via the Ischemic Stroke Score

We calculated the risk ratios (RRs) and CIs by unconditional maximum likelihood estimation and normal approximation, respectively (Wald), as well as performed minor sample adjustment by the Mantel Haenszel method, generating *P_yates_*, *P_uncor_*, and *P_fisher_*. We grouped these genotype variants following their clusters, which provided the most relevant RR results (Table S4 in Multimedia Appendix 1; Figure 17; Interactive Graph 7 [20]). The detailed RRs for stroke scores are presented in Table S5 in Multimedia Appendix 1. Forest plots were created for clusters 4 (Figures S1-S5 in Multimedia Appendix 2), 6 (Figures S6-S9 in Multimedia Appendix 2), 11 (Figures S10-S13 in Multimedia Appendix 2), and 13 (Figures S14-S17 in Multimedia Appendix 2).

The GCS can be used for head injury, and score ranges are used to describe the injury severity. Scores of 13-15 indicate mild traumatic brain injury, 9-12 indicate moderate traumatic brain injury, and 3-8 indicate severe traumatic brain injury. The risk of experiencing mild traumatic brain injury (cutpoint 50% of GCS was 12.77) was 23% higher in the group of patients without diabetes and with a BMI greater than 20.8 kg/m^2^ as well as *NOTCH3* heterozygous mutation, *MTHFR-C677T*, and FI-Prothrombin than in the other groups (RR=1.23, 95% CI 0.99-1.54; *P_fisher_*=2.68×10^-3^). This risk was 20% lower in the group of patients with BMI less than 20.8 kg/m^2^ and with *MTHFR-A1298C* and *FV-H1299R* wildtype variants than in the other groups (RR=0.79, 95% CI 0.61-1.01; *P_fisher_*=1.72×10^-3^).

The NIHSS quantifies the impairment caused by stroke and aids in planning post-acute care disposition, although it has been intended to assess differences in interventions in clinical trials. A NIHSS score of 0 indicates no stroke symptoms, 1-4 indicates minor stroke, 5-15 indicates moderate stroke, 16-20 indicates moderate to severe stroke, and 21-42 indicates severe stroke. The risk of a NIHSS score at admission greater than 9.83 and a NIHSS score at 24 hours greater than 7.92 (moderate stroke) was higher in the group of patients with age older than 54 years, height shorter than 161 cm, PT time ≤13.25 seconds, PT ratio ≤99, creatinine >83.67 µmol/L, and *FXIII Val34Leu* wildtype than in the other groups (RR=2.72, 95% CI 1.4-5.31 and RR=2.09, 95% CI 1.1-3.93, respectively; *P_fisher_*=2.19×10^-2^ and 8.81×10^-2^, respectively). The risk of a NIHSS score at discharge greater than 6.85 (moderate stroke) was higher in the group of patients with age older than 54 years, height taller than 161 cm, PT time ≤13.25 seconds, PT ratio ≤99, creatinine >83.67 µmol/L, *FII Prothrombin* and *MTHFR-C677T* wildtype, and *NOTCH3* p.R544C heterozygous (RR=4.8, 95% CI 1.53-15.04; *P_fisher_*=3.47×10^-2^).

The mRS is an outcome measure in stroke clinical trials. The mRS assessment is recommended 3 months (90 days) following hospital discharge. The mRS score is assigned as follows: 0, patient has no residual symptoms; 1, patient has no significant disability and has ability to carry out all prestroke activities; 2, patient has remote disability and is incapable of carrying out all prestroke movements but is capable of looking after self without daily help; 3, patient has moderate disability and needs some external help but is capable of walking without the assistance of another individual; 4, patient has moderately severe disability and is incapable of walking or performing physical functions without the aid of another individual; 5, patient has severe disability, is bedridden, shows incontinence, and requires continuous care; 6, patient has passed away (during the hospital stay or after discharge from the hospital); 7, inability to contact the patient or caregiver; and 8, score not achieved or not determined from the medical records. The risk of a mRS score greater than 2.86 (moderate disability) was higher in the group of patients with INR >1.02, PT time >13.25 seconds, PT ratio ≤99, creatinine >83.67 µmol/L, *FXIII Val34Leu* wildtype (in case the number of infarcts on CT was greater than 2), *MTHFR-A1298C* heterozygous/wildtype, and *FV-H1299R* wildtype (RR=3.13, 95% CI 1.6-6.11; *P_fisher_*=2.64×10^-2^).

**Figure 17:**
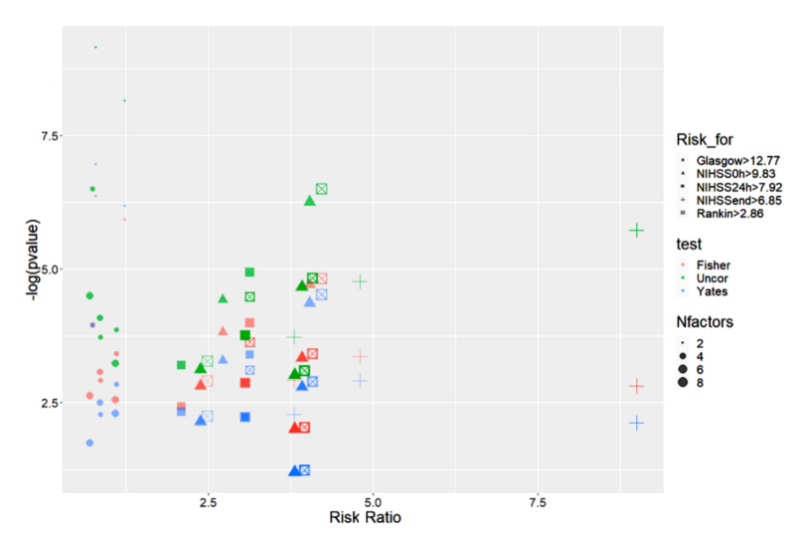
Dot plot of the genotype variants according to their clusters, which provides the most relevant risk ratio results. NIHSS: National Institutes of Health Stroke Scale.

## Discussion

### Principal Findings

Some sophisticated techniques for HCA exploit statistical frameworks called hierarchical models or multilevel models. Hierarchical models are useful in a number of contexts. HCA, which is also known as hierarchical clustering, is a popular method for cluster analysis in big data research and data mining aiming to establish a hierarchy of clusters. As such, HCA attempts to group subjects with similar features into clusters. Clustering is a data science technique in machine learning that groups similar rows in a data set. After running a clustering technique, a new column appears in the data set to indicate the group each row of data fits into the best.

Several gene mutations have been identified as leading causes of cerebral autosomal dominant arteriopathy with subcortical infarcts and leukoencephalopathy (CADASIL), a hereditary disease that causes stroke and other neurological symptoms. CADASIL accounts for up to 5% of all strokes in individuals aged younger than 65 years. The thrombophilia test helps determine the disease’s genetic origin to provide appropriate prevention and treatment measures. Hypercoagulation syndrome may be due to mutations in genes encoding proteins related to blood clotting (thrombophilia). People with hypercoagulable syndrome tend to form blood clots in blood vessels (primarily veins), resulting in stroke, heart attack, repeated miscarriages, and complications during pregnancy (pre-eclampsia, fetal growth retardation, and stillbirth) [21].

In our study, gene variants were assessed to understand how ischemic stroke genetics could interest practitioners and be useful for clinical work. The variants were as follows: *FII Prothrombin*, *FV-Leiden*, *MTHFR-C677T*, *MTHFR-A1298C*, *FV-H1299R*, *PAI1 4G/5G*, *FXIII Val34Leu*, *FV-Cambridge*, *and NOTCH3* p.R544C.

We visualized how these risk factors and genetic elements could affect ischemic stroke outcomes with a hierarchical analysis strategy. Maximally selected rank statistics help to define the optimal thresholds of several continuous factors (creatinine, age, PT time and ratio, INR, LDL-C, number of infarcts on CT or MRI, patient height, and MPV) based on the mRS, NIHSS, and GCS scores and their related symptom statuses, such as numbness, dizziness, gender, circular muscle disorder, mouth distortion, and diabetes status. Their optimal cutpoints fitted with the normal range in both genders. The creatinine level of 83.67 (SD 9.19) µmol/L is consistent with the usual results of 0.7 to 1.3 mg/dL (61.9 to 114.9 µmol/L) for men and 0.6 to 1.1 mg/dL (53 to 97.2 µmol/L) for women [22]. Our age threshold was 54 (SD 5) years, which is consistent with the findings worldwide, with aging being the most robust nonmodifiable risk factor for incident stroke (risk doubles every 10 years after the age of 55 years) [23]. Assessment of the PT time is recommended for the administration of recombinant tissue-plasminogen activator (rt-PA) in stroke [24]. The standard range of the PT time is 10 to 13 seconds. The usual INR for a healthy individual is 1.1 or below, and the therapeutic range for most patients on vitamin K antagonists is 2.0 to 3.0. An augmented PT/INR for patients on vitamin K antagonists may suggest a super-therapeutic status and will need prescription dose adjustments to control bleeding [25]. In our study, the calculated baseline PT time was 13.25 (SD 0.17) and INR was 1.02 (SD 0.03), which confirmed cases of moderate outcomes. Data on the association between BMI and stroke are scarce. Individuals with a BMI of 18.5 to 24.9 kg/m^2^ are considered to have a healthy weight. Our calculated baseline BMI was 20.85 kg/m^2^, and it was associated with genetic factors that influence the GCS score. 

According to the Nagelkerke method, the cutpoint 50% values of the mRS score and NIHSS scores at admission, after 24 hours, and at discharge were 2.86 (SD 1.21), 9.83 (SD 2.85), 7.29 (SD 2.04), and 6.85 (SD 2.90), respectively, which were consistent with the moderate outcomes of our patients. We found that the MTHFR and *NOTCH3* p.R544C variants may influence stroke severity in patients with specific conditions of PT, creatinine, INR, and BMI. 

The MTHFR gene provides instructions for the human body to make the MTHFR protein, which helps the body process folate, which is important for forming DNA and modifying proteins. The most common variant of the MTHFR gene is *MTHFR-C677T* [26]. This mutation causes a reduction in the capacity to create L-methylfolate. *MTHFR-A1298C* single-nucleotide polymorphism has also been suggested to have an impact on MTHFR enzyme activity but to a lesser extent than the *MTHFR-C677T* polymorphism. They have been recently shown to be associated with ischemic stroke [27].

CADASIL is an autosomal dominant inherited vasculopathy and is the most common single‐gene disorder causing stroke, with more than 200 different *NOTCH3* p.R544C mutations in patients worldwide, indicating that CADASIL has considerable genetic heterogeneity. The defective 33‐exon *NOTCH3* p.R544C gene is located on chromosome 19, which typically impacts the number of highly conserved cysteine residues among the epidermal growth factor–like repeat domain [28].

HCA is attractive for exploratory high-throughput data because it provides a convenient approach to visualize the similarities of variables and infer the grouping of variables based on the dendrogram structure. Hence, HCA facilitates the interpretation of the data of the microbiome and other omics. Importantly, bi-clustering (2-way clustering), a particular approach of HCA, can incorporate a correlation method (eg, Spearman rank correlation) to cluster rows and columns of the data matrix simultaneously. Thus, bi-clustering can find features (microbial taxa, genes, metabolites, etc) that correlate only in a subset of objects but not in the rest of the data set [29]. In this study, we clearly identified the role and interaction of risk factors that influence stroke progression. Genetic mutations become significant in a small range of strongly correlated factors through a PCA plot.

Stroke has multiple modifiable and nonmodifiable risk factors and represents a leading cause of death globally. Understanding the complex interplay of stroke risk factors is thus not only a scientific necessity but also a critical step toward improving global health outcomes [30].

### Limitations

We found that 3 of the 9 gene variants had significant RRs. Data settings could help to work with both qualitative and numerical data simultaneously. The main advantage of the HCA clustering concept is the display of possible correlations between several factors to provide reference markers that are useful for diagnostic control and to improve outcome prevention. It was beneficial to identify the association between genetic characteristics and clinical outcomes, which usually requires several in vitro studies; however, there were some constraints. It is critical to clean and prepare the data set because HCA and k-means cannot operate with missing or noisy data. We must combine and validate the data with k-means, which provides several options for the optimal cluster number to produce a PCA cluster plot and define the principal component position. Since our data had various kinds of information, it was challenging to calculate the distance matrix in HCA and k-means.

### Conclusions

The existence of conventional vascular risk factors may prevent clinicians from suspecting the possibility of gene mutations in stroke patients, especially among those with underlying atrial fibrillation or extensive artery atherosclerosis. In this study, a more specific population was chosen. It is interesting that although there are many genes linked to increased atrial fibrillation risk, not all of them are associated with ischemic stroke risk, which might be because those gene variants are too rare to detect their impacts on stroke risk. Nevertheless, in the future, the identification of a linkage between some of those genes and ischemic stroke could be a significant game changer in the field of stroke prevention. Moreover, with the detection of stroke risk loci, more information can be gained on their impacts and interconnections, and the precision of stroke scores might increase.

## Appendix

Multimedia Appendix 1 Supplementary results.

Multimedia Appendix 2 Forest plots.

## Abbreviations

CADASIL: cerebral autosomal dominant arteriopathy with subcortical infarcts and leukoencephalopathy
CT: computed tomography
CTA: computed tomography angiography
CTPP: confronting 2-pair primers
GCS: Glasgow Coma Scale
HCA: hierarchical cluster analysis
HDAC: histone deacetylase
INR: international normalized ratio
LDL-C: low-density lipoprotein cholesterol
MPV: mean platelet volume
MRI: magnetic resonance imaging
mRS: modified Rankin scale
NIHSS: National Institutes of Health Stroke Scale
PCA: principal component analysis
PCR: polymerase chain reaction
PT: prothrombin
RR: risk ratio

## Data Availability

The data sets used or analyzed during this study are available from the corresponding author on reasonable request. We however cannot provide personal information or data that contain the identity of patients in any form.
