# Supplementary material for: The Roles of NOTCH3 p.R544C and Thrombophilia Genes in Vietnamese Patients With Ischemic Stroke: Study Involving a Hierarchical Cluster Analysis
Source: JMIR Bioinform Biotechnol. 2024 May 7;5:e56884. doi: 10.2196/56884 (PMC11135231; doi:10.2196/56884)
Supplement: Multimedia Appendix 1 [file bioinform_v5i1e56884_app1.pdf]

Multimedia Appendix 1. Supplementary results.

Table S1. Correlation results between factors.

**Factor I**	**Factor II**	**Factor III**	**R(I-II)**	**p_{value}(I-II)**	**R(II-III)**	**p_{value} (II-III)**
**FV_1299**	high_tension	age	-0.23	$4.89x10^{-2}$	0.24	$4.06x10^{-2}$
		diastolic			0.43	$9.97x10^{-5}$
		N_stroke			0.23	$4.69x10^{-2}$
		systolic			0.31	$6.75x10^{-3}$
		agehigh2low1			0.34	$2.81x10^{-3}$
		Aspirin			0.23	$4.69x10^{-2}$
		Ca_channel_blocker			0.42	$1.68x10^{-4}$
		Choles			0.31	$6.83x10^{-3}$
		Circular_muscle_disoder			0.28	$1.66x10^{-2}$
		Creatinin			0.26	$2.46x10^{-2}$
		PT_ratio			0.24	$3.92x10^{-2}$
		strokes_story			0.30	$8.42x10^{-3}$
		thrombus_suction			-0.29	$1.29x10^{-2}$
		UCMC			0.51	$2.78x10^{-6}$
**Factor I**	**Factor II**	**Factor III**	**R(I-II)**	**p(I-II)**	**R(II-III)**	**p(II-III)**
**FV_Leiden**	APTT_ratio	APTT_second_	-0.32	$4.87x10^{-3}$	0.34	$2.66x10^{-3}$
		nausea			-0.25	$3.10x10^{-2}$
		PT_ratio			-0.23	$4.70x10^{-2}$
	Diazepam	Glucose	0.28	$1.57x10^{-2}$	0.23	$4.41x10^{-2}$
		LVSF			-0.27	$1.80x10^{-2}$
		NIHSS_0h			-0.24	$3.90x10^{-2}$
		NIHSS_24h			-0.23	$4.48x10^{-2}$
		Meclophenoxat			-0.41	$3.00x10^{-4}$
		**MTHFR_1298**			-0.24	$4.16x10^{-2}$
	hardly_talk_	gender	0.24	$4.13x10^{-2}$	0.28	$1.40x10^{-2}$
		weight			0.23	$4.70x10^{-2}$
		Fibrinogen			0.31	$6.00x10^{-3}$
		Statin			0.33	$3.97x10^{-3}$
**Factor I**	**Factor II**	**Factor III**	**R(I-II)**	**p(I-II)**	**R(II-III)**	**p(II-III)**
**FXIII_Val34Leu**	thrombus_suction	APTT_second_	0.59	$3.17x10^{-8}$	-0.31	$6.73x10^{-3}$
	**MTHFR_1298**	thrombus_suction	0.33	$4.43x10^{-3}$	0.26	$2.29x10^{-2}$

		ethic			0.28	$1.47x10^{-2}$
		Meclophenoxat			0.30	$8.98x10^{-3}$
		Diazepam			-0.24	$4.16x10^{-2}$
		Actylise			-0.23	$4.80\ x10^{-2}$
		thrombus_suction			0.26	$2.29x10^{-2}$
	NE	alcolhol	0.31	$6.11x10^{-3}$	0.23	$4.86x10^{-2}$
		Circular_muscle_disoder			0.30	$1.02x10^{-2}$
		gender			0.28	$1.41x10^{-2}$
		Glasgow			-0.24	$3.69x10^{-2}$
		Glucose			0.25	$2.84x10^{-2}$
		Hb			0.26	$2.39x10^{-2}$
		vomit			0.29	$1.24x10^{-2}$
		WBC			0.91	<0.00001
		N_infarctsMRI			0.24	$3.81x10^{-2}$
	WBC	Circular_muscle_disoder	0.30	$8.54x10^{-3}$	0.29	$1.29x10^{-2}$
		Hb			0.28	$1.43x10^{-2}$
		Smoking			0.26	$2.48x10^{-2}$
		Meclophenoxat			-0.26	$2.56x10^{-2}$
		N_infarctsMRI			0.27	$2.03x10^{-2}$
	INR	dizzy	0.29	$1.05x10^{-2}$	-0.35	$2.03x10^{-3}$
		headache			-0.31	$6.11x10^{-3}$
		NIHSS_0h			0.37	$1.10x10^{-3}$
		NIHSS_24h			0.39	$5.50x10^{-4}$
		NIHSS_end			0.25	$2.97x10^{-2}$
		Rankin_end			0.29	$1.15x10^{-2}$
		systolic			-0.27	$1.99x10^{-2}$
		PT_ratio			-0.95	<.00001
		PT_second_			0.58	$5.87x10^{-8}$
		thrombus_suction			0.27	$1.97x10^{-2}$
	diastolic	systolic	-0.28	$1.49x10^{-2}$	0.72	$2.66x10^{-13}$
		Hb			0.31	$7.48x10^{-3}$
		HDL_C			0.35	$1.87x10^{-3}$
		high_tension			0.43	$9.97x10^{-5}$
		RBC			0.32	$5.72x10^{-3}$
		UCMC			0.37	$1.21x10^{-3}$
	PT_ratio	APTT_ratio	-0.27	$2.09x10^{-2}$	-0.23	$4.70x10^{-2}$
		dizzy			0.36	$1.65x10^{-3}$

		ethic			-0.26	$2.43x10^{-2}$
		gender			-0.23	$4.96x10^{-2}$
		headache			0.31	$7.14x10^{-3}$
		high_tension			0.24	$3.92x10^{-2}$
		INR			-0.95	<0.00001
		nausea			0.23	$4.55x10^{-2}$
		NIHSS_0h			-0.36	$1.54x10^{-3}$
		NIHSS_24h			-0.35	$2.23x10^{-3}$
		PT_second_			-0.51	$2.93x10^{-6}$
		Rankin_end			-0.28	$1.54x10^{-2}$
		systolic			0.27	$2.01x10^{-2}$
		Choles			0.32	$5.54x10^{-3}$
		thrombus_suction			-0.30	$1.02x10^{-2}$
		VD			-0.25	$3.14x10^{-2}$
**Factor I**	**Factor II**	**Factor III**	**R(I-II)**	**p(I-II)**	**R(II-III)**	**p(II-III)**
**MTHFR_1298**	thrombus_suction	PT_ratio	0.26	$2.29x10^{-2}$	-0.30	$1.02x10^{-2}$
		high_tension			-0.29	$1.29x10^{-2}$
		INR			0.27	$1.97x10^{-2}$
		headache			-0.25	$3.05x10^{-2}$
		EF			-0.24	$3.67x10^{-2}$
**Factor I**	**Factor II**	**Factor III**	**R(I-II)**	**p(I-II)**	**R(II-III)**	**p(II-III)**
**MTHFR_677**	Actylise	NIHSS_0h	-0.23	$4.80x10^{-2}$	0.24	$4.17x10^{-2}$
		systolic			0.24	$3.91x10^{-2}$
		weight			0.28	$1.50x10^{-2}$
**Factor I**	**Factor II**	**Factor III**	**R(I-II)**	**p(I-II)**	**R(II-III)**	**p(II-III)**
**Notch_3**	Statin	hardly_talk_	-0.37	$1.21x10^{-3}$	0.33	$3.97x10^{-3}$
	height	gender	-0.29	$1.24x10^{-2}$	0.72	$2.70x10^{-13}$
		Actylise			0.30	$8.32x10^{-3}$
		AF			-0.33	$3.85x10^{-3}$
		alcolhol			0.50	$5.78x10^{-6}$
		Creatinin			0.28	$1.57x10^{-2}$
		Hb			0.35	$2.39x10^{-3}$
		LVSF			0.27	$2.01x10^{-2}$
		N_infarctsMRI			0.31	$7.19x10^{-3}$
		NIHSS_0h			0.30	$9.27x10^{-3}$
		NIHSS_24h			0.23	$4.35x10^{-2}$
		PLT			-0.26	$2.57x10^{-2}$

		RBC			0.26	$2.34x10^{-2}$
		Smoking			0.39	$5.14x10^{-4}$
		weight			0.67	$3.68x10^{-11}$
	Actylise	height	-0.29	$1.25x10^{-2}$	0.30	$8.32x10^{-3}$
	Aspirin	ALT	0.28	$1.58x10^{-3}$	-0.26	$2.33x10^{-2}$
		AST			-0.31	$7.36x10^{-3}$
		gender			-0.23	$4.95x10^{-2}$
		Glasgow			0.24	$3.95x10^{-2}$
		high_tension			0.23	$4.69x10^{-2}$
		NIHSS_0h			-0.35	$2.22x10^{-3}$
		NIHSS_24h			-0.41	$2.45x10^{-4}$
		NIHSS_end			-0.24	$3.61x10^{-2}$
		Rankin_end			-0.33	$3.83x10^{-3}$
		Ure			-0.23	$4.75x10^{-2}$
	APTT_second_	APTT_ratio	-0.25	$2.95x10^{-2}$	0.34	$2.66x10^{-3}$
		Meclophenoxat			0.31	$6.69x10^{-3}$
		thrombus_suction			-0.31	$6.73x10^{-3}$
	gender	alcolhol	-0.23	4.4610^{-2}	0.47	$2.06x10^{-5}$
		Aspirin			-0.23	$4.95x10^{-2}$
		Choles			-0.27	$2.05x10^{-2}$
		Creatinin			0.43	$1.38x10^{-4}$
		hardly_talk_			0.28	$1.40x10^{-2}$
		Hb			0.41	$2.79x10^{-4}$
		height			0.72	$2.70x10^{-13}$
		LVSF			0.28	$1.64x10^{-2}$
		N_infarctsMRI			0.37	$9.66x10^{-4}$
		NE			0.28	$1.41x10^{-2}$
		NIHSS_0h			0.29	$1.22x10^{-2}$
		NIHSS_24h			0.28	$1.41x10^{-2}$
		NIHSS_end			0.26	$2.68x10^{-2}$
		PT_ratio			-0.23	$4.96x10^{-2}$
		Rankin_end			0.32	$4.74x10^{-3}$
		RBC			0.28	$1.50x10^{-2}$
		Smoking			0.53	$1.04x10^{-6}$
		VD			0.26	$2.22x10^{-2}$
		weight			0.50	$3.85x10^{-6}$

Table S2. Results of clustering imputation.

Methods	Number clusters	Value Index
kl	3	3.0285
ch	77	185.7114
hartigan	77	Inf
cindex	77	0.1155
db	77	0.051
silhouette	77	0.9932
duda	3	0.9519
pseudot2	3	2.9293
beale	15	-11.4778
ratkowsky	-Inf	0
ball	3	33.8538
ptbiserial	6	0.518
gap	2	0.2312
frey	2	1.1939
mcclain	2	0.167
gamma	71	1
gplus	71	0
tau	5	473.3775
dunn	77	1.6721
sdindex	55	1.8494
dindex	77	0.0001

Abbreviations of clustering methods [18]: CH (Calinski and Harabasz 1974), CCC (Sarle 1983), Pseudot2 (Duda and Hart 1973), KL (Krzanowski and Lai 1988), Gamma (Baker and Hubert 1975), Gap (Tibshirani et al. 2001), Silhouette (Rousseeuw 1987), Hartigan (Hartigan 1975), Cindex (Hubert and Levin 1976), DB (Davies and Bouldin 1979), Ratkowsky (Ratkowsky and Lance 1978), Scott (Scott and Symons 1971), Marriot (Marriot 1971), Ball (Ball and Hall 1965), Trcovw (Milligan and Cooper 1985), Tracew (Milligan and Cooper 1985), Friedman (Friedman and Rubin 1967), Rubin (Friedman and Rubin 1967), Dunn (Dunn 1974).

Table S3. Maximally selected rank statistics to define the optimal thresholds of several continuous factors following the ischemic stroke score and discrete elements.

A

**Rankin at the hospital discharge**												
**Event**	**numbness**		**dizzy**		**gender**		**Circular muscle disoder**		**distored mouth**		**MEDIAN**	**SD**
**Factors**	**cutpoint**	**statistic**	**cutpoint**	**statistic**	**cutpoint**	**statistic**	**cutpoint**	**statistic**	**cutpoint**	**statistic**		
Creatinin (µmol/l)	85.170	3.258	101.350	2.245	102.400	3.135	103.500	3.441	85.170	3.606	101.350	9.477
Age (years old)	54.000	3.374	44.000	2.022	54.000	2.977	51.000	1.143	54.000	2.290	54.000	4.336
PT second (s)	12.700	3.752	13.100	3.177	13.100	3.295	13.100	2.989	13.100	3.304	13.100	0.179
INR	1.000	3.696	1.000	4.047	1.010	3.274	1.000	2.797	1.010	3.370	1.000	0.005
LDL C (mmol/l)	4.590	1.592	2.130	2.501	4.590	2.487	4.740	2.365	2.210	1.913	4.590	1.355
N of infarcts via CT	2.000	2.858	2.000	2.466	2.000	1.578	2.000	2.624	2.000	1.824	2.000	0.000
PT ratio	97.300	3.679	97.300	3.887	97.000	3.504	99.000	2.752	97.000	3.525	97.300	0.841

B

**NIHSS at the hospital admission**														
**Event**	**numbness**		**dizzy**		**gender**		**Circular muscle disoder**		**distored mouth**		**diabete**		**MEDIAN**	**SD**
**Factors**	**cutpoint**	**statistic**	**cutpoint**	**statistic**	**cutpoint**	**statistic**	**cutpoint**	**statistic**	**cutpoint**	**statistic**	**cutpoint**	**statistic**		
Creatinin (µmol/l)	82.170	2.667	75.640	1.819	82.170	1.434	82.170	2.059	82.170	3.328			82.170	2.920
Age (years old)	54.000	2.788	44.000	1.400	54.000	1.400	44.000	1.652	44.000	0.792			44.000	5.477
PT second (s)	13.600	2.936	13.400	3.273	13.400	2.670	14.000	1.358	12.400	2.208			13.400	0.590
INR	1.010	2.988	1.000	3.826	1.060	2.428	0.970	1.593	0.980	2.285	0.990	2.637	0.995	0.032
LDL C (mmol/l)	2.610	1.562	2.130	1.712	3.620	2.001	4.740	1.724	2.230	1.663			2.610	1.106
N of infarcts via CT	2.000	2.047	2.000	1.797	1.000	1.264	2.000	2.235	1.000	1.729			2.000	0.548
PT ratio	103.000	3.050	97.300	3.592	90.300	2.295	103.000	1.627	102.000	2.715			102.000	5.466
Height (cm)			153.000	2.659	155.000	0.982	169.000	2.142	161.000	2.533	162.000	0.951	161.000	6.325

N infarcts via MRI			2.000	2.203	2.000	0.356	2.000	0.851	2.000	1.399	2.000	1.796	2.000	0.000

C

**NIHSS after 24h**												
**event**	**numbness**		**dizzy**		**gender**		**Circular muscle disoder**		**distored mouth**		**MEDIAN**	**SD**
**Factors**	**cutpoint**	**statistic**	**cutpoint**	**statistic**	**cutpoint**	**statistic**	**cutpoint**	**statistic**	**cutpoint**	**statistic**		
Creatinin (µmol/l)	82.170	2.726	101.350	2.307	82.170	1.707	82.170	2.024	82.170	3.425	82.170	8.578
Age (years old)	54.000	3.061	44.000	1.666	55.000	2.172	59.000	0.821	54.000	1.424	54.000	5.541
PT second (s)	12.900	3.054	13.400	3.426	13.400	3.423	13.300	2.373	13.400	3.011	13.400	0.217
INR	0.950	3.070	1.000	3.877	1.040	3.052	1.060	1.767	1.070	2.784	1.040	0.049
LDL C (mmol/l)	2.610	1.668	2.130	2.118	4.160	3.095	4.740	2.730	4.160	2.406	4.160	1.125
N of infarcts via CT	2.000	2.540	2.000	2.567	2.000	1.741	2.000	2.850	2.000	2.438	2.000	0.000
PT ratio	114.500	3.302	97.300	3.705	93.000	2.686	99.000	1.725	99.000	2.615	99.000	8.170
MPV					6.000	1.111	6.600	1.144	6.500	1.261	6.500	0.321

D

**NIHSS at the hospital admission**												
**Event**	**numbness**		**dizzy**		**gender**		**Circular muscle disoder**		**distored mouth**			
**Factors**	**cutpoint**	**statistic**	**cutpoint**	**statistic**	**cutpoint**	**statistic**	**cutpoint**	**statistic**	**cutpoint**	**statistic**	**MEDIAN**	**SD**
Creatinin (µmol/l)	77.700	2.608	101.350	2.024	100.500	1.053	77.830	1.755	85.170	3.120	85.170	11.733
Age (years old)	54.000	3.295	44.000	2.083	54.000	3.089	59.000	0.857	54.000	2.052	54.000	5.477
PT second (s)	12.700	3.380	13.100	2.866	13.600	2.895	13.100	1.689	13.800	2.627	13.100	0.439
INR	1.010	2.923	1.000	3.657	1.070	3.045	1.060	1.529	1.070	2.778	1.060	0.034
LDL C (mmol/l)	2.610	1.779	2.130	2.290	4.310	3.056	4.740	2.701	4.740	2.203	4.310	1.244
N of infarcts via CT	2.000	2.552	2.000	2.310	1.000	1.506	2.000	2.736	2.000	2.293	2.000	0.447
PT ratio	114.500	3.346	97.300	3.410	92.000	2.412	99.000	1.179	99.000	2.207	99.000	8.410
MPV (fL)			9.600	1.951					6.500	1.190	8.050	2.192

E

**Glasgow**						
**Event**	**Circular muscle disoder**		**ethic**			
**Factors**	**cutpoint**	**statistic**	**cutpoint**	**statistic**	**MEDIAN**	**SD**
BMI	20.576	3.513	20.576	3.422	20.576	0.000

F

	**Median**	**SD**
Creatinin (µmol/l)	83.670	9.199
Age (years old)	54.000	5.000
PT second (s)	13.250	0.173
INR	1.020	0.031
LDL C (mmol/l)	4.235	0.890
N of infarcts via CT	2.000	0.000
PT ratio	99.000	1.955
Height (cm)	161.000	6.325
N infarcts via MRI	2.000	0.000
MPV (fL)	7.275	1.096
BMI	20.576	0.000

Table S4. The most relevant factors related with diagnostic and stroke outcomes (ischemic stroke score) according to risk ratio results.

		Glasgow >12.77		NIHSS at admission >9.83±2.85	NIHSS at 24h >7.92±2.04	NIHSS at discharged >6.85±2.90	Rankin at discharged >2.86±1.21			
BMI	≤20.58		✓							
	>20.58	✓								
INR	≤1.02±0.03									
	>10.2±0.03						✓	✓	✓	✓
PT time	≤13.25±0.17 seconds									
	>13.25±0.17 seconds			✓	✓	✓	✓	✓	✓	✓
PT ratio	≤99±1.96			✓	✓	✓	✓	✓	✓	✓
	>99±1.96									
Creatinin	≤83.67±9.2 µmol/l									
	>83.67±9.2 µmol/l			✓	✓	✓	✓	✓	✓	✓
N of infarcts via CT	≤2							✓	✓	✓
	>2						✓			
FII prothrombin	wildtype	✓				✓				
	heterozygous									
	homozygouszygous									
MTHFR 677	wildtype	✓								
	heterozygous					✓				
	homozygouszygous									
MTHFR 1298	wildtype		✓						✓	✓
	heterozygous							✓		
	homozygouszygous									
FV1299	wildtype		✓					✓	✓	
	heterozygous									
	homozygouszygous									
Notch3	wildtype									
	heterozygous	✓				✓				
	homozygouszygous									
diabetes	yes									
	no	✓								
FXIII Val34Leu	wildtype			✓	✓	✓	✓			✓
	heterozygous									

	homozygouszygous									
age	≤54±5y.o									
	>54±5y.o			✓	✓	✓				
height	≤161±6.3cm			✓	✓					
	>161±6.3cm					✓				
RR[95%CI]		1.23 [0.99,1.54]	0.79 [0.61,1.01]	2.72 [1.4,5.31]	2.09 [1.1,3.95]	4.8 [1.53,15.04]	3.96 [2.82,5.56]	3.13 [1.6,6.11]	3.13 [1.6,6.11]	3.13 [1.6,6.11]
p_{fisher}		$2.68x10^{-3}$	$1.72 x10^{-3}$	$2.19 x10^{-2}$	$8.81x10^{-2}$	$3.47 x10^{-2}$	1.30 x10-01	$2.64 x10^{-2}$	$2.64 x10^{-2}$	$2.64 x10^{-2}$
p_{yates}		$2.07 x10^{-3}$	$9.51 x10^{-4}$	$3.73 x10^{-2}$	$9.72x10^{-2}$	$5.46 x10^{-2}$	2.91 x10-01	$4.47 x10^{-2}$	$4.47 x10^{-2}$	$4.47 x10^{-2}$
p_{uncor}		$2.90 x10^{-4}$	$1.07 x10^{-4}$	$1.20 x10^{-2}$	$4.07 x10^{-2}$	$8.49 x10^{-3}$	$4.50 x10^{-2}$	$1.13 x10^{-2}$	$1.13 x10^{-2}$	$1.13 x10^{-2}$

Table S5. Details of the most relevant factors related with diagnostic and stroke outcomes (based on the ischemic stroke score) according to risk ratio results.

(A) Risk ratio for Glasgow >12.77

Factor1	Factor2	Factor3	Factor4	Factor5	Factor6	Factor7	Factor8	Factor9	Studied group (N)		Control group (N)		p_{fisher}	p_{yates}	p_{uncor}	RR	lower	upper
									Glasgow >12.77	Glasgow ≤12.77	Glasgow >12.77	Glasgow ≤12.77						
INR >1.02	PTsecond >13.25seconds	PTratio ≤99	Creatinin >83.67umol/l	NinfarctsCT ≤2	FXIIIVal34Leu homozygous				0	1	26	73	1.30 x10-01	2.91 x10-01	4.50 x10^{-2}	3.96	2.82	5.56
INR >1.02	PTsecond ≤13.25seconds	PTratio ≤99	Creatinin ≤83.67umol/l	NinfarctsCT >2	FXIIIVal34Leu wt				0	1	26	73	1.30 x10-01	2.91 x10-01	4.50 x10^{-2}	3.96	2.82	5.56
INR >1.02	PTsecond ≤13.25seconds	PTratio ≤99	Creatinin ≤83.67umol/l	NinfarctsCT ≤2	FXIIIVal34Leu hetero				0	1	26	73	2.64 x10^{-2}	4.47 x10^{-2}	1.13 x10^{-2}	3.13	1.6	6.11
BMI >20.58	FIIprothrombin wt	MTHFR677 wt	Notch3 hetero	nondiabetes					79	1	16	4	2.68 x10^{-3}	2.07 x10^{-3}	2.90 x10^{-4}	1.23	0.99	1.54
BMI ≤20.58	FIIprothrombin wt	MTHFR677 homozygous	Notch3 hetero	nondiabetes					44	0	51	5	3.26 x10^{-2}	5.80 x10^{-2}	2.10 x10^{-2}	1.1	1.01	1.19
BMI ≤20.58	FVLeiden wt	PAI14G5G hetero	FVCambridge wt						37	0	58	5	7.72 x10^{-2}	9.98 x10^{-2}	3.94 x10^{-2}	1.09	1.01	1.17
BMI >20.58	MTHFR1298 wt	FV1299 wt							10	2	85	3	5.40 x10^{-2}	1.02 x10-01	2.40 x10^{-2}	0.86	0.67	1.11
BMI ≤20.58	MTHFR1298 hetero	FV1299 wt							9	2	86	3	4.60 x10^{-2}	8.18 x10^{-2}	1.67 x10^{-2}	0.85	0.64	1.12
BMI ≤20.58	MTHFR1298 wt	FV1299 wt							14	4	81	1	1.72 x10^{-3}	9.51 x10^{-4}	1.07 x10^{-4}	0.79	0.61	1.01
BMI >20.58	FXIIIVal34Leu wt								5	2	90	3	1.91 x10^{-2}	1.93 x10^{-2}	1.50 x10^{-3}	0.74	0.46	1.18
BMI ≤20.58	FXIIIVal34Leu wt								2	1	93	4	7.20 x10^{-2}	1.73 x10-01	1.11 x10^{-2}	0.7	0.31	1.55

(B) Risk ratio for NIHSS at admission >9.83±2.85

Factor1	Factor2	Factor3	Factor4	Factor5	Factor6	Factor7	Factor8	Factor9	Studied group (N)		Control group (N)		p_{fisher}	p_{yates}	p_{uncor}	RR	lower	upper
									NIHSS0h >9.83 ±2.85	NIHSS0h ≤9.83 ±2.85	NIHSS0h >9.83 ±2.85	NIHSS0h ≤9.83 ±2.85						
PTsecond >13.25seconds	PTratio ≤99	Creatinin >83.67umol/l	age >54y.o	height ≤161cm	FVLeiden wt	PAI14G5G hetero	FVCambridge wt		3	0	24	73	9.04 x10^{-3}	1.28 x10^{-2}	1.92 x10^{-3}	4.04	2.86	5.72

PTsecond ≤13.25seconds	PTratio ≤99	Creatinin >83.67umol/l	age >54y.o	height >161cm	FIIprothrombin wt	MTHFR677 wt	Notch3 hetero	nondiabetes	2	0	25	73	3.55 x10^{-2}	6.12 x10^{-2}	9.41 x10^{-3}	3.92	2.79	5.5
PTsecond ≤13.25seconds	PTratio >99	Creatinin >83.67umol/l	age >54y.o	height >161cm	MTHFR1298 wt	FV1299 wt			2	0	25	73	3.55 x10^{-2}	6.12 x10^{-2}	9.41 x10^{-3}	3.92	2.79	5.5
PTsecond ≤13.25seconds	PTratio ≤99	Creatinin >83.67umol/l	age >54y.o	height >161cm	MTHFR1298 hetero	FV1299 wt			2	0	25	73	3.55 x10^{-2}	6.12 x10^{-2}	9.41 x10^{-3}	3.92	2.79	5.5
PTsecond >13.25seconds	PTratio ≤99	Creatinin >83.67umol/l	age >54y.o	height ≤161cm	FIIprothrombin wt	MTHFR677 homozygous	Notch3 hetero	nondiabetes	1	0	26	73	1.35 x10-01	3.01 x10-01	4.92 x10^{-2}	3.81	2.74	5.3
PTsecond >13.25seconds	PTratio ≤99	Creatinin ≤83.67umol/l	age >54y.o	height ≤161cm	FIIprothrombin wt	MTHFR677 wt	Notch3 hetero	nondiabetes	1	0	26	73	1.35 x10-01	3.01 x10-01	4.92 x10^{-2}	3.81	2.74	5.3
PTsecond >13.25seconds	PTratio ≤99	Creatinin ≤83.67umol/l	age ≤54y.o	height >161cm	FIIprothrombin wt	MTHFR677 hetero	Notch3 hetero	nondiabetes	1	0	26	73	1.35 x10-01	3.01 x10-01	4.92 x10^{-2}	3.81	2.74	5.3
PTsecond >13.25seconds	PTratio ≤99	Creatinin ≤83.67umol/l	age ≤54y.o	height >161cm	FIIprothrombin wt	MTHFR677 wt	Notch3 wt	nondiabetes	1	0	26	73	1.35 x10-01	3.01 x10-01	4.92 x10^{-2}	3.81	2.74	5.3
PTsecond ≤13.25seconds	PTratio >99	Creatinin >83.67umol/l	age >54y.o	height >161cm	FIIprothrombin wt	MTHFR677 hetero	Notch3 hetero	diabetes	1	0	26	73	1.35 x10-01	3.01 x10-01	4.92 x10^{-2}	3.81	2.74	5.3
PTsecond ≤13.25seconds	PTratio >99	Creatinin >83.67umol/l	age >54y.o	height >161cm	FIIprothrombin wt	MTHFR677 wt	Notch3 hetero	nondiabetes	1	0	26	73	1.35 x10-01	3.01 x10-01	4.92 x10^{-2}	3.81	2.74	5.3
PTsecond ≤13.25seconds	PTratio >99	Creatinin >83.67umol/l	age >54y.o	height ≤161cm	FIIprothrombin wt	MTHFR677 hetero	Notch3 hetero	nondiabetes	1	0	26	73	1.35 x10-01	3.01 x10-01	4.92 x10^{-2}	3.81	2.74	5.3
PTsecond ≤13.25seconds	PTratio >99	Creatinin >83.67umol/l	age >54y.o	height ≤161cm	FIIprothrombin wt	MTHFR677 wt	Notch3 wt	diabetes	1	0	26	73	1.35 x10-01	3.01 x10-01	4.92 x10^{-2}	3.81	2.74	5.3
PTsecond ≤13.25seconds	PTratio >99	Creatinin >83.67umol/l	age ≤54y.o	height >161cm	FIIprothrombin wt	MTHFR677 wt	Notch3 wt	nondiabetes	1	0	26	73	1.35 x10-01	3.01 x10-01	4.92 x10^{-2}	3.81	2.74	5.3
PTsecond ≤13.25seconds	PTratio ≤99	Creatinin >83.67umol/l	age >54y.o	height >161cm	FIIprothrombin hetero	MTHFR677 hetero	Notch3 hetero	nondiabetes	1	0	26	73	1.35 x10-01	3.01 x10-01	4.92 x10^{-2}	3.81	2.74	5.3
PTsecond ≤13.25seconds	PTratio ≤99	Creatinin >83.67umol/l	age ≤54y.o	height >161cm	FIIprothrombin wt	MTHFR677 hetero	Notch3 homozygous	nondiabetes	1	0	26	73	1.35 x10-01	3.01 x10-01	4.92 x10^{-2}	3.81	2.74	5.3
PTsecond ≤13.25seconds	PTratio ≤99	Creatinin ≤83.67umol/l	age >54y.o	height >161cm	FIIprothrombin wt	MTHFR677 wt	Notch3 hetero	diabetes	1	0	26	73	1.35 x10-01	3.01 x10-01	4.92 x10^{-2}	3.81	2.74	5.3
PTsecond >13.25seconds	PTratio ≤99	Creatinin >83.67umol/l	age >54y.o	height ≤161cm	FVLeiden wt	PAI14G5G wt	FVCambridge wt		1	0	26	73	1.35 x10-01	3.01 x10-01	4.92 x10^{-2}	3.81	2.74	5.3

PTsecond >13.25seconds	PTratio ≤99	Creatinin >83.67umol/l	age ≤54y.o	height >161cm	FVLeiden wt	PAI14G5G homozygous	FVCambridge wt		1	0	26	73	1.35 x10-01	3.01 x10-01	4.92 $x10^{-2}$	3.81	2.74	5.3
PTsecond >13.25seconds	PTratio ≤99	Creatinin ≤83.67umol/l	age >54y.o	height >161cm	FVLeiden wt	PAI14G5G homozygous	FVCambridge wt		1	0	26	73	1.35 x10-01	3.01 x10-01	4.92 $x10^{-2}$	3.81	2.74	5.3
PTsecond >13.25seconds	PTratio ≤99	Creatinin ≤83.67umol/l	age >54y.o	height ≤161cm	FVLeiden wt	PAI14G5G homozygous	FVCambridge wt		1	0	26	73	1.35 x10-01	3.01 x10-01	4.92 $x10^{-2}$	3.81	2.74	5.3
PTsecond >13.25seconds	PTratio ≤99	Creatinin ≤83.67umol/l	age ≤54y.o	height >161cm	FVLeiden wt	PAI14G5G hetero	FVCambridge wt		1	0	26	73	1.35 x10-01	3.01 x10-01	4.92 $x10^{-2}$	3.81	2.74	5.3
PTsecond >13.25seconds	PTratio ≤99	Creatinin ≤83.67umol/l	age ≤54y.o	height >161cm	FVLeiden wt	PAI14G5G wt	FVCambridge wt		1	0	26	73	1.35 x10-01	3.01 x10-01	4.92 $x10^{-2}$	3.81	2.74	5.3
PTsecond ≤13.25seconds	PTratio >99	Creatinin ≤83.67umol/l	age >54y.o	height >161cm	FVLeiden wt	PAI14G5G wt	FVCambridge wt		1	0	26	73	1.35 x10-01	3.01 x10-01	4.92 $x10^{-2}$	3.81	2.74	5.3
PTsecond ≤13.25seconds	PTratio ≤99	Creatinin >83.67umol/l	age >54y.o	height >161cm	FVLeiden hetero	PAI14G5G homozygous	FVCambridge wt		1	0	26	73	1.35 x10-01	3.01 x10-01	4.92 $x10^{-2}$	3.81	2.74	5.3
PTsecond ≤13.25seconds	PTratio ≤99	Creatinin >83.67umol/l	age >54y.o	height >161cm	FVLeiden wt	PAI14G5G homozygous	FVCambridge wt		1	0	26	73	1.35 x10-01	3.01 x10-01	4.92 $x10^{-2}$	3.81	2.74	5.3
PTsecond ≤13.25seconds	PTratio ≤99	Creatinin >83.67umol/l	age ≤54y.o	height >161cm	FVLeiden wt	PAI14G5G hetero	FVCambridge wt		1	0	26	73	1.35 x10-01	3.01 x10-01	4.92 $x10^{-2}$	3.81	2.74	5.3
PTsecond ≤13.25seconds	PTratio ≤99	Creatinin ≤83.67umol/l	age >54y.o	height >161cm	FVLeiden wt	PAI14G5G wt	FVCambridge wt		1	0	26	73	1.35 x10-01	3.01 x10-01	4.92 $x10^{-2}$	3.81	2.74	5.3
PTsecond ≤13.25seconds	PTratio ≤99	Creatinin ≤83.67umol/l	age ≤54y.o	height >161cm	FVLeiden hetero	PAI14G5G wt	FVCambridge wt		1	0	26	73	1.35 x10-01	3.01 x10-01	4.92 $x10^{-2}$	3.81	2.74	5.3
PTsecond >13.25seconds	PTratio ≤99	Creatinin >83.67umol/l	age >54y.o	height >161cm	MTHFR1298 hetero	FV1299 hetero			1	0	26	73	1.35 x10-01	3.01 x10-01	4.92 $x10^{-2}$	3.81	2.74	5.3
PTsecond >13.25seconds	PTratio ≤99	Creatinin ≤83.67umol/l	age >54y.o	height ≤161cm	MTHFR1298 homozygous	FV1299 wt			1	0	26	73	1.35 x10-01	3.01 x10-01	4.92 $x10^{-2}$	3.81	2.74	5.3
PTsecond >13.25seconds	PTratio ≤99	Creatinin ≤83.67umol/l	age ≤54y.o	height >161cm	MTHFR1298 homozygous	FV1299 hetero			1	0	26	73	1.35 x10-01	3.01 x10-01	4.92 $x10^{-2}$	3.81	2.74	5.3

PTsecond >13.25seconds	PTratio ≤99	Creatinin ≤83.67umol/l	age ≤54y.o	height >161cm	MTHFR1298 wt	FV1299 wt			1	0	26	73	1.35 x10-01	3.01 x10-01	4.92 $x10^{-2}$	3.81	2.74	5.3
PTsecond ≤13.25seconds	PTratio ≤99	Creatinin >83.67umol/l	age ≤54y.o	height >161cm	MTHFR1298 wt	FV1299 wt			1	0	26	73	1.35 x10-01	3.01 x10-01	4.92 $x10^{-2}$	3.81	2.74	5.3
PTsecond ≤13.25seconds	PTratio ≤99	Creatinin ≤83.67umol/l	age ≤54y.o	height >161cm	MTHFR1298 wt	FV1299 wt			1	0	26	73	1.35 x10-01	3.01 x10-01	4.92 $x10^{-2}$	3.81	2.74	5.3
PTsecond ≤13.25seconds	PTratio ≤99	Creatinin >83.67umol/l	age ≤54y.o	height >161cm	FXIIIVal34Leu wt				1	0	26	73	1.35 x10-01	3.01 x10-01	4.92 $x10^{-2}$	3.81	2.74	5.3
PTsecond >13.25seconds	PTratio ≤99	Creatinin >83.67umol/l	age >54y.o	height ≤161cm	FXIIIVal34Leu wt				4	2	23	71	2.19 $x10^{-2}$	3.73 $x10^{-2}$	1.20 $x10^{-2}$	2.72	1.4	5.31
PTsecond >13.25seconds	PTratio ≤99	Creatinin >83.67umol/l	age >54y.o	height ≤161cm	FIIprothrombin wt	MTHFR677 wt	Notch3 hetero	nondiabetes	3	2	24	71	6.01 $x10^{-2}$	1.17 x10-01	4.41 $x10^{-2}$	2.38	1.07	5.26
PTsecond ≤13.25seconds	PTratio ≤99	Creatinin >83.67umol/l	age >54y.o	height >161cm	FXIIIVal34Leu wt				3	2	24	71	6.01 $x10^{-2}$	1.17 x10-01	4.41 $x10^{-2}$	2.38	1.07	5.26

(C) Risk ratio for NIHSS after 24h >7.92±2.04

Factor1	Factor2	Factor3	Factor4	Factor5	Factor6	Factor7	Factor8	Factor9	Studied group (N)		Control group (N)		p_{fisher}	p_{yates}	p_{uncor}	RR	lower	upper
									NIHSS24h >7.92±2.04	NIHSS24h ≤7.92±2.04	NIHSS24h >7.92±2.04	NIHSS24h ≤7.92±2.04						
PTsecond >13.25seconds	PTratio ≤99	Creatinin >83.67umol/l	age >54y.o	height ≤161cm	MTHFR1298 wt	FV1299 wt			3	0	31	66	1.85 $x10^{-2}$	3.35 $x10^{-2}$	7.14 $x10^{-3}$	3.13	2.34	4.18
PTsecond >13.25seconds	PTratio ≤99	Creatinin >83.67umol/l	age ≤54y.o	height >161cm	FIIprothrombin wt	MTHFR677 wt	Notch3 hetero	nondiabetes	2	0	32	66	5.67 $x10^{-2}$	1.08 x10-01	2.33 $x10^{-2}$	3.06	2.3	4.07
PTsecond ≤13.25seconds	PTratio >99	Creatinin >83.67umol/l	age >54y.o	height >161cm	MTHFR1298 wt	FV1299 wt			2	0	32	66	5.67 $x10^{-2}$	1.08 x10-01	2.33 $x10^{-2}$	3.06	2.3	4.07
PTsecond ≤13.25seconds	PTratio ≤99	Creatinin >83.67umol/l	age >54y.o	height >161cm	MTHFR1298 hetero	FV1299 wt			2	0	32	66	5.67 $x10^{-2}$	1.08 x10-01	2.33 $x10^{-2}$	3.06	2.3	4.07
PTsecond >13.25seconds	PTratio ≤99	Creatinin >83.67umol/l	age >54y.o	height ≤161cm	FXIIIVal34Leu wt				4	2	30	64	8.81 $x10^{-2}$	9.72 $x10^{-2}$	4.07 $x10^{-2}$	2.09	1.1	3.95

(D) Risk ratio for NIHSS at discharges >6.85±2.90

Factor1	Factor2	Factor3	Factor4	Factor5	Factor6	Factor7	Factor8	Factor9	Studied group		Control group		p_{fisher}	p_{yates}	p_{uncor}	RR	lower	upper
									NIHSSend >6.85 ±2.90	NIHSSend ≤6.85 ±2.90	NIHSSend >6.85±2.90	NIHSSend ≤6.85 ±2.90						
PTsecond >13.25seconds	PTratio ≤99	Creatinin ≤83.67umol/l	age >54y.o	height ≤161cm	FIIprothrombin wt	MTHFR677 hetero	Notch3 hetero	nondiabetes	1	0	11	88	6.00 $x10^{-2}$	1.20 x10-01	3.25 $x10^{-3}$	9	5.16	15.71
PTsecond ≤13.25seconds	PTratio >99	Creatinin >83.67umol/l	age >54y.o	height >161cm	FIIprothrombin wt	MTHFR677 hetero	Notch3 hetero	diabetes	1	0	11	88	6.00 $x10^{-2}$	1.20 x10-01	3.25 $x10^{-3}$	9	5.16	15.71
PTsecond ≤13.25seconds	PTratio >99	Creatinin >83.67umol/l	age >54y.o	height >161cm	FIIprothrombin wt	MTHFR677 wt	Notch3 hetero	nondiabetes	1	0	11	88	6.00 $x10^{-2}$	1.20 x10-01	3.25 $x10^{-3}$	9	5.16	15.71
PTsecond ≤13.25seconds	PTratio >99	Creatinin >83.67umol/l	age >54y.o	height ≤161cm	FIIprothrombin wt	MTHFR677 hetero	Notch3 hetero	nondiabetes	1	0	11	88	6.00 $x10^{-2}$	1.20 x10-01	3.25 $x10^{-3}$	9	5.16	15.71
PTsecond ≤13.25seconds	PTratio >99	Creatinin >83.67umol/l	age >54y.o	height ≤161cm	FIIprothrombin wt	MTHFR677 wt	Notch3 wt	diabetes	1	0	11	88	6.00 $x10^{-2}$	1.20 x10-01	3.25 $x10^{-3}$	9	5.16	15.71
PTsecond ≤13.25seconds	PTratio >99	Creatinin >83.67umol/l	age ≤54y.o	height >161cm	FIIprothrombin wt	MTHFR677 wt	Notch3 wt	nondiabetes	1	0	11	88	6.00 $x10^{-2}$	1.20 x10-01	3.25 $x10^{-3}$	9	5.16	15.71
PTsecond ≤13.25seconds	PTratio ≤99	Creatinin >83.67umol/l	age >54y.o	height >161cm	FIIprothrombin hetero	MTHFR677 hetero	Notch3 hetero	nondiabetes	1	0	11	88	6.00 $x10^{-2}$	1.20 x10-01	3.25 $x10^{-3}$	9	5.16	15.71
PTsecond >13.25seconds	PTratio ≤99	Creatinin >83.67umol/l	age >54y.o	height >161cm	FIIprothrombin wt	MTHFR677 hetero	Notch3 hetero	nondiabetes	2	2	10	86	3.47 $x10^{-2}$	5.46 $x10^{-2}$	8.49 $x10^{-3}$	4.8	1.53	15.04
PTsecond >13.25seconds	PTratio ≤99	Creatinin >83.67umol/l	age >54y.o	height ≤161cm	FIIprothrombin wt	MTHFR677 wt	Notch3 hetero	nondiabetes	2	3	10	85	5.40 $x10^{-2}$	1.02 x10-01	2.40 $x10^{-2}$	3.8	1.12	12.91

(E) Risk ratio for the modified Rankinscale (mRS) >2.86±1.21

Factor1	Factor2	Factor3	Factor4	Factor5	Factor6	Factor7	Factor8	Factor9	Studied group		Control group		p_{fisher}	p_{yates}	p_{uncor}	RR	lower	upper
									Rankin >2.86±1.21	Rankin ≤2.86 ± 1.21	Rankin >2.86±1.21	Rankin ≤2.86 ± 1.21						

INR ≤1.02	PTsecond >13.25seconds	PTratio ≤99	Creatinin ≤83.67umol/l	NinfarCT ≤2	FIIprothrombin wt	MTHFR677 wt	Notch3 hetero	nondiabetes	3	0	23	74	8.04 x10^{-3}	1.08 x10^{-2}	1.50 x10^{-3}	4.22	2.95	6.03
INR >1.02	PTsecond >13.25seconds	PTratio ≤99	Creatinin >83.67umol/l	Ninfarcts CT >2	FVLeiden wt	PAI14G5G hetero	FVCambridge wt		3	0	23	74	8.04 x10^{-3}	1.08 x10^{-2}	1.50 x10^{-3}	4.22	2.95	6.03
INR ≤1.02	PTsecond >13.25seconds	PTratio ≤99	Creatinin ≤83.67umol/l	NinfarCT ≤2	FIIprothrombin wt	MTHFR677 wt	Notch3 hetero	nondiabetes	2	0	24	74	3.28 x10^{-2}	5.53 x10^{-2}	7.97 x10^{-3}	4.08	2.88	5.78
INR ≤1.02	PTsecond >13.25seconds	PTratio ≤99	Creatinin >83.67umol/l	Ninfarcts CT >2	FVLeiden wt	PAI14G5G homozygous	FVCambridge wt		2	0	24	74	3.28 x10^{-2}	5.53 x10^{-2}	7.97 x10^{-3}	4.08	2.88	5.78
INR >1.02	PTsecond >13.25seconds	PTratio ≤99	Creatinin >83.67umol/l	Ninfarcts CT >2	MTHFR1298 wt	FV1299 wt			2	0	24	74	3.28 x10^{-2}	5.53 x10^{-2}	7.97 x10^{-3}	4.08	2.88	5.78
INR ≤1.02	PTsecond ≤13.25seconds	PTratio ≤99	Creatinin >83.67umol/l	Ninfarcts CT ≤2	MTHFR1298 hetero	FV1299 wt			2	0	24	74	3.28 x10^{-2}	5.53 x10^{-2}	7.97 x10^{-3}	4.08	2.88	5.78
INR >1.02	PTsecond >13.25seconds	PTratio ≤99	Creatinin ≤83.67umol/l	Ninfarcts CT >2	FXIIIVal34Leu wt				1	1	25	73	3.28 x10^{-2}	5.53 x10^{-2}	7.97 x10^{-3}	4.08	2.88	5.78
INR ≤1.02	PTsecond >13.25seconds	PTratio ≤99	Creatinin ≤83.67umol/l	NinfarCT ≤2	FIIprothrombin wt	MTHFR677 hetero	Notch3 hetero	nondiabetes	1	0	25	74	1.30 x10-01	2.91 x10-01	4.50 x10^{-2}	3.96	2.82	5.56
INR ≤1.02	PTsecond >13.25seconds	PTratio ≤99	Creatinin ≤83.67umol/l	NinfarCT ≤2	FIIprothrombin wt	MTHFR677 homozygous	Notch3 hetero	nondiabetes	1	0	25	74	1.30 x10-01	2.91 x10-01	4.50 x10^{-2}	3.96	2.82	5.56
INR ≤1.02	PTsecond >13.25seconds	PTratio ≤99	Creatinin ≤83.67umol/l	NinfarCT ≤2	FIIprothrombin wt	MTHFR677 hetero	Notch3 hetero	diabetes	1	0	25	74	1.30 x10-01	2.91 x10-01	4.50 x10^{-2}	3.96	2.82	5.56
INR ≤1.02	PTsecond ≤13.25seconds	PTratio ≤99	Creatinin ≤83.67umol/l	NinfarCT ≤2	FIIprothrombin wt	MTHFR677 hetero	Notch3 hetero	nondiabetes	1	0	25	74	1.30 x10-01	2.91 x10-01	4.50 x10^{-2}	3.96	2.82	5.56
INR ≤1.02	PTsecond ≤13.25seconds	PTratio ≤99	Creatinin ≤83.67umol/l	NinfarCT ≤2	FIIprothrombin wt	MTHFR677 wt	Notch3 wt	nondiabetes	1	0	25	74	1.30 x10-01	2.91 x10-01	4.50 x10^{-2}	3.96	2.82	5.56
INR ≤1.02	PTsecond ≤13.25seconds	PTratio ≤99	Creatinin ≤83.67umol/l	NinfarCT ≤2	FIIprothrombin wt	MTHFR677 wt	Notch3 wt	diabetes	1	0	25	74	1.30 x10-01	2.91 x10-01	4.50 x10^{-2}	3.96	2.82	5.56
INR ≤1.02	PTsecond ≤13.25seconds	PTratio ≤99	Creatinin ≤83.67umol/l	NinfarCT ≤2	FIIprothrombin wt	MTHFR677 wt	Notch3 hetero	nondiabetes	1	0	25	74	1.30 x10-01	2.91 x10-01	4.50 x10^{-2}	3.96	2.82	5.56
INR ≤1.02	PTsecond ≤13.25seconds	PTratio ≤99	Creatinin ≤83.67umol/l	NinfarCT ≤2	FIIprothrombin hetero	MTHFR677 hetero	Notch3 hetero	nondiabetes	1	0	25	74	1.30 x10-01	2.91 x10-01	4.50 x10^{-2}	3.96	2.82	5.56
INR ≤1.02	PTsecond ≤13.25seconds	PTratio ≤99	Creatinin ≤83.67umol/l	NinfarCT ≤2	FIIprothrombin wt	MTHFR677 homozygous	Notch3 hetero	nondiabetes	1	0	25	74	1.30 x10-01	2.91 x10-01	4.50 x10^{-2}	3.96	2.82	5.56

INR >1.02	PTsecond >13.25seconds	PTratio ≤99	Creatinin >83.67umol/l	Ninfarcts CT >2	FVLeiden wt	PAI14G5G homozygous	FVCambridge wt		1	0	25	74	1.30 x10-01	2.91 x10-01	4.50 x10^{-2}	3.96	2.82	5.56
INR >1.02	PTsecond >13.25seconds	PTratio ≤99	Creatinin >83.67umol/l	Ninfarcts CT ≤2	FVLeiden hetero	PAI14G5G homozygous	FVCambridge wt		1	0	25	74	1.30 x10-01	2.91 x10-01	4.50 x10^{-2}	3.96	2.82	5.56
INR >1.02	PTsecond >13.25seconds	PTratio ≤99	Creatinin >83.67umol/l	Ninfarcts CT ≤2	FVLeiden wt	PAI14G5G wt	FVCambridge wt		1	0	25	74	1.30 x10-01	2.91 x10-01	4.50 x10^{-2}	3.96	2.82	5.56
INR >1.02	PTsecond >13.25seconds	PTratio ≤99	Creatinin ≤83.67umol/l	Ninfarcts CT >2	FVLeiden wt	PAI14G5G homozygous	FVCambridge wt		1	0	25	74	1.30 x10-01	2.91 x10-01	4.50 x10^{-2}	3.96	2.82	5.56
INR >1.02	PTsecond >13.25seconds	PTratio ≤99	Creatinin ≤83.67umol/l	Ninfarcts CT ≤2	FVLeiden hetero	PAI14G5G hetero	FVCambridge wt		1	0	25	74	1.30 x10-01	2.91 x10-01	4.50 x10^{-2}	3.96	2.82	5.56
INR ≤1.02	PTsecond ≤13.25seconds	PTratio >99	Creatinin ≤83.67umol/l	Ninfarcts CT >2	FVLeiden wt	PAI14G5G wt	FVCambridge wt		1	0	25	74	1.30 x10-01	2.91 x10-01	4.50 x10^{-2}	3.96	2.82	5.56
INR ≤1.02	PTsecond ≤13.25seconds	PTratio ≤99	Creatinin >83.67umol/l	Ninfarcts CT ≤2	FVLeiden hetero	PAI14G5G homozygous	FVCambridge wt		1	0	25	74	1.30 x10-01	2.91 x10-01	4.50 x10^{-2}	3.96	2.82	5.56
INR ≤1.02	PTsecond ≤13.25seconds	PTratio ≤99	Creatinin >83.67umol/l	Ninfarcts CT ≤2	FVLeiden wt	PAI14G5G homozygous	FVCambridge wt		1	0	25	74	1.30 x10-01	2.91 x10-01	4.50 x10^{-2}	3.96	2.82	5.56
INR ≤1.02	PTsecond ≤13.25seconds	PTratio ≤99	Creatinin >83.67umol/l	Ninfarcts CT ≤2	FVLeiden wt	PAI14G5G wt	FVCambridge wt		1	0	25	74	1.30 x10-01	2.91 x10-01	4.50 x10^{-2}	3.96	2.82	5.56
INR ≤1.02	PTsecond ≤13.25seconds	PTratio ≤99	Creatinin ≤83.67umol/l	Ninfarcts CT >2	FVLeiden hetero	PAI14G5G wt	FVCambridge wt		1	0	25	74	1.30 x10-01	2.91 x10-01	4.50 x10^{-2}	3.96	2.82	5.56
INR >1.02	PTsecond >13.25seconds	PTratio ≤99	Creatinin >83.67umol/l	Ninfarcts CT >2	MTHFR1298 hetero	FV1299 wt			1	0	25	74	1.30 x10-01	2.91 x10-01	4.50 x10^{-2}	3.96	2.82	5.56
INR >1.02	PTsecond >13.25seconds	PTratio ≤99	Creatinin >83.67umol/l	Ninfarcts CT >2	MTHFR1298 homozygous	FV1299 wt			1	0	25	74	1.30 x10-01	2.91 x10-01	4.50 x10^{-2}	3.96	2.82	5.56
INR >1.02	PTsecond >13.25seconds	PTratio ≤99	Creatinin ≤83.67umol/l	Ninfarcts CT >2	MTHFR1298 homozygous	FV1299 wt			1	0	25	74	1.30 x10-01	2.91 x10-01	4.50 x10^{-2}	3.96	2.82	5.56
INR ≤1.02	PTsecond >13.25seconds	PTratio ≤99	Creatinin >83.67umol/l	Ninfarcts CT >2	MTHFR1298 hetero	FV1299 hetero			1	0	25	74	1.30 x10-01	2.91 x10-01	4.50 x10^{-2}	3.96	2.82	5.56
INR ≤1.02	PTsecond >13.25seconds	PTratio ≤99	Creatinin >83.67umol/l	Ninfarcts CT >2	MTHFR1298 wt	FV1299 wt			1	0	25	74	1.30 x10-01	2.91 x10-01	4.50 x10^{-2}	3.96	2.82	5.56
INR ≤1.02	PTsecond ≤13.25seconds	PTratio >99	Creatinin ≤83.67umol/l	Ninfarcts CT >2	MTHFR1298 wt	FV1299 wt			1	0	25	74	1.30 x10-01	2.91 x10-01	4.50 x10^{-2}	3.96	2.82	5.56

INR >1.02	PTsecond >13.25seconds	PTratio ≤99	Creatinin >83.67umol/l	Ninfarcts CT >2	FXIIIVal34Leu wt				4	1	22	73	1.30 x10-01	2.91 x10-01	4.50 $x10^{-2}$	3.96	2.82	5.56
INR >1.02	PTsecond >13.25seconds	PTratio ≤99	Creatinin >83.67umol/l	Ninfarcts CT ≤2	MTHFR1298 hetero	FV1299 wt			3	1	23	73	2.64 $x10^{-2}$	4.47 $x10^{-2}$	1.13 $x10^{-2}$	3.13	1.6	6.11
INR >1.02	PTsecond >13.25seconds	PTratio ≤99	Creatinin >83.67umol/l	Ninfarcts CT ≤2	MTHFR1298 wt	FV1299 wt			3	1	23	73	2.64 $x10^{-2}$	4.47 $x10^{-2}$	1.13 $x10^{-2}$	3.13	1.6	6.11
INR >1.02	PTsecond >13.25seconds	PTratio ≤99	Creatinin ≤83.67umol/l	Ninfarcts CT ≤2	FXIIIVal34Leu wt				2	10	24	64	2.64 $x10^{-2}$	4.47 $x10^{-2}$	1.13 $x10^{-2}$	3.13	1.6	6.11
INR ≤1.02	PTsecond >13.25seconds	PTratio ≤99	Creatinin ≤83.67umol/l	NinfarCT ≤2	FIIprothrombin wt	MTHFR677 wt	Notch3 hetero	nondiabetes	3	2	23	72	5.44 $x10^{-2}$	1.05 x10-01	3.77 $x10^{-2}$	2.48	1.11	5.51
