# Supplementary material for: The Roles of NOTCH3 p.R544C and Thrombophilia Genes in Vietnamese Patients With Ischemic Stroke: Study Involving a Hierarchical Cluster Analysis
Source: JMIR Bioinform Biotechnol. 2024 May 7;5:e56884. doi: 10.2196/56884 (PMC11135231; doi:10.2196/56884)

Multimedia Appendix 2. Forest plots.

Figure S1. Forest plot of the risk of a modified Rankin scale score >2.86 in cluster 4 (FII Prothrombin genotype, MTHFR-C677T genotype, NOTCH3 p.R544C genotype, and diabetes status) with international normalized ratio (INR), prothrombin (PT) time and ratio, creatinine, and number of infarcts on computed tomography (CT).

Study	Rankin>2.86 Events	Rankin>2.86 Total	Rankin≤2.86 Events	Rankin≤2.86 Total	Weight	Risk Ratio MH, Fixed, 95% CI
INR>1.02_PT_second_>13.25seconds_PT_ratio≤99_Creatinin≤83.67umol/l_N_infarctsCT≤2_FII_prothrombin_wt_MTHFR_677_hetero_Notch_3_hetero_non diabetes	1	4	25	96	7.8%	0.96 [0.17; 5.42]
INR≤1.02_PT_second_≤13.25seconds_PT_ratio>99_Creatinin>83.67umol/l_N_infarctsCT>2_FII_prothrombin_wt_MTHFR_677_wt_Notch_3_hetero_non diabetes	1	4	25	96	7.8%	0.96 [0.17; 5.42]
INR>1.02_PT_second_>13.25seconds_PT_ratio≤99_Creatinin>83.67umol/l_N_infarctsCT>2_FII_prothrombin_wt_MTHFR_677_hetero_Notch_3_hetero_non diabetes	1	2	25	98	3.9%	1.96 [0.47; 8.16]
INR>1.02_PT_second_>13.25seconds_PT_ratio≤99_Creatinin>83.67umol/l_N_infarctsCT≤2_FII_prothrombin_wt_MTHFR_677_wt_Notch_3_hetero_diabetes	1	2	25	98	3.9%	1.96 [0.47; 8.16]
INR>1.02_PT_second_>13.25seconds_PT_ratio≤99_Creatinin≤83.67umol/l_N_infarctsCT>2_FII_prothrombin_wt_MTHFR_677_wt_Notch_3_hetero_non diabetes	1	2	25	98	3.9%	1.96 [0.47; 8.16]
INR≤1.02_PT_second_≤13.25seconds_PT_ratio>99_Creatinin>83.67umol/l_N_infarctsCT≤2_FII_prothrombin_wt_MTHFR_677_hetero_Notch_3_hetero_diabetes	1	2	25	98	3.9%	1.96 [0.47; 8.16]
INR≤1.02_PT_second_≤13.25seconds_PT_ratio>99_Creatinin>83.67umol/l_N_infarctsCT≤2_FII_prothrombin_wt_MTHFR_677_hetero_Notch_3_hetero_non diabetes	1	2	25	98	3.9%	1.96 [0.47; 8.16]
INR≤1.02_PT_second_≤13.25seconds_PT_ratio≤99_Creatinin>83.67umol/l_N_infarctsCT≤2_FII_prothrombin_wt_MTHFR_677_wt_Notch_3_hetero_non diabetes	1	2	25	98	3.9%	1.96 [0.47; 8.16]
INR≤1.02_PT_second_≤13.25seconds_PT_ratio≤99_Creatinin≤83.67umol/l_N_infarctsCT>2_FII_prothrombin_wt_MTHFR_677_hetero_Notch_3_hetero_non diabetes	1	2	25	98	3.9%	1.96 [0.47; 8.16]
INR>1.02_PT_second_>13.25seconds_PT_ratio≤99_Creatinin>83.67umol/l_N_infarctsCT≤2_FII_prothrombin_wt_MTHFR_677_wt_Notch_3_hetero_non diabetes	3	5	23	95	9.0%	2.48 [1.11; 5.51]
INR>1.02_PT_second_>13.25seconds_PT_ratio≤99_Creatinin>83.67umol/l_N_infarctsCT≤2_FII_prothrombin_wt_MTHFR_677_hetero_Notch_3_hetero_non diabetes	1	1	25	99	3.9%	3.90 [2.79; 5.45]
INR>1.02_PT_second_>13.25seconds_PT_ratio≤99_Creatinin>83.67umol/l_N_infarctsCT≤2_FII_prothrombin_wt_MTHFR_677_homo_Notch_3_hetero_non diabetes	1	1	25	99	3.9%	3.90 [2.79; 5.45]
INR>1.02_PT_second_>13.25seconds_PT_ratio≤99_Creatinin≤83.67umol/l_N_infarctsCT≤2_FII_prothrombin_wt_MTHFR_677_hetero_Notch_3_hetero_diabetes	1	1	25	99	3.9%	3.90 [2.79; 5.45]
INR>1.02_PT_second_≤13.25seconds_PT_ratio≤99_Creatinin≤83.67umol/l_N_infarctsCT≤2_FII_prothrombin_wt_MTHFR_677_hetero_Notch_3_hetero_non diabetes	1	1	25	99	3.9%	3.90 [2.79; 5.45]
INR≤1.02_PT_second_≤13.25seconds_PT_ratio>99_Creatinin>83.67umol/l_N_infarctsCT>2_FII_prothrombin_wt_MTHFR_677_wt_Notch_3_wt_non diabetes	1	1	25	99	3.9%	3.90 [2.79; 5.45]
INR≤1.02_PT_second_≤13.25seconds_PT_ratio>99_Creatinin>83.67umol/l_N_infarctsCT≤2_FII_prothrombin_wt_MTHFR_677_wt_Notch_3_wt_diabetes	1	1	25	99	3.9%	3.90 [2.79; 5.45]
INR≤1.02_PT_second_≤13.25seconds_PT_ratio>99_Creatinin≤83.67umol/l_N_infarctsCT>2_FII_prothrombin_wt_MTHFR_677_wt_Notch_3_hetero_non diabetes	1	1	25	99	3.9%	3.90 [2.79; 5.45]
INR≤1.02_PT_second_≤13.25seconds_PT_ratio≤99_Creatinin>83.67umol/l_N_infarctsCT≤2_FII_prothrombin_hetero_MTHFR_677_hetero_Notch_3_hetero_non diabetes	1	1	25	99	3.9%	3.90 [2.79; 5.45]
INR≤1.02_PT_second_≤13.25seconds_PT_ratio≤99_Creatinin>83.67umol/l_N_infarctsCT≤2_FII_prothrombin_wt_MTHFR_677_homo_Notch_3_hetero_non diabetes	1	1	25	99	3.9%	3.90 [2.79; 5.45]
INR≤1.02_PT_second_>13.25seconds_PT_ratio≤99_Creatinin>83.67umol/l_N_infarctsCT>2_FII_prothrombin_wt_MTHFR_677_wt_Notch_3_hetero_non diabetes	2	2	24	98	5.6%	4.02 [2.85; 5.67]
INR>1.02_PT_second_>13.25seconds_PT_ratio≤99_Creatinin>83.67umol/l_N_infarctsCT>2_FII_prothrombin_wt_MTHFR_677_wt_Notch_3_hetero_non diabetes	3	3	23	97	7.2%	4.15 [2.92; 5.90]
**Total (95% CI)**		**41**		**2059**	**100.0%**	**2.40 [1.90; 3.02]**
**Prediction interval**						**[3.40; 4.20]**

Heterogeneity: $Tau^2 = 0$; $Chi^2 = 12.29$, df = 20 ($P = 0.91$); $I^2 = 0\%$

Test for overall effect: $Z = 7.36$ ($P < 0.01$)

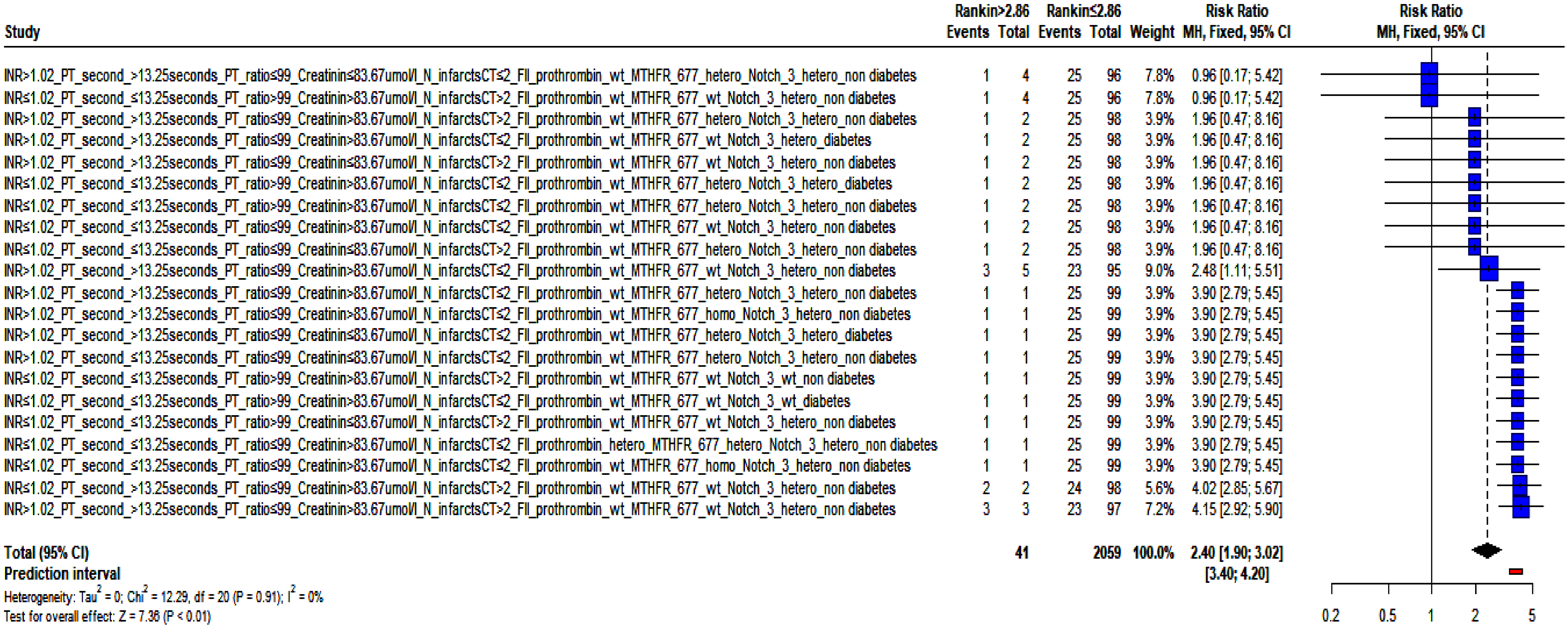

Figure S2. Forest plot of the risk of a National Institutes of Health Stroke Scale (NIHSS) score at admission >9.83 in cluster 4 (FII Prothrombin genotype, MTHFR-C677T genotype, NOTCH3 p.R544C genotype, and diabetes status) with prothrombin (PT) time and ratio, creatinine, patient age, and patient height.

Study	NIHSS0h>9.83 Events	NIHSS0h>9.83 Total	NIHSS0h≤9.83 Events	NIHSS0h≤9.83 Total	Weight	Risk Ratio MH, Fixed, 95% CI
PT_second_≤13.25seconds_PT_ratio>99_Creatinin>83.67umol/l_age>54y.o_height≤161cm_FII_prothrombin_wt_MTHFR_677_wt_Notch_3_hetero_non diabetes	0	5	27	95	10.0%	0.32 [0.02; 4.52]
PT_second_≤13.25seconds_PT_ratio>99_Creatinin≤83.67umol/l_age≤54y.o_height≤161cm_FII_prothrombin_wt_MTHFR_677_hetero_Notch_3_hetero_non diabetes	0	3	27	97	6.7%	0.51 [0.04; 6.72]
PT_second_>13.25seconds_PT_ratio≤99_Creatinin≤83.67umol/l_age≤54y.o_height≤161cm_FII_prothrombin_wt_MTHFR_677_hetero_Notch_3_hetero_non diabetes	0	2	27	98	5.0%	0.72 [0.06; 8.72]
PT_second_>13.25seconds_PT_ratio>99_Creatinin>83.67umol/l_age≤54y.o_height>161cm_FII_prothrombin_wt_MTHFR_677_hetero_Notch_3_hetero_diabetes	0	1	27	99	3.3%	1.21 [0.12; 11.86]
PT_second_>13.25seconds_PT_ratio>99_Creatinin≤83.67umol/l_age>54y.o_height≤161cm_FII_prothrombin_wt_MTHFR_677_wt_Notch_3_hetero_non diabetes	0	1	27	99	3.3%	1.21 [0.12; 11.86]
PT_second_>13.25seconds_PT_ratio>99_Creatinin≤83.67umol/l_age≤54y.o_height>161cm_FII_prothrombin_wt_MTHFR_677_hetero_Notch_3_hetero_non diabetes	0	1	27	99	3.3%	1.21 [0.12; 11.86]
PT_second_>13.25seconds_PT_ratio>99_Creatinin≤83.67umol/l_age≤54y.o_height≤161cm_FII_prothrombin_wt_MTHFR_677_hetero_Notch_3_hetero_non diabetes	0	1	27	99	3.3%	1.21 [0.12; 11.86]
PT_second_>13.25seconds_PT_ratio>99_Creatinin≤83.67umol/l_age≤54y.o_height≤161cm_FII_prothrombin_wt_MTHFR_677_wt_Notch_3_hetero_non diabetes	0	1	27	99	3.3%	1.21 [0.12; 11.86]
PT_second_>13.25seconds_PT_ratio≤99_Creatinin>83.67umol/l_age>54y.o_height>161cm_FII_prothrombin_wt_MTHFR_677_wt_Notch_3_hetero_diabetes	0	1	27	99	3.3%	1.21 [0.12; 11.86]
PT_second_>13.25seconds_PT_ratio≤99_Creatinin≤83.67umol/l_age>54y.o_height≤161cm_FII_prothrombin_wt_MTHFR_677_hetero_Notch_3_hetero_diabetes	0	1	27	99	3.3%	1.21 [0.12; 11.86]
PT_second_≤13.25seconds_PT_ratio>99_Creatinin>83.67umol/l_age>54y.o_height≤161cm_FII_prothrombin_wt_MTHFR_677_wt_Notch_3_hetero_diabetes	0	1	27	99	3.3%	1.21 [0.12; 11.86]
PT_second_≤13.25seconds_PT_ratio>99_Creatinin>83.67umol/l_age≤54y.o_height≤161cm_FII_prothrombin_wt_MTHFR_677_wt_Notch_3_hetero_non diabetes	0	1	27	99	3.3%	1.21 [0.12; 11.86]
PT_second_≤13.25seconds_PT_ratio>99_Creatinin≤83.67umol/l_age≤54y.o_height>161cm_FII_prothrombin_homo_MTHFR_677_hetero_Notch_3_hetero_diabetes	0	1	27	99	3.3%	1.21 [0.12; 11.86]
PT_second_≤13.25seconds_PT_ratio>99_Creatinin≤83.67umol/l_age≤54y.o_height≤161cm_FII_prothrombin_wt_MTHFR_677_wt_Notch_3_hetero_non diabetes	0	1	27	99	3.3%	1.21 [0.12; 11.86]
PT_second_>13.25seconds_PT_ratio≤99_Creatinin>83.67umol/l_age>54y.o_height>161cm_FII_prothrombin_wt_MTHFR_677_wt_Notch_3_hetero_non diabetes	1	3	26	97	4.8%	1.24 [0.24; 6.37]
PT_second_≤13.25seconds_PT_ratio≤99_Creatinin≤83.67umol/l_age≤54y.o_height>161cm_FII_prothrombin_wt_MTHFR_677_hetero_Notch_3_hetero_non diabetes	1	2	26	98	3.2%	1.88 [0.45; 7.83]
PT_second_>13.25seconds_PT_ratio≤99_Creatinin>83.67umol/l_age>54y.o_height>161cm_FII_prothrombin_wt_MTHFR_677_hetero_Notch_3_hetero_non diabetes	2	4	25	96	6.2%	1.92 [0.68; 5.41]
PT_second_>13.25seconds_PT_ratio≤99_Creatinin>83.67umol/l_age>54y.o_height≤161cm_FII_prothrombin_wt_MTHFR_677_homo_Notch_3_hetero_non diabetes	1	1	26	99	3.2%	3.75 [2.71; 5.20]
PT_second_≤13.25seconds_PT_ratio>99_Creatinin>83.67umol/l_age>54y.o_height≤161cm_FII_prothrombin_wt_MTHFR_677_hetero_Notch_3_hetero_non diabetes	1	1	26	99	3.2%	3.75 [2.71; 5.20]
PT_second_≤13.25seconds_PT_ratio>99_Creatinin>83.67umol/l_age>54y.o_height≤161cm_FII_prothrombin_wt_MTHFR_677_wt_Notch_3_wt_diabetes	1	1	26	99	3.2%	3.75 [2.71; 5.20]
PT_second_≤13.25seconds_PT_ratio>99_Creatinin>83.67umol/l_age≤54y.o_height>161cm_FII_prothrombin_wt_MTHFR_677_wt_Notch_3_wt_non diabetes	1	1	26	99	3.2%	3.75 [2.71; 5.20]
PT_second_≤13.25seconds_PT_ratio≤99_Creatinin>83.67umol/l_age>54y.o_height>161cm_FII_prothrombin_hetero_MTHFR_677_hetero_Notch_3_hetero_non diabetes	1	1	26	99	3.2%	3.75 [2.71; 5.20]
PT_second_≤13.25seconds_PT_ratio≤99_Creatinin>83.67umol/l_age≤54y.o_height>161cm_FII_prothrombin_wt_MTHFR_677_hetero_Notch_3_homo_non diabetes	1	1	26	99	3.2%	3.75 [2.71; 5.20]
PT_second_≤13.25seconds_PT_ratio≤99_Creatinin≤83.67umol/l_age>54y.o_height>161cm_FII_prothrombin_wt_MTHFR_677_wt_Notch_3_hetero_diabetes	1	1	26	99	3.2%	3.75 [2.71; 5.20]
PT_second_≤13.25seconds_PT_ratio≤99_Creatinin>83.67umol/l_age>54y.o_height>161cm_FII_prothrombin_wt_MTHFR_677_wt_Notch_3_hetero_non diabetes	2	2	25	98	4.7%	3.86 [2.77; 5.40]
**Total (95% CI)**		**39**		**2461**	**100.0%**	**1.45 [1.06; 2.00]**
**Prediction interval**						**[3.15; 4.00]**

Heterogeneity: $Tau^2 = 0$; $Chi^2 = 21.09$, df = 24 ($P = 0.63$); $I^2 = 0\%$

Test for overall effect: $Z = 2.30$ ($P = 0.02$)

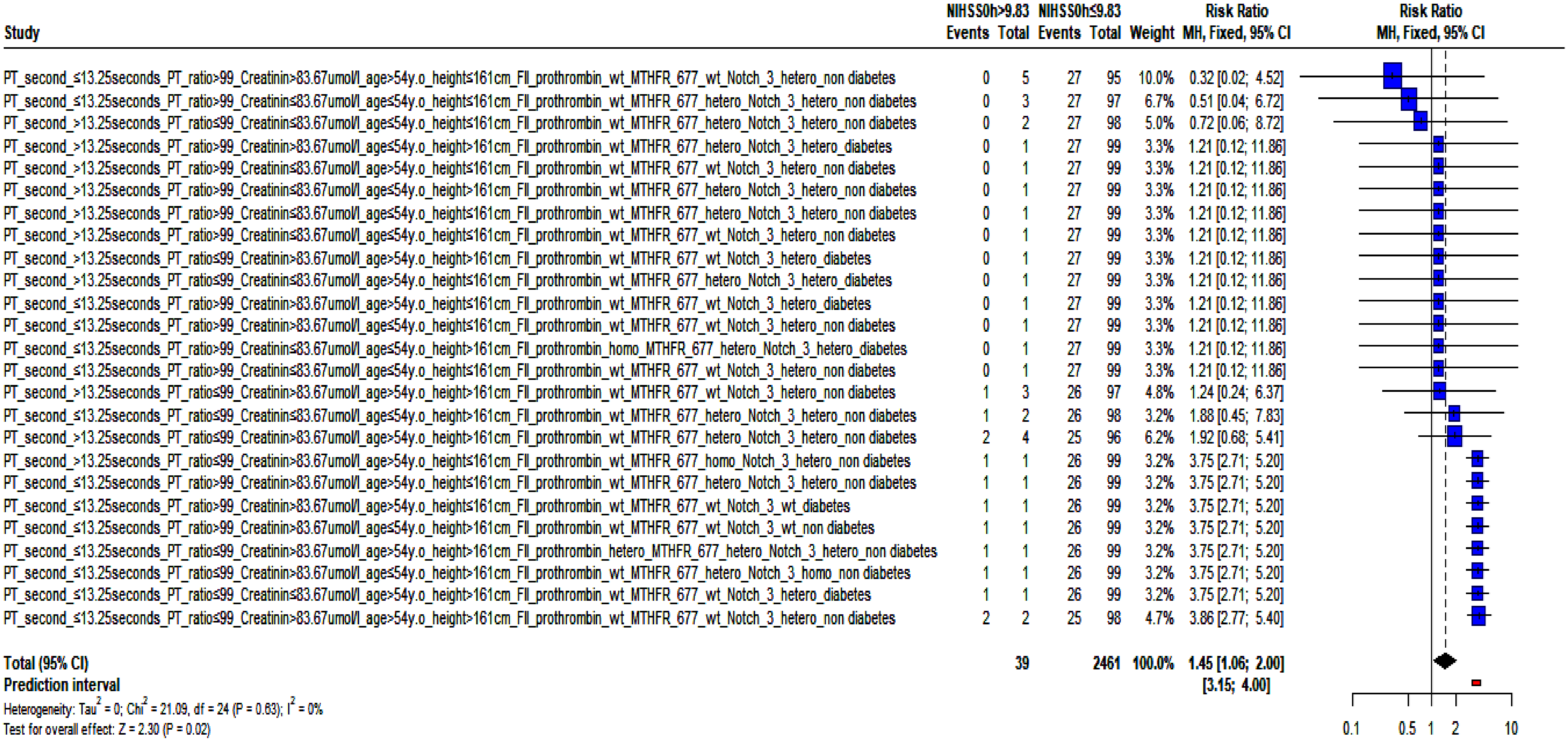

Figure S3. Forest plot of the risk of a National Institutes of Health Stroke Scale (NIHSS) score after 24 hours >7.92 in cluster 4 (FII Prothrombin genotype, MTHFR-C677T genotype, NOTCH3 p.R544C genotype, and diabetes status) with prothrombin (PT) time and ratio, creatinine, patient age, and patient height.

Study	NIHSS24h>7.92 Events	NIHSS24h>7.92 Total	NIHSS24h≤7.92 Events	NIHSS24h≤7.92 Total	Weight	Risk Ratio MH, Fixed, 95% CI
PT_second_≤13.25seconds_PT_ratio>99_Creatinin>83.67umol/l_age≤54y.o_height>161cm_FII_prothrombin_wt_MTHFR_677_wt_Notch_3_hetero_non diabetes	1	4	33	96	5.4%	0.73 [0.13; 4.06]
PT_second_≤13.25seconds_PT_ratio>99_Creatinin≤83.67umol/l_age>54y.o_height≤161cm_FII_prothrombin_wt_MTHFR_677_wt_Notch_3_hetero_non diabetes	1	4	33	96	5.4%	0.73 [0.13; 4.06]
PT_second_>13.25seconds_PT_ratio≤99_Creatinin>83.67umol/l_age>54y.o_height>161cm_FII_prothrombin_wt_MTHFR_677_wt_Notch_3_hetero_non diabetes	1	3	33	97	4.0%	0.98 [0.19; 4.97]
PT_second_>13.25seconds_PT_ratio≤99_Creatinin≤83.67umol/l_age>54y.o_height>161cm_FII_prothrombin_wt_MTHFR_677_wt_Notch_3_hetero_non diabetes	1	3	33	97	4.0%	0.98 [0.19; 4.97]
PT_second_>13.25seconds_PT_ratio≤99_Creatinin≤83.67umol/l_age≤54y.o_height≤161cm_FII_prothrombin_wt_MTHFR_677_wt_Notch_3_hetero_non diabetes	1	3	33	97	4.0%	0.98 [0.19; 4.97]
PT_second_≤13.25seconds_PT_ratio>99_Creatinin≤83.67umol/l_age>54y.o_height≤161cm_FII_prothrombin_wt_MTHFR_677_hetero_Notch_3_hetero_non diabetes	1	3	33	97	4.0%	0.98 [0.19; 4.97]
PT_second_≤13.25seconds_PT_ratio>99_Creatinin≤83.67umol/l_age≤54y.o_height>161cm_FII_prothrombin_wt_MTHFR_677_wt_Notch_3_hetero_non diabetes	1	3	33	97	4.0%	0.98 [0.19; 4.97]
PT_second_>13.25seconds_PT_ratio≤99_Creatinin≤83.67umol/l_age≤54y.o_height≤161cm_FII_prothrombin_wt_MTHFR_677_hetero_Notch_3_hetero_non diabetes	1	2	33	98	2.7%	1.48 [0.36; 6.10]
PT_second_≤13.25seconds_PT_ratio>99_Creatinin≤83.67umol/l_age>54y.o_height>161cm_FII_prothrombin_wt_MTHFR_677_wt_Notch_3_hetero_non diabetes	1	2	33	98	2.7%	1.48 [0.36; 6.10]
PT_second_≤13.25seconds_PT_ratio≤99_Creatinin>83.67umol/l_age>54y.o_height>161cm_FII_prothrombin_wt_MTHFR_677_wt_Notch_3_hetero_non diabetes	1	2	33	98	2.7%	1.48 [0.36; 6.10]
PT_second_≤13.25seconds_PT_ratio≤99_Creatinin≤83.67umol/l_age≤54y.o_height>161cm_FII_prothrombin_wt_MTHFR_677_hetero_Notch_3_hetero_non diabetes	1	2	33	98	2.7%	1.48 [0.36; 6.10]
PT_second_>13.25seconds_PT_ratio≤99_Creatinin>83.67umol/l_age>54y.o_height>161cm_FII_prothrombin_wt_MTHFR_677_hetero_Notch_3_hetero_non diabetes	2	4	32	96	5.2%	1.50 [0.54; 4.16]
PT_second_>13.25seconds_PT_ratio≤99_Creatinin>83.67umol/l_age>54y.o_height≤161cm_FII_prothrombin_wt_MTHFR_677_wt_Notch_3_hetero_non diabetes	3	5	31	95	6.3%	1.84 [0.85; 3.98]
PT_second_>13.25seconds_PT_ratio>99_Creatinin≤83.67umol/l_age>54y.o_height≤161cm_FII_prothrombin_wt_MTHFR_677_wt_Notch_3_hetero_non diabetes	1	1	33	99	2.7%	2.97 [2.25; 3.91]
PT_second_>13.25seconds_PT_ratio≤99_Creatinin>83.67umol/l_age>54y.o_height≤161cm_FII_prothrombin_wt_MTHFR_677_homo_Notch_3_hetero_non diabetes	1	1	33	99	2.7%	2.97 [2.25; 3.91]
PT_second_>13.25seconds_PT_ratio≤99_Creatinin≤83.67umol/l_age>54y.o_height≤161cm_FII_prothrombin_wt_MTHFR_677_hetero_Notch_3_hetero_diabetes	1	1	33	99	2.7%	2.97 [2.25; 3.91]
PT_second_>13.25seconds_PT_ratio≤99_Creatinin≤83.67umol/l_age>54y.o_height≤161cm_FII_prothrombin_wt_MTHFR_677_wt_Notch_3_hetero_non diabetes	1	1	33	99	2.7%	2.97 [2.25; 3.91]
PT_second_>13.25seconds_PT_ratio≤99_Creatinin≤83.67umol/l_age≤54y.o_height>161cm_FII_prothrombin_wt_MTHFR_677_hetero_Notch_3_hetero_non diabetes	1	1	33	99	2.7%	2.97 [2.25; 3.91]
PT_second_>13.25seconds_PT_ratio≤99_Creatinin≤83.67umol/l_age≤54y.o_height>161cm_FII_prothrombin_wt_MTHFR_677_wt_Notch_3_wt_non diabetes	1	1	33	99	2.7%	2.97 [2.25; 3.91]
PT_second_≤13.25seconds_PT_ratio>99_Creatinin>83.67umol/l_age>54y.o_height>161cm_FII_prothrombin_wt_MTHFR_677_hetero_Notch_3_hetero_diabetes	1	1	33	99	2.7%	2.97 [2.25; 3.91]
PT_second_≤13.25seconds_PT_ratio>99_Creatinin>83.67umol/l_age>54y.o_height>161cm_FII_prothrombin_wt_MTHFR_677_wt_Notch_3_hetero_non diabetes	1	1	33	99	2.7%	2.97 [2.25; 3.91]
PT_second_≤13.25seconds_PT_ratio>99_Creatinin>83.67umol/l_age>54y.o_height≤161cm_FII_prothrombin_wt_MTHFR_677_hetero_Notch_3_hetero_non diabetes	1	1	33	99	2.7%	2.97 [2.25; 3.91]
PT_second_≤13.25seconds_PT_ratio>99_Creatinin>83.67umol/l_age>54y.o_height≤161cm_FII_prothrombin_wt_MTHFR_677_wt_Notch_3_wt_diabetes	1	1	33	99	2.7%	2.97 [2.25; 3.91]
PT_second_≤13.25seconds_PT_ratio>99_Creatinin>83.67umol/l_age≤54y.o_height>161cm_FII_prothrombin_wt_MTHFR_677_wt_Notch_3_wt_non diabetes	1	1	33	99	2.7%	2.97 [2.25; 3.91]
PT_second_≤13.25seconds_PT_ratio>99_Creatinin>83.67umol/l_age≤54y.o_height≤161cm_FII_prothrombin_wt_MTHFR_677_homo_Notch_3_hetero_non diabetes	1	1	33	99	2.7%	2.97 [2.25; 3.91]
PT_second_≤13.25seconds_PT_ratio≤99_Creatinin>83.67umol/l_age>54y.o_height>161cm_FII_prothrombin_hetero_MTHFR_677_hetero_Notch_3_hetero_non diabetes	1	1	33	99	2.7%	2.97 [2.25; 3.91]
PT_second_≤13.25seconds_PT_ratio≤99_Creatinin>83.67umol/l_age>54y.o_height≤161cm_FII_prothrombin_wt_MTHFR_677_hetero_Notch_3_hetero_non diabetes	1	1	33	99	2.7%	2.97 [2.25; 3.91]
PT_second_≤13.25seconds_PT_ratio≤99_Creatinin≤83.67umol/l_age>54y.o_height>161cm_FII_prothrombin_wt_MTHFR_677_wt_Notch_3_hetero_diabetes	1	1	33	99	2.7%	2.97 [2.25; 3.91]
PT_second_≤13.25seconds_PT_ratio≤99_Creatinin≤83.67umol/l_age>54y.o_height≤161cm_FII_prothrombin_wt_MTHFR_677_hetero_Notch_3_hetero_non diabetes	1	1	33	99	2.7%	2.97 [2.25; 3.91]
PT_second_>13.25seconds_PT_ratio≤99_Creatinin>83.67umol/l_age≤54y.o_height>161cm_FII_prothrombin_wt_MTHFR_677_wt_Notch_3_hetero_non diabetes	2	2	32	98	3.9%	3.03 [2.29; 4.02]
**Total (95% CI)**		**58**		**2942**	**100.0%**	**1.69 [1.38; 2.07]**
**Prediction interval**						**[2.70; 3.11]**

Heterogeneity: $Tau^2 < 0.0001$; $Chi^2 = 20.49$, df = 29 ($P = 0.88$); $I^2 = 0\%$
Test for overall effect: $Z = 5.03$ ($P < 0.01$)

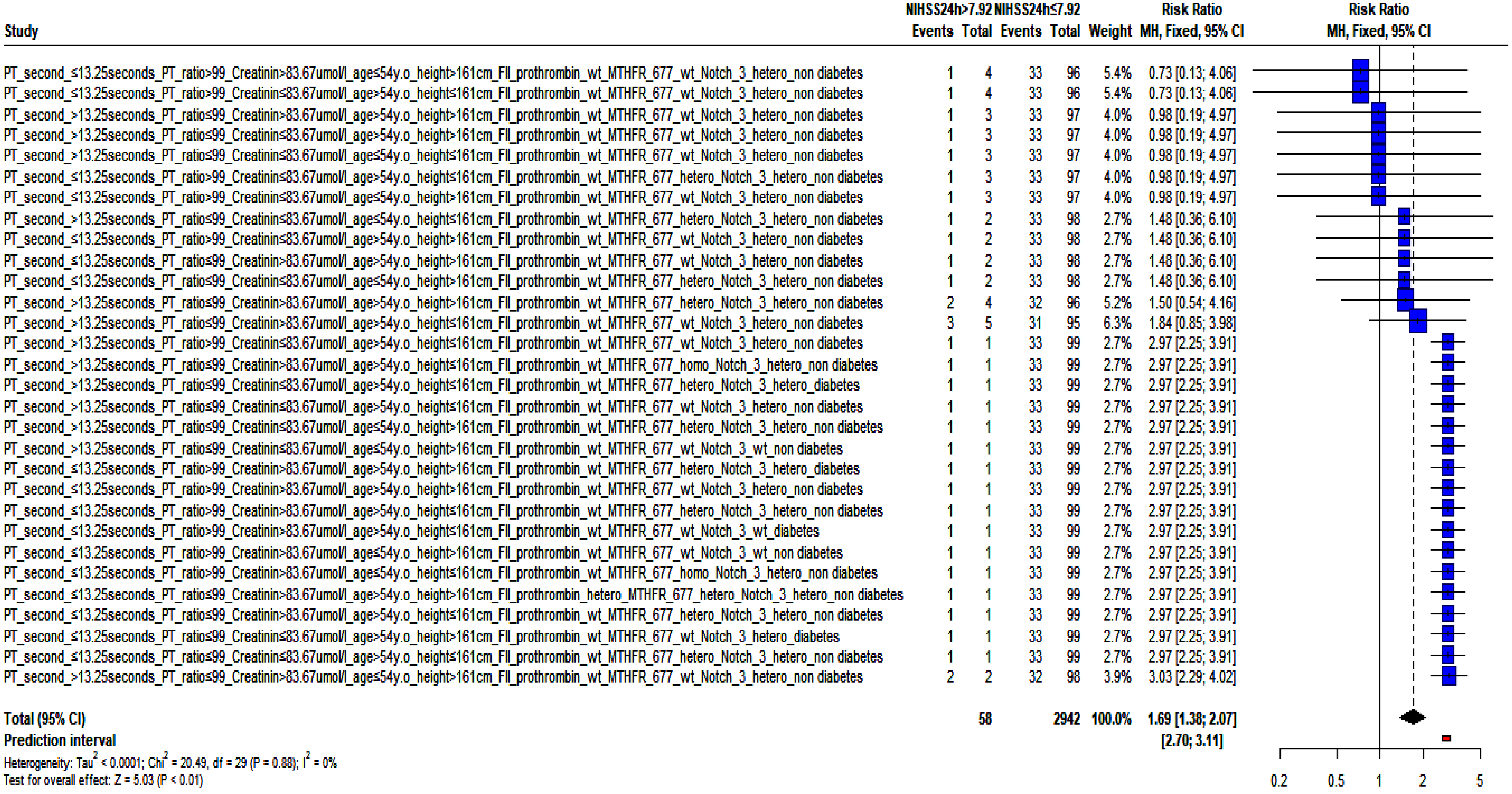

Figure S4. Forest plot of the risk of a National Institutes of Health Stroke Scale (NIHSS) score at discharge >6.85 in cluster 4 (FII Prothrombin genotype, MTHFR-C677T genotype, NOTCH3 p.R544C genotype, and diabetes status) with prothrombin (PT) time and ratio, creatinine, patient age, and patient height.

	NIHSSend>6.85		NIHSSend≤6.85			Risk Ratio
Study	Events	Total	Events	Total	Weight	MH, Fixed, 95% CI
PT_second_>13.25seconds_PT_ratio≤99_Creatinin>83.67umol/l_age>54y.o_height>161cm_FII_prothrombin_wt_MTHFR_677_wt_Notch_3_hetero_non diabetes	1	3	11	97	11.8%	2.94 [0.54; 16.00]
PT_second_>13.25seconds_PT_ratio≤99_Creatinin>83.67umol/l_age>54y.o_height≤161cm_FII_prothrombin_wt_MTHFR_677_wt_Notch_3_hetero_non diabetes	2	5	10	95	17.8%	3.80 [1.12; 12.91]
PT_second_>13.25seconds_PT_ratio≤99_Creatinin>83.67umol/l_age>54y.o_height>161cm_FII_prothrombin_wt_MTHFR_677_hetero_Notch_3_hetero_non diabetes	2	4	10	96	14.2%	4.80 [1.53; 15.04]
PT_second_>13.25seconds_PT_ratio≤99_Creatinin≤83.67umol/l_age>54y.o_height≤161cm_FII_prothrombin_wt_MTHFR_677_hetero_Notch_3_hetero_non diabetes	1	1	11	99	8.0%	8.65 [5.02; 14.90]
PT_second_≤13.25seconds_PT_ratio>99_Creatinin>83.67umol/l_age>54y.o_height>161cm_FII_prothrombin_wt_MTHFR_677_hetero_Notch_3_hetero_diabetes	1	1	11	99	8.0%	8.65 [5.02; 14.90]
PT_second_≤13.25seconds_PT_ratio>99_Creatinin>83.67umol/l_age>54y.o_height>161cm_FII_prothrombin_wt_MTHFR_677_wt_Notch_3_hetero_non diabetes	1	1	11	99	8.0%	8.65 [5.02; 14.90]
PT_second_≤13.25seconds_PT_ratio>99_Creatinin>83.67umol/l_age>54y.o_height≤161cm_FII_prothrombin_wt_MTHFR_677_hetero_Notch_3_hetero_non diabetes	1	1	11	99	8.0%	8.65 [5.02; 14.90]
PT_second_≤13.25seconds_PT_ratio>99_Creatinin>83.67umol/l_age>54y.o_height≤161cm_FII_prothrombin_wt_MTHFR_677_wt_Notch_3_wt_diabetes	1	1	11	99	8.0%	8.65 [5.02; 14.90]
PT_second_≤13.25seconds_PT_ratio>99_Creatinin>83.67umol/l_age≤54y.o_height>161cm_FII_prothrombin_wt_MTHFR_677_wt_Notch_3_wt_non diabetes	1	1	11	99	8.0%	8.65 [5.02; 14.90]
PT_second_≤13.25seconds_PT_ratio≤99_Creatinin>83.67umol/l_age>54y.o_height>161cm_FII_prothrombin_hetero_MTHFR_677_hetero_Notch_3_hetero_non diabetes	1	1	11	99	8.0%	8.65 [5.02; 14.90]
**Total (95% CI)**		**19**		**981**	**100.0%**	**5.37 [3.74; 7.70]**
**Prediction interval**						**[6.49; 10.35]**

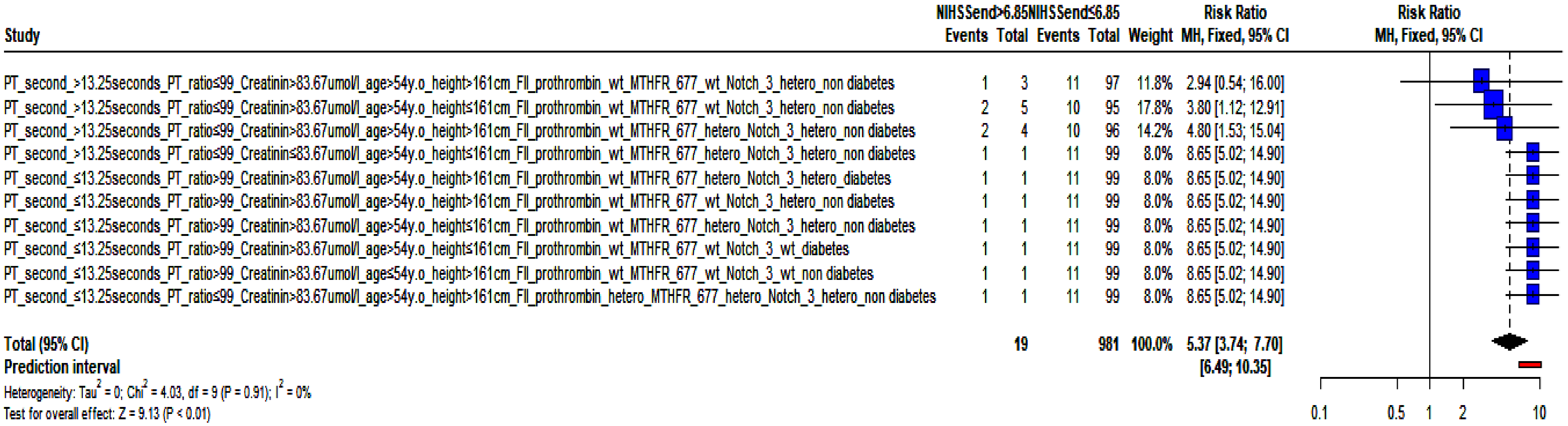

Heterogeneity: $Tau^2 = 0$; $Chi^2 = 4.03$, $df = 9$ ($P = 0.91$); $I^2 = 0\%$
Test for overall effect: $Z = 9.13$ ($P < 0.01$)

Figure S5. Forest plot of the risk of a Glasgow Coma Scale score >12.77 in cluster 4 (FII Prothrombin genotype, MTHFR-C677T genotype, NOTCH3 p.R544C genotype, and diabetes status) with patient BMI.

Study	Glasgow>12.77 Events	Glasgow>12.77 Total	Glasgow≤12.77 Events	Glasgow≤12.77 Total	Weight	Risk Ratio MH, Fixed, 95% CI
BMI≤20.58_FII_prothrombin_wt_MTHFR_677_wt_Notch_3_hetero_non diabetes	6	7	89	93	34.7%	0.90 [0.66; 1.22]
BMI>20.58_FII_prothrombin_wt_MTHFR_677_wt_Notch_3_wt_non diabetes	2	2	93	98	15.3%	1.05 [1.01; 1.10]
BMI>20.58_FII_prothrombin_wt_MTHFR_677_homo_Notch_3_hetero_non diabetes	3	3	92	97	20.2%	1.05 [1.01; 1.10]
BMI>20.58_FII_prothrombin_wt_MTHFR_677_wt_Notch_3_hetero_diabetes	5	5	90	95	29.7%	1.06 [1.01; 1.11]
**Total (95% CI)**		**17**		**383**	**100.0%**	**0.92 [0.78; 1.09]**
**Prediction interval**						**[0.99; 1.12]**

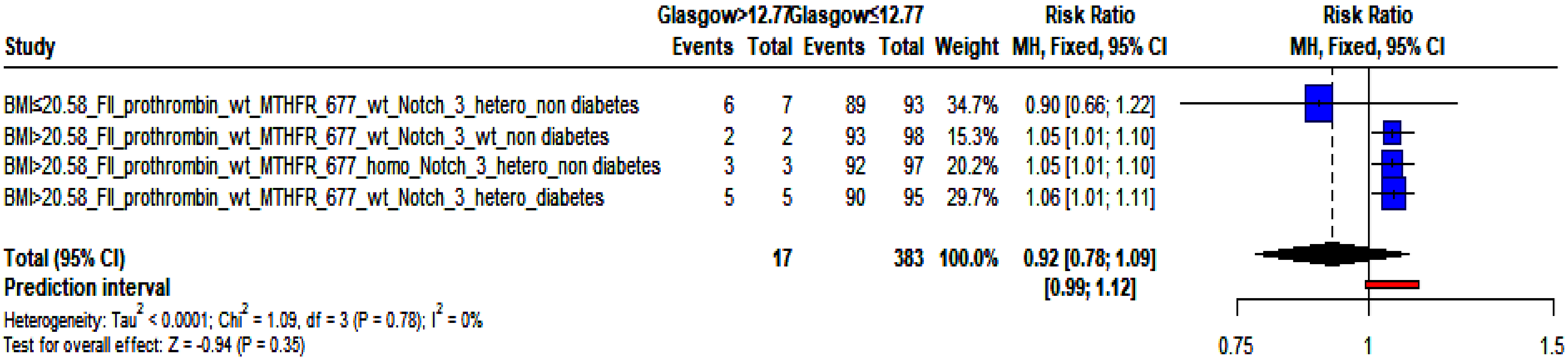

Heterogeneity: $Tau^2 < 0.0001$; $Chi^2 = 1.09$, df = 3 (P = 0.78); $I^2 = 0\%$
Test for overall effect: Z = -0.94 (P = 0.35)

Figure S6. Forest plot of the risk of a modified Rankin scale score >2.86 in cluster 6 (FV-Leiden genotype, PAI1 4G/5G genotype, and FV-Cambridge genotype) with international normalized ratio (INR), prothrombin (PT) time and ratio, creatinine, and number of infarcts on computed tomography (CT).

Study	Rankin>2.86 Events	Rankin>2.86 Total	Rankin≤2.86 Events	Rankin≤2.86 Total	Weight	Risk Ratio MH, Fixed, 95% CI
BMI≤20.58_FII_prothrombin_wt_MTHFR_677_wt_Notch_3_hetero_non diabetes	6	7	89	93	34.7%	0.90 [0.66; 1.22]
BMI>20.58_FII_prothrombin_wt_MTHFR_677_wt_Notch_3_wt_non diabetes	2	2	93	98	15.3%	1.05 [1.01; 1.10]
BMI>20.58_FII_prothrombin_wt_MTHFR_677_homo_Notch_3_hetero_non diabetes	3	3	92	97	20.2%	1.05 [1.01; 1.10]
BMI>20.58_FII_prothrombin_wt_MTHFR_677_wt_Notch_3_hetero_diabetes	5	5	90	95	29.7%	1.06 [1.01; 1.11]
**Total (95% CI)**		**17**		**383**	**100.0%**	**0.92 [0.78; 1.09]**
**Prediction interval**						**[0.99; 1.12]**

Heterogeneity: $Tau^2 < 0.0001$; $Chi^2 = 1.09$, df = 3 (P = 0.78); $I^2 = 0\%$
Test for overall effect: Z = -0.94 (P = 0.35)

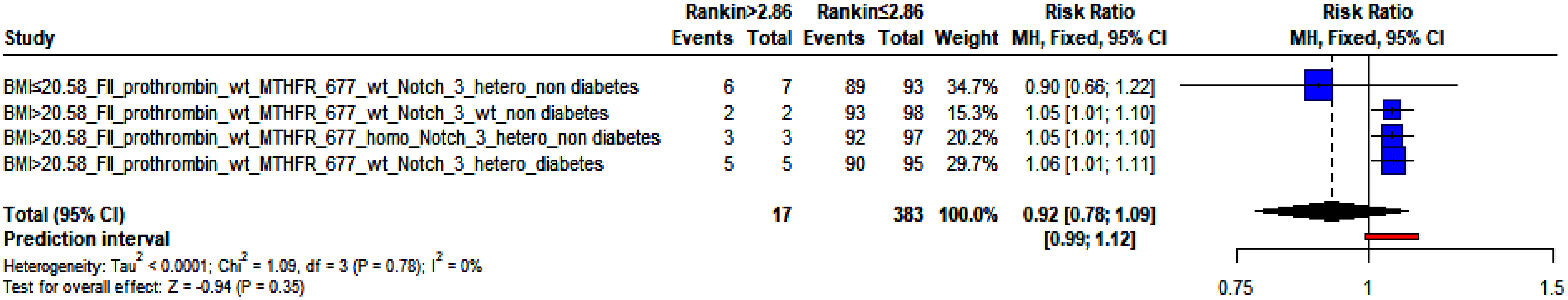

Figure S7. Forest plot of the risk of a National Institutes of Health Stroke Scale (NIHSS) score at admission >9.83 in cluster 6 (FV-Leiden genotype, PAI1 4G/5G genotype, and FV-Cambridge genotype) with prothrombin (PT) time and ratio, creatinine, patient age, and patient height.

Study	NIHSS0h>9.83 Events	NIHSS0h>9.83 Total	NIHSS0h≤9.83 Events	NIHSS0h≤9.83 Total	Weight	Risk Ratio MH, Fixed, 95% CI
PT_second_≤13.25seconds_PT_ratio>99_Creatinin>83.67umol/l_age>54y.o_height≤161cm_FV_Leiden_wt_PAI1_4G_5G_hetero_FV_Cambridge_wt	1	5	26	95	8.3%	0.73 [0.12; 4.35]
PT_second_≤13.25seconds_PT_ratio>99_Creatinin≤83.67umol/l_age>54y.o_height≤161cm_FV_Leiden_wt_PAI1_4G_5G_hetero_FV_Cambridge_wt	1	5	26	95	8.3%	0.73 [0.12; 4.35]
PT_second_>13.25seconds_PT_ratio≤99_Creatinin>83.67umol/l_age>54y.o_height>161cm_FV_Leiden_wt_PAI1_4G_5G_hetero_FV_Cambridge_wt	1	3	26	97	5.0%	1.24 [0.24; 6.37]
PT_second_≤13.25seconds_PT_ratio>99_Creatinin>83.67umol/l_age>54y.o_height≤161cm_FV_Leiden_wt_PAI1_4G_5G_homo_FV_Cambridge_wt	1	3	26	97	5.0%	1.24 [0.24; 6.37]
PT_second_≤13.25seconds_PT_ratio>99_Creatinin≤83.67umol/l_age>54y.o_height≤161cm_FV_Leiden_wt_PAI1_4G_5G_wt_FV_Cambridge_wt	1	3	26	97	5.0%	1.24 [0.24; 6.37]
PT_second_≤13.25seconds_PT_ratio≤99_Creatinin>83.67umol/l_age>54y.o_height>161cm_FV_Leiden_wt_PAI1_4G_5G_hetero_FV_Cambridge_wt	1	3	26	97	5.0%	1.24 [0.24; 6.37]
PT_second_>13.25seconds_PT_ratio≤99_Creatinin>83.67umol/l_age>54y.o_height>161cm_FV_Leiden_wt_PAI1_4G_5G_homo_FV_Cambridge_wt	2	4	25	96	6.4%	1.92 [0.68; 5.41]
PT_second_≤13.25seconds_PT_ratio>99_Creatinin>83.67umol/l_age≤54y.o_height>161cm_FV_Leiden_wt_PAI1_4G_5G_hetero_FV_Cambridge_wt	2	4	25	96	6.4%	1.92 [0.68; 5.41]
PT_second_≤13.25seconds_PT_ratio>99_Creatinin>83.67umol/l_age>54y.o_height>161cm_FV_Leiden_wt_PAI1_4G_5G_hetero_FV_Cambridge_wt	2	3	25	97	4.8%	2.59 [1.09; 6.16]
PT_second_>13.25seconds_PT_ratio≤99_Creatinin>83.67umol/l_age>54y.o_height≤161cm_FV_Leiden_wt_PAI1_4G_5G_wt_FV_Cambridge_wt	1	1	26	99	3.3%	3.75 [2.71; 5.20]
PT_second_>13.25seconds_PT_ratio≤99_Creatinin>83.67umol/l_age≤54y.o_height>161cm_FV_Leiden_wt_PAI1_4G_5G_homo_FV_Cambridge_wt	1	1	26	99	3.3%	3.75 [2.71; 5.20]
PT_second_>13.25seconds_PT_ratio≤99_Creatinin≤83.67umol/l_age>54y.o_height>161cm_FV_Leiden_wt_PAI1_4G_5G_homo_FV_Cambridge_wt	1	1	26	99	3.3%	3.75 [2.71; 5.20]
PT_second_>13.25seconds_PT_ratio≤99_Creatinin≤83.67umol/l_age>54y.o_height≤161cm_FV_Leiden_wt_PAI1_4G_5G_homo_FV_Cambridge_wt	1	1	26	99	3.3%	3.75 [2.71; 5.20]
PT_second_>13.25seconds_PT_ratio≤99_Creatinin≤83.67umol/l_age≤54y.o_height>161cm_FV_Leiden_wt_PAI1_4G_5G_hetero_FV_Cambridge_wt	1	1	26	99	3.3%	3.75 [2.71; 5.20]
PT_second_>13.25seconds_PT_ratio≤99_Creatinin≤83.67umol/l_age≤54y.o_height>161cm_FV_Leiden_wt_PAI1_4G_5G_wt_FV_Cambridge_wt	1	1	26	99	3.3%	3.75 [2.71; 5.20]
PT_second_≤13.25seconds_PT_ratio>99_Creatinin≤83.67umol/l_age>54y.o_height>161cm_FV_Leiden_wt_PAI1_4G_5G_wt_FV_Cambridge_wt	1	1	26	99	3.3%	3.75 [2.71; 5.20]
PT_second_≤13.25seconds_PT_ratio≤99_Creatinin>83.67umol/l_age>54y.o_height>161cm_FV_Leiden_hetero_PAI1_4G_5G_homo_FV_Cambridge_wt	1	1	26	99	3.3%	3.75 [2.71; 5.20]
PT_second_≤13.25seconds_PT_ratio≤99_Creatinin>83.67umol/l_age>54y.o_height>161cm_FV_Leiden_wt_PAI1_4G_5G_homo_FV_Cambridge_wt	1	1	26	99	3.3%	3.75 [2.71; 5.20]
PT_second_≤13.25seconds_PT_ratio≤99_Creatinin>83.67umol/l_age≤54y.o_height>161cm_FV_Leiden_wt_PAI1_4G_5G_hetero_FV_Cambridge_wt	1	1	26	99	3.3%	3.75 [2.71; 5.20]
PT_second_≤13.25seconds_PT_ratio≤99_Creatinin≤83.67umol/l_age>54y.o_height>161cm_FV_Leiden_wt_PAI1_4G_5G_wt_FV_Cambridge_wt	1	1	26	99	3.3%	3.75 [2.71; 5.20]
PT_second_≤13.25seconds_PT_ratio≤99_Creatinin≤83.67umol/l_age≤54y.o_height>161cm_FV_Leiden_hetero_PAI1_4G_5G_wt_FV_Cambridge_wt	1	1	26	99	3.3%	3.75 [2.71; 5.20]
PT_second_>13.25seconds_PT_ratio≤99_Creatinin>83.67umol/l_age>54y.o_height≤161cm_FV_Leiden_wt_PAI1_4G_5G_hetero_FV_Cambridge_wt	3	3	24	97	6.1%	3.98 [2.83; 5.61]
**Total (95% CI)**		**48**		**2152**	**100.0%**	**2.08 [1.64; 2.64]**
**Prediction interval**						**[3.31; 4.00]**

Heterogeneity: $Tau^2 < 0.0001$; $Chi^2 = 17.07$, df = 21 ($P = 0.71$); $I^2 = 0\%$
Test for overall effect: $Z = 6.04$ ($P < 0.01$)

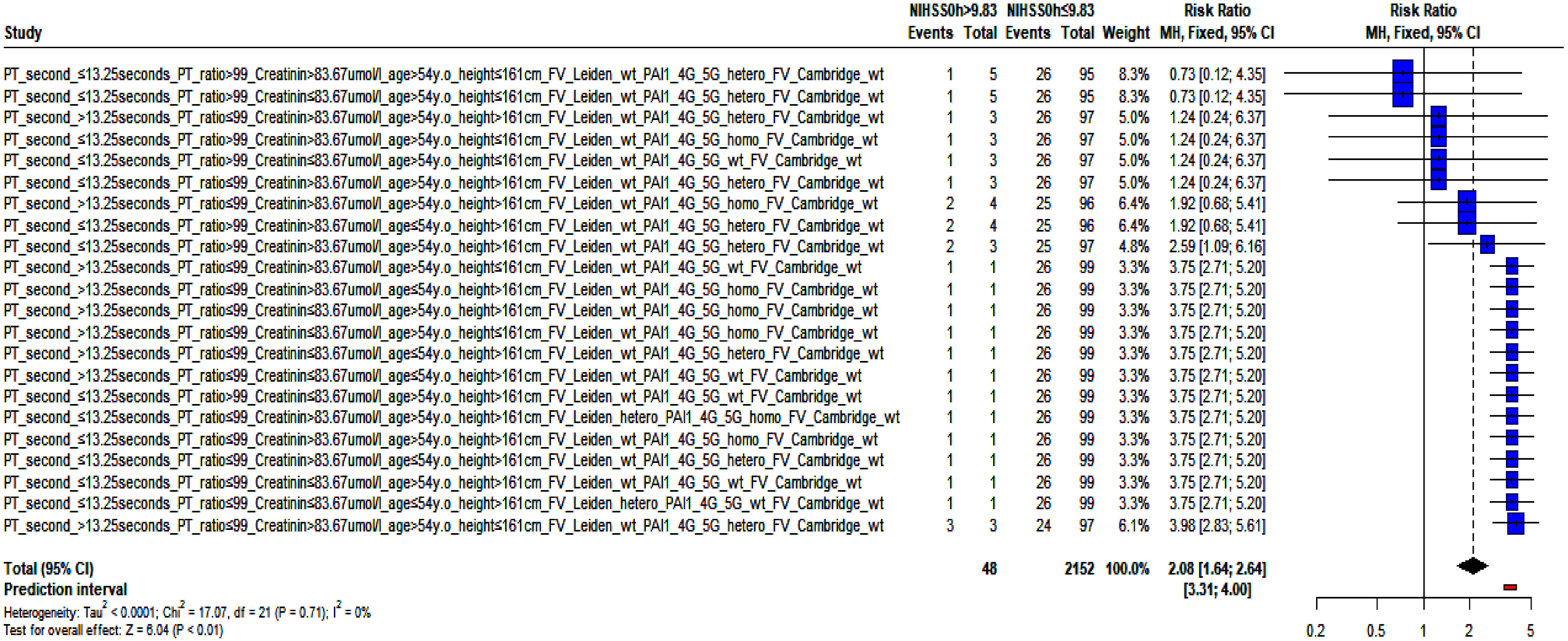

Figure S8. Forest plot of the risk of a National Institutes of Health Stroke Scale (NIHSS) score after 24 hours >7.92 in cluster 6 (FV-Leiden genotype, PAI1 4G/5G genotype, and FV-Cambridge genotype) with prothrombin (PT) time and ratio, creatinine, patient age, and patient height.

Study	NIHSS24h>7.92 Events	NIHSS24h>7.92 Total	NIHSS24h≤7.92 Events	NIHSS24h≤7.92 Total	Weight	Risk Ratio MH, Fixed, 95% CI
PT_second_≤13.25seconds_PT_ratio>99_Creatinin>83.67umol/l_age>54y.o_height≤161cm_FV_Leiden_wt_PAI1_4G_5G_hetero_FV_Cambridge_wt	1	5	33	95	6.7%	0.58 [0.10; 3.40]
PT_second_≤13.25seconds_PT_ratio>99_Creatinin≤83.67umol/l_age≤54y.o_height>161cm_FV_Leiden_wt_PAI1_4G_5G_hetero_FV_Cambridge_wt	1	4	33	96	5.4%	0.73 [0.13; 4.06]
PT_second_>13.25seconds_PT_ratio≤99_Creatinin>83.67umol/l_age>54y.o_height>161cm_FV_Leiden_wt_PAI1_4G_5G_hetero_FV_Cambridge_wt	1	3	33	97	4.0%	0.98 [0.19; 4.97]
PT_second_≤13.25seconds_PT_ratio>99_Creatinin>83.67umol/l_age>54y.o_height≤161cm_FV_Leiden_wt_PAI1_4G_5G_homo_FV_Cambridge_wt	1	3	33	97	4.0%	0.98 [0.19; 4.97]
PT_second_≤13.25seconds_PT_ratio>99_Creatinin≤83.67umol/l_age>54y.o_height≤161cm_FV_Leiden_wt_PAI1_4G_5G_homo_FV_Cambridge_wt	1	3	33	97	4.0%	0.98 [0.19; 4.97]
PT_second_≤13.25seconds_PT_ratio>99_Creatinin≤83.67umol/l_age>54y.o_height≤161cm_FV_Leiden_wt_PAI1_4G_5G_wt_FV_Cambridge_wt	1	3	33	97	4.0%	0.98 [0.19; 4.97]
PT_second_>13.25seconds_PT_ratio≤99_Creatinin≤83.67umol/l_age≤54y.o_height≤161cm_FV_Leiden_wt_PAI1_4G_5G_homo_FV_Cambridge_wt	1	2	33	98	2.7%	1.48 [0.36; 6.10]
PT_second_>13.25seconds_PT_ratio≤99_Creatinin>83.67umol/l_age>54y.o_height>161cm_FV_Leiden_wt_PAI1_4G_5G_homo_FV_Cambridge_wt	2	4	32	96	5.2%	1.50 [0.54; 4.16]
PT_second_≤13.25seconds_PT_ratio>99_Creatinin>83.67umol/l_age≤54y.o_height>161cm_FV_Leiden_wt_PAI1_4G_5G_hetero_FV_Cambridge_wt	2	4	32	96	5.2%	1.50 [0.54; 4.16]
PT_second_>13.25seconds_PT_ratio≤99_Creatinin>83.67umol/l_age>54y.o_height≤161cm_FV_Leiden_wt_PAI1_4G_5G_hetero_FV_Cambridge_wt	2	3	32	97	3.9%	2.02 [0.86; 4.72]
PT_second_≤13.25seconds_PT_ratio>99_Creatinin>83.67umol/l_age>54y.o_height>161cm_FV_Leiden_wt_PAI1_4G_5G_hetero_FV_Cambridge_wt	2	3	32	97	3.9%	2.02 [0.86; 4.72]
PT_second_>13.25seconds_PT_ratio>99_Creatinin≤83.67umol/l_age>54y.o_height≤161cm_FV_Leiden_wt_PAI1_4G_5G_hetero_FV_Cambridge_wt	1	1	33	99	2.7%	2.97 [2.25; 3.91]
PT_second_>13.25seconds_PT_ratio≤99_Creatinin>83.67umol/l_age>54y.o_height≤161cm_FV_Leiden_hetero_PAI1_4G_5G_homo_FV_Cambridge_wt	1	1	33	99	2.7%	2.97 [2.25; 3.91]
PT_second_>13.25seconds_PT_ratio≤99_Creatinin>83.67umol/l_age>54y.o_height≤161cm_FV_Leiden_wt_PAI1_4G_5G_wt_FV_Cambridge_wt	1	1	33	99	2.7%	2.97 [2.25; 3.91]
PT_second_>13.25seconds_PT_ratio≤99_Creatinin>83.67umol/l_age≤54y.o_height>161cm_FV_Leiden_wt_PAI1_4G_5G_hetero_FV_Cambridge_wt	1	1	33	99	2.7%	2.97 [2.25; 3.91]
PT_second_>13.25seconds_PT_ratio≤99_Creatinin>83.67umol/l_age≤54y.o_height>161cm_FV_Leiden_wt_PAI1_4G_5G_homo_FV_Cambridge_wt	1	1	33	99	2.7%	2.97 [2.25; 3.91]
PT_second_>13.25seconds_PT_ratio≤99_Creatinin≤83.67umol/l_age>54y.o_height>161cm_FV_Leiden_wt_PAI1_4G_5G_homo_FV_Cambridge_wt	1	1	33	99	2.7%	2.97 [2.25; 3.91]
PT_second_>13.25seconds_PT_ratio≤99_Creatinin≤83.67umol/l_age>54y.o_height≤161cm_FV_Leiden_hetero_PAI1_4G_5G_hetero_FV_Cambridge_wt	1	1	33	99	2.7%	2.97 [2.25; 3.91]
PT_second_>13.25seconds_PT_ratio≤99_Creatinin≤83.67umol/l_age>54y.o_height≤161cm_FV_Leiden_wt_PAI1_4G_5G_homo_FV_Cambridge_wt	1	1	33	99	2.7%	2.97 [2.25; 3.91]
PT_second_>13.25seconds_PT_ratio≤99_Creatinin≤83.67umol/l_age≤54y.o_height>161cm_FV_Leiden_wt_PAI1_4G_5G_hetero_FV_Cambridge_wt	1	1	33	99	2.7%	2.97 [2.25; 3.91]
PT_second_>13.25seconds_PT_ratio≤99_Creatinin≤83.67umol/l_age≤54y.o_height>161cm_FV_Leiden_wt_PAI1_4G_5G_wt_FV_Cambridge_wt	1	1	33	99	2.7%	2.97 [2.25; 3.91]
PT_second_>13.25seconds_PT_ratio≤99_Creatinin≤83.67umol/l_age≤54y.o_height≤161cm_FV_Leiden_wt_PAI1_4G_5G_wt_FV_Cambridge_wt	1	1	33	99	2.7%	2.97 [2.25; 3.91]
PT_second_≤13.25seconds_PT_ratio>99_Creatinin>83.67umol/l_age≤54y.o_height≤161cm_FV_Leiden_wt_PAI1_4G_5G_wt_FV_Cambridge_wt	1	1	33	99	2.7%	2.97 [2.25; 3.91]
PT_second_≤13.25seconds_PT_ratio>99_Creatinin≤83.67umol/l_age>54y.o_height>161cm_FV_Leiden_wt_PAI1_4G_5G_wt_FV_Cambridge_wt	1	1	33	99	2.7%	2.97 [2.25; 3.91]
PT_second_≤13.25seconds_PT_ratio≤99_Creatinin>83.67umol/l_age>54y.o_height>161cm_FV_Leiden_hetero_PAI1_4G_5G_homo_FV_Cambridge_wt	1	1	33	99	2.7%	2.97 [2.25; 3.91]
PT_second_≤13.25seconds_PT_ratio≤99_Creatinin>83.67umol/l_age>54y.o_height>161cm_FV_Leiden_wt_PAI1_4G_5G_homo_FV_Cambridge_wt	1	1	33	99	2.7%	2.97 [2.25; 3.91]
PT_second_≤13.25seconds_PT_ratio≤99_Creatinin>83.67umol/l_age>54y.o_height≤161cm_FV_Leiden_wt_PAI1_4G_5G_hetero_FV_Cambridge_wt	1	1	33	99	2.7%	2.97 [2.25; 3.91]
PT_second_≤13.25seconds_PT_ratio≤99_Creatinin≤83.67umol/l_age>54y.o_height>161cm_FV_Leiden_wt_PAI1_4G_5G_wt_FV_Cambridge_wt	1	1	33	99	2.7%	2.97 [2.25; 3.91]
PT_second_≤13.25seconds_PT_ratio≤99_Creatinin≤83.67umol/l_age>54y.o_height≤161cm_FV_Leiden_wt_PAI1_4G_5G_wt_FV_Cambridge_wt	1	1	33	99	2.7%	2.97 [2.25; 3.91]
PT_second_≤13.25seconds_PT_ratio≤99_Creatinin≤83.67umol/l_age≤54y.o_height>161cm_FV_Leiden_hetero_PAI1_4G_5G_wt_FV_Cambridge_wt	1	1	33	99	2.7%	2.97 [2.25; 3.91]
**Total (95% CI)**		**56**		**2944**	**100.0%**	**1.73 [1.42; 2.11]**
**Prediction interval**						**[2.72; 3.10]**

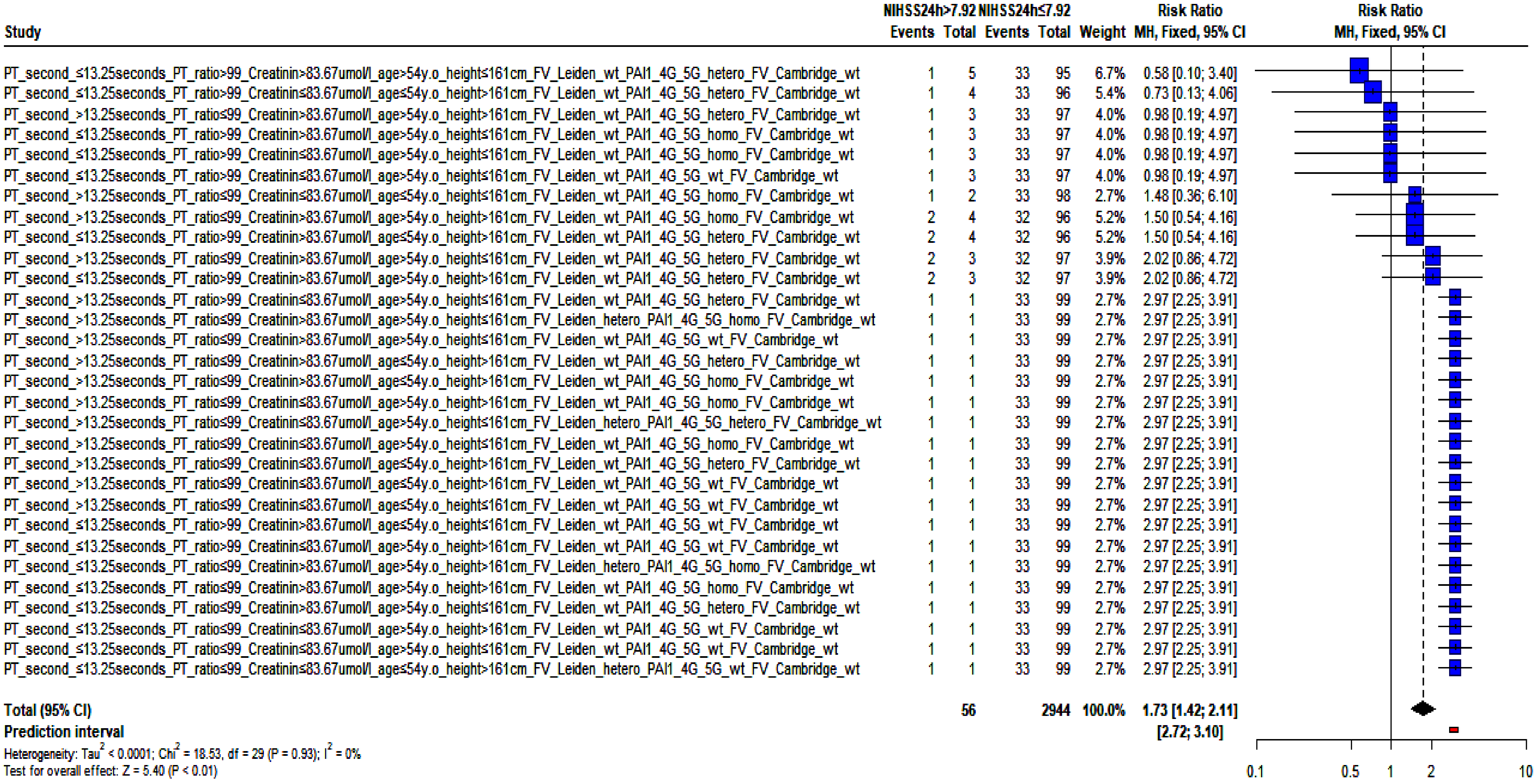

Heterogeneity: $Tau^2 < 0.0001$; $Chi^2 = 18.53$, df = 29 ($P = 0.93$); $I^2 = 0\%$
Test for overall effect: $Z = 5.40$ ($P < 0.01$)

Figure S9. Forest plot of the risk of a Glasgow Coma Scale score >12.77 in cluster 6 (FV-Leiden genotype, PAI1 4G/5G genotype, and FV-Cambridge genotype) with BMI.

	Glasgow>12.77		Glasgow≤12.77			Risk Ratio
**Study**	**Events**	**Total**	**Events**	**Total**	**Weight**	**MH, Fixed, 95% CI**
BMI≤20.58_FV_Leiden_wt_PAI1_4G_5G_hetero_FV_Cambridge_wt	9	11	86	89	12.2%	0.85 [0.64; 1.12]
BMI>20.58_FV_Leiden_wt_PAI1_4G_5G_hetero_FV_Cambridge_wt	31	32	64	68	26.4%	1.03 [0.94; 1.12]
BMI>20.58_FV_Leiden_hetero_PAI1_4G_5G_hetero_FV_Cambridge_wt	1	1	94	99	2.4%	1.05 [1.01; 1.10]
BMI>20.58_FV_Leiden_hetero_PAI1_4G_5G_wt_FV_Cambridge_wt	1	1	94	99	2.4%	1.05 [1.01; 1.10]
BMI≤20.58_FV_Leiden_hetero_PAI1_4G_5G_wt_FV_Cambridge_wt	1	1	94	99	2.4%	1.05 [1.01; 1.10]
BMI>20.58_FV_Leiden_hetero_PAI1_4G_5G_homo_FV_Cambridge_wt	3	3	92	97	4.7%	1.05 [1.01; 1.10]
BMI≤20.58_FV_Leiden_wt_PAI1_4G_5G_homo_FV_Cambridge_wt	5	5	90	95	6.9%	1.06 [1.01; 1.11]
BMI>20.58_FV_Leiden_wt_PAI1_4G_5G_wt_FV_Cambridge_wt	21	21	74	79	20.7%	1.07 [1.01; 1.13]
BMI>20.58_FV_Leiden_wt_PAI1_4G_5G_homo_FV_Cambridge_wt	23	23	72	77	22.0%	1.07 [1.01; 1.13]
**Total (95% CI)**		**98**		**802**	**100.0%**	**0.99 [0.93; 1.05]**
**Prediction interval**						**[1.03; 1.08]**

Heterogeneity: $Tau^2 < 0.0001$; $Chi^2 = 3.03$, df = 8 (P = 0.93); $I^2 = 0\%$
Test for overall effect: Z = -0.29 (P = 0.77)

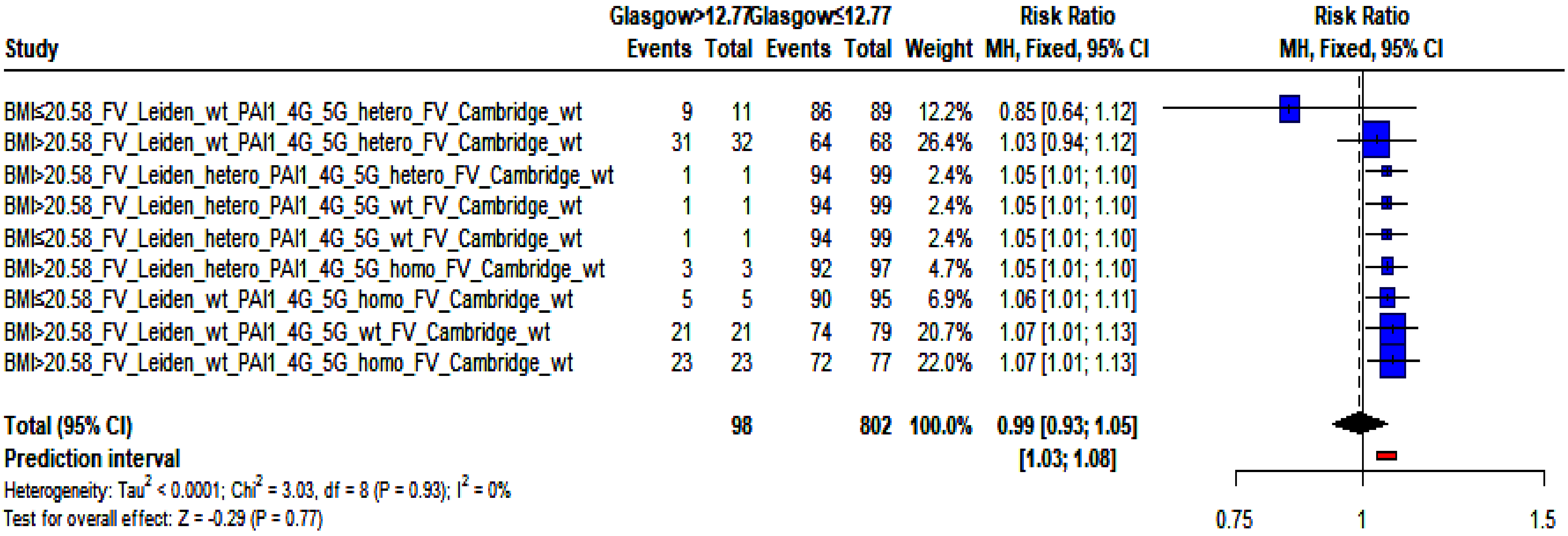

Figure S10. Forest plot of the risk of a modified Rankin scale score >2.86 in cluster 11 (MTHFR-A1298C genotype and FV-H1299R genotype) with international normalized ratio (INR), prothrombin (PT) time and ratio, creatinine, and number of infarcts on computed tomography (CT).

Study	Rankin>2.86 Events	Rankin>2.86 Total	Rankin≤2.86 Events	Rankin≤2.86 Total	Weight	Risk Ratio MH, Fixed, 95% CI
INR>1.02_PT_second_>13.25seconds_PT_ratio≤99_Creatinin≤83.67umol/l_N_infarctsCT≤2_MTHFR_1298_wt_FV_1299_wt	1	8	25	92	12.3%	0.46 [0.07; 2.97]
INR≤1.02_PT_second_≤13.25seconds_PT_ratio>99_Creatinin>83.67umol/l_N_infarctsCT≤2_MTHFR_1298_hetero_FV_1299_wt	1	6	25	94	9.3%	0.63 [0.10; 3.87]
INR≤1.02_PT_second_≤13.25seconds_PT_ratio>99_Creatinin>83.67umol/l_N_infarctsCT>2_MTHFR_1298_wt_FV_1299_wt	1	5	25	95	7.7%	0.76 [0.13; 4.53]
INR≤1.02_PT_second_≤13.25seconds_PT_ratio>99_Creatinin>83.67umol/l_N_infarctsCT≤2_MTHFR_1298_wt_FV_1299_wt	2	8	24	92	11.9%	0.96 [0.27; 3.34]
INR≤1.02_PT_second_≤13.25seconds_PT_ratio≤99_Creatinin>83.67umol/l_N_infarctsCT≤2_MTHFR_1298_wt_FV_1299_wt	1	4	25	96	6.2%	0.96 [0.17; 5.42]
INR>1.02_PT_second_>13.25seconds_PT_ratio≤99_Creatinin≤83.67umol/l_N_infarctsCT≤2_MTHFR_1298_hetero_FV_1299_wt	1	3	25	97	4.6%	1.29 [0.25; 6.64]
INR>1.02_PT_second_≤13.25seconds_PT_ratio≤99_Creatinin≤83.67umol/l_N_infarctsCT≤2_MTHFR_1298_hetero_FV_1299_wt	1	2	25	98	3.1%	1.96 [0.47; 8.16]
INR≤1.02_PT_second_≤13.25seconds_PT_ratio>99_Creatinin>83.67umol/l_N_infarctsCT>2_MTHFR_1298_hetero_FV_1299_wt	1	2	25	98	3.1%	1.96 [0.47; 8.16]
INR≤1.02_PT_second_≤13.25seconds_PT_ratio≤99_Creatinin≤83.67umol/l_N_infarctsCT>2_MTHFR_1298_wt_FV_1299_wt	1	2	25	98	3.1%	1.96 [0.47; 8.16]
INR>1.02_PT_second_>13.25seconds_PT_ratio≤99_Creatinin>83.67umol/l_N_infarctsCT≤2_MTHFR_1298_hetero_FV_1299_wt	3	4	23	96	5.7%	3.13 [1.60; 6.11]
INR>1.02_PT_second_>13.25seconds_PT_ratio≤99_Creatinin>83.67umol/l_N_infarctsCT≤2_MTHFR_1298_wt_FV_1299_wt	3	4	23	96	5.7%	3.13 [1.60; 6.11]
INR>1.02_PT_second_>13.25seconds_PT_ratio≤99_Creatinin>83.67umol/l_N_infarctsCT>2_MTHFR_1298_hetero_FV_1299_wt	1	1	25	99	3.1%	3.90 [2.79; 5.45]
INR>1.02_PT_second_>13.25seconds_PT_ratio≤99_Creatinin>83.67umol/l_N_infarctsCT>2_MTHFR_1298_homo_FV_1299_wt	1	1	25	99	3.1%	3.90 [2.79; 5.45]
INR>1.02_PT_second_>13.25seconds_PT_ratio≤99_Creatinin≤83.67umol/l_N_infarctsCT>2_MTHFR_1298_homo_FV_1299_wt	1	1	25	99	3.1%	3.90 [2.79; 5.45]
INR≤1.02_PT_second_>13.25seconds_PT_ratio≤99_Creatinin>83.67umol/l_N_infarctsCT>2_MTHFR_1298_hetero_FV_1299_hetero	1	1	25	99	3.1%	3.90 [2.79; 5.45]
INR≤1.02_PT_second_>13.25seconds_PT_ratio≤99_Creatinin>83.67umol/l_N_infarctsCT>2_MTHFR_1298_wt_FV_1299_wt	1	1	25	99	3.1%	3.90 [2.79; 5.45]
INR≤1.02_PT_second_≤13.25seconds_PT_ratio>99_Creatinin≤83.67umol/l_N_infarctsCT>2_MTHFR_1298_wt_FV_1299_wt	1	1	25	99	3.1%	3.90 [2.79; 5.45]
INR>1.02_PT_second_>13.25seconds_PT_ratio≤99_Creatinin>83.67umol/l_N_infarctsCT>2_MTHFR_1298_wt_FV_1299_wt	2	2	24	98	4.4%	4.02 [2.85; 5.67]
INR≤1.02_PT_second_≤13.25seconds_PT_ratio≤99_Creatinin>83.67umol/l_N_infarctsCT≤2_MTHFR_1298_hetero_FV_1299_wt	2	2	24	98	4.4%	4.02 [2.85; 5.67]
**Total (95% CI)**		**58**		**1842**	**100.0%**	**1.79 [1.36; 2.35]**
**Prediction interval**						**[3.25; 4.14]**

Heterogeneity: $Tau^2 < 0.0001$; $Chi^2 = 23.67$, df = 18 ($P = 0.17$); $I^2 = 24\%$
Test for overall effect: $Z = 4.16$ ($P < 0.01$)

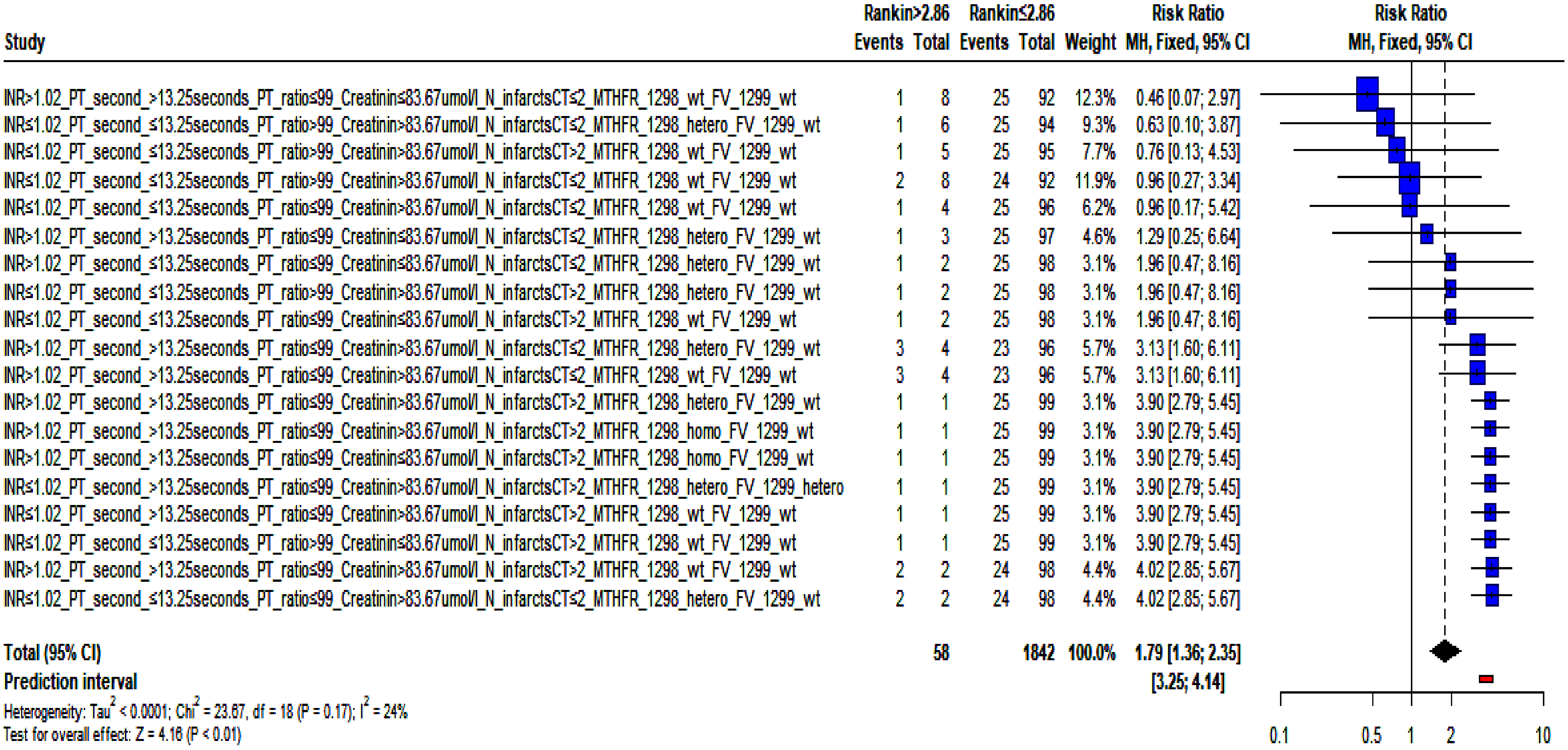

Figure S11. Forest plot of the risk of a National Institutes of Health Stroke Scale (NIHSS) score at admission >9.83 in cluster 11 (MTHFR-A1298C genotype and FV-H1299R genotype) with prothrombin (PT) time and ratio, creatinine, patient age, and patient height.

Study	NIHSS0h>9.83 Events	NIHSS0h>9.83 Total	NIHSS0h≤9.83 Events	NIHSS0h≤9.83 Total	Weight	Risk Ratio MH, Fixed, 95% CI
PT_second_≤13.25seconds_PT_ratio>99_Creatinin>83.67umol/l_age>54y.o_height≤161cm_MTHFR_1298_wt_FV_1299_wt	1	7	26	93	10.5%	0.51 [0.08; 3.23]
PT_second_≤13.25seconds_PT_ratio>99_Creatinin≤83.67umol/l_age>54y.o_height≤161cm_MTHFR_1298_wt_FV_1299_wt	1	6	26	94	9.0%	0.60 [0.10; 3.71]
PT_second_≤13.25seconds_PT_ratio>99_Creatinin≤83.67umol/l_age>54y.o_height≤161cm_MTHFR_1298_hetero_FV_1299_wt	1	5	26	95	7.5%	0.73 [0.12; 4.35]
PT_second_≤13.25seconds_PT_ratio>99_Creatinin≤83.67umol/l_age>54y.o_height>161cm_MTHFR_1298_wt_FV_1299_wt	1	4	26	96	6.0%	0.92 [0.16; 5.20]
PT_second_>13.25seconds_PT_ratio≤99_Creatinin>83.67umol/l_age>54y.o_height>161cm_MTHFR_1298_hetero_FV_1299_wt	1	3	26	97	4.5%	1.24 [0.24; 6.37]
PT_second_>13.25seconds_PT_ratio≤99_Creatinin>83.67umol/l_age≤54y.o_height>161cm_MTHFR_1298_wt_FV_1299_wt	1	3	26	97	4.5%	1.24 [0.24; 6.37]
PT_second_≤13.25seconds_PT_ratio>99_Creatinin>83.67umol/l_age>54y.o_height≤161cm_MTHFR_1298_hetero_FV_1299_wt	1	3	26	97	4.5%	1.24 [0.24; 6.37]
PT_second_≤13.25seconds_PT_ratio≤99_Creatinin>83.67umol/l_age>54y.o_height>161cm_MTHFR_1298_wt_FV_1299_wt	1	3	26	97	4.5%	1.24 [0.24; 6.37]
PT_second_>13.25seconds_PT_ratio≤99_Creatinin>83.67umol/l_age>54y.o_height>161cm_MTHFR_1298_wt_FV_1299_wt	1	2	26	98	3.0%	1.88 [0.45; 7.83]
PT_second_>13.25seconds_PT_ratio≤99_Creatinin≤83.67umol/l_age>54y.o_height>161cm_MTHFR_1298_wt_FV_1299_wt	1	2	26	98	3.0%	1.88 [0.45; 7.83]
PT_second_≤13.25seconds_PT_ratio≤99_Creatinin≤83.67umol/l_age>54y.o_height>161cm_MTHFR_1298_wt_FV_1299_wt	1	2	26	98	3.0%	1.88 [0.45; 7.83]
PT_second_>13.25seconds_PT_ratio≤99_Creatinin>83.67umol/l_age>54y.o_height≤161cm_MTHFR_1298_hetero_FV_1299_wt	2	3	25	97	4.3%	2.59 [1.09; 6.16]
PT_second_>13.25seconds_PT_ratio≤99_Creatinin>83.67umol/l_age>54y.o_height≤161cm_MTHFR_1298_wt_FV_1299_wt	2	3	25	97	4.3%	2.59 [1.09; 6.16]
PT_second_≤13.25seconds_PT_ratio>99_Creatinin>83.67umol/l_age≤54y.o_height>161cm_MTHFR_1298_hetero_FV_1299_wt	2	3	25	97	4.3%	2.59 [1.09; 6.16]
PT_second_>13.25seconds_PT_ratio≤99_Creatinin>83.67umol/l_age>54y.o_height>161cm_MTHFR_1298_hetero_FV_1299_hetero	1	1	26	99	3.0%	3.75 [2.71; 5.20]
PT_second_>13.25seconds_PT_ratio≤99_Creatinin≤83.67umol/l_age>54y.o_height≤161cm_MTHFR_1298_homo_FV_1299_wt	1	1	26	99	3.0%	3.75 [2.71; 5.20]
PT_second_>13.25seconds_PT_ratio≤99_Creatinin≤83.67umol/l_age≤54y.o_height>161cm_MTHFR_1298_homo_FV_1299_hetero	1	1	26	99	3.0%	3.75 [2.71; 5.20]
PT_second_>13.25seconds_PT_ratio≤99_Creatinin≤83.67umol/l_age≤54y.o_height>161cm_MTHFR_1298_wt_FV_1299_wt	1	1	26	99	3.0%	3.75 [2.71; 5.20]
PT_second_≤13.25seconds_PT_ratio≤99_Creatinin>83.67umol/l_age≤54y.o_height>161cm_MTHFR_1298_wt_FV_1299_wt	1	1	26	99	3.0%	3.75 [2.71; 5.20]
PT_second_≤13.25seconds_PT_ratio≤99_Creatinin≤83.67umol/l_age≤54y.o_height>161cm_MTHFR_1298_wt_FV_1299_wt	1	1	26	99	3.0%	3.75 [2.71; 5.20]
PT_second_≤13.25seconds_PT_ratio>99_Creatinin>83.67umol/l_age>54y.o_height>161cm_MTHFR_1298_wt_FV_1299_wt	2	2	25	98	4.3%	3.86 [2.77; 5.40]
PT_second_≤13.25seconds_PT_ratio≤99_Creatinin>83.67umol/l_age>54y.o_height>161cm_MTHFR_1298_hetero_FV_1299_wt	2	2	25	98	4.3%	3.86 [2.77; 5.40]
**Total (95% CI)**		**59**		**2141**	**100.0%**	**1.74 [1.34; 2.27]**
**Prediction interval**						**[3.11; 3.94]**

Heterogeneity: $Tau^2 < 0.0001$; $Chi^2 = 24.50$, df = 21 ($P = 0.27$); $I^2 = 14\%$
Test for overall effect: $Z = 4.09$ ($P < 0.01$)

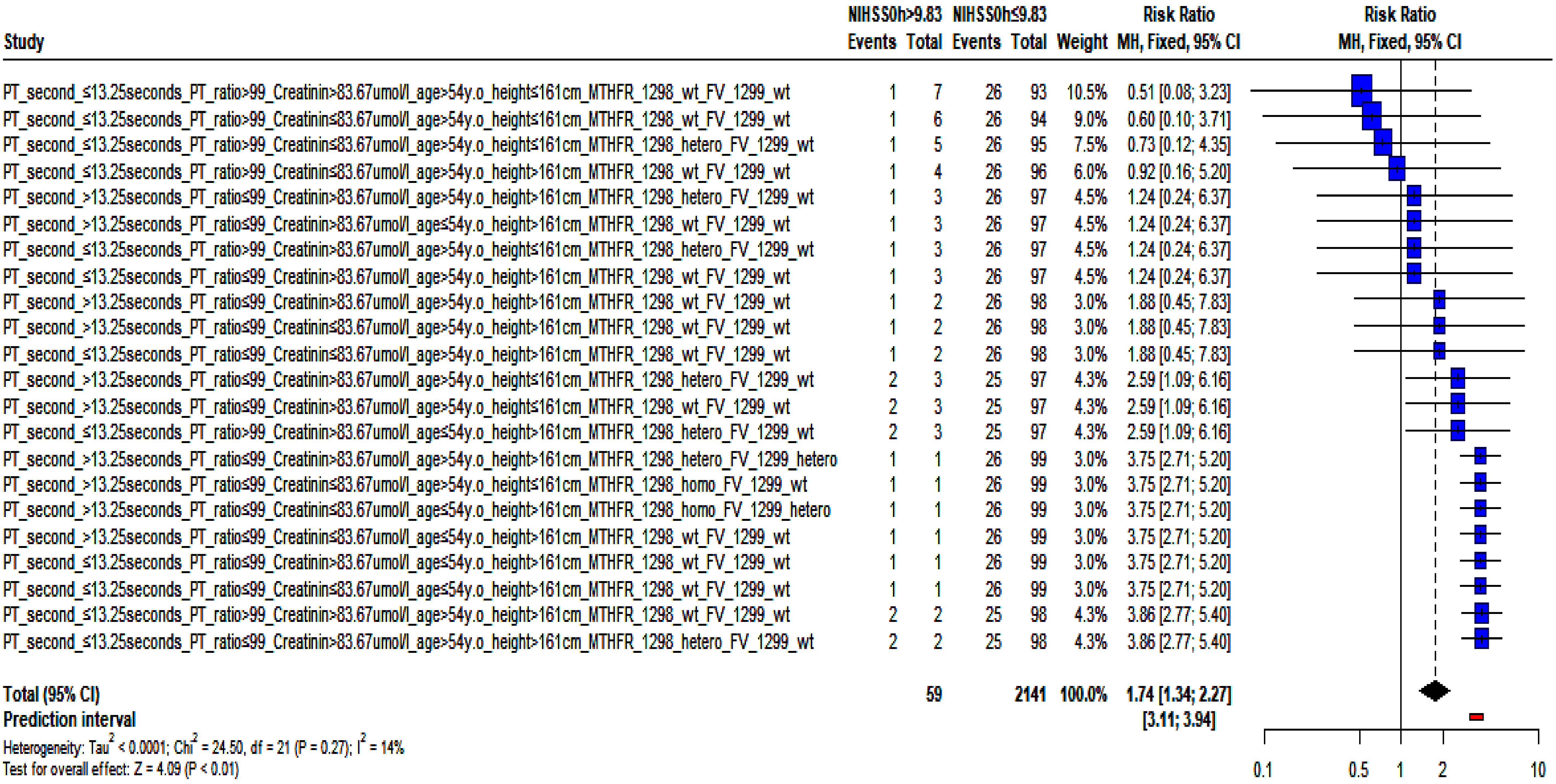

Figure S12. Forest plot of the risk of a National Institutes of Health Stroke Scale (NIHSS) score after 24 hours >7.92 in cluster 11 (MTHFR-A1298C genotype and FV-H1299R genotype) with prothrombin (PT) time and ratio, creatinine, patient age, and patient height.

Study	NIHSS24h>7.92 Events	NIHSS24h>7.92 Total	NIHSS24h≤7.92 Events	NIHSS24h≤7.92 Total	Weight	Risk Ratio MH, Fixed, 95% CI
PT_second_≤13.25seconds_PT_ratio>99_Creatinin>83.67umol/l_age>54y.o_height≤161cm_MTHFR_1298_wt_FV_1299_wt	1	7	33	93	9.5%	0.40 [0.06; 2.52]
PT_second_≤13.25seconds_PT_ratio>99_Creatinin≤83.67umol/l_age>54y.o_height>161cm_MTHFR_1298_wt_FV_1299_wt	1	4	33	96	5.4%	0.73 [0.13; 4.06]
PT_second_≤13.25seconds_PT_ratio>99_Creatinin≤83.67umol/l_age>54y.o_height≤161cm_MTHFR_1298_wt_FV_1299_wt	2	6	32	94	7.9%	0.98 [0.31; 3.14]
PT_second_>13.25seconds_PT_ratio≤99_Creatinin>83.67umol/l_age>54y.o_height>161cm_MTHFR_1298_hetero_FV_1299_wt	1	3	33	97	4.1%	0.98 [0.19; 4.97]
PT_second_>13.25seconds_PT_ratio≤99_Creatinin>83.67umol/l_age>54y.o_height≤161cm_MTHFR_1298_hetero_FV_1299_wt	1	3	33	97	4.1%	0.98 [0.19; 4.97]
PT_second_≤13.25seconds_PT_ratio>99_Creatinin>83.67umol/l_age>54y.o_height≤161cm_MTHFR_1298_hetero_FV_1299_wt	1	3	33	97	4.1%	0.98 [0.19; 4.97]
PT_second_≤13.25seconds_PT_ratio>99_Creatinin≤83.67umol/l_age≤54y.o_height>161cm_MTHFR_1298_wt_FV_1299_wt	1	3	33	97	4.1%	0.98 [0.19; 4.97]
PT_second_>13.25seconds_PT_ratio≤99_Creatinin>83.67umol/l_age>54y.o_height>161cm_MTHFR_1298_wt_FV_1299_wt	1	2	33	98	2.7%	1.48 [0.36; 6.10]
PT_second_>13.25seconds_PT_ratio≤99_Creatinin≤83.67umol/l_age>54y.o_height>161cm_MTHFR_1298_wt_FV_1299_wt	1	2	33	98	2.7%	1.48 [0.36; 6.10]
PT_second_>13.25seconds_PT_ratio≤99_Creatinin≤83.67umol/l_age>54y.o_height≤161cm_MTHFR_1298_wt_FV_1299_wt	1	2	33	98	2.7%	1.48 [0.36; 6.10]
PT_second_≤13.25seconds_PT_ratio≤99_Creatinin≤83.67umol/l_age>54y.o_height>161cm_MTHFR_1298_wt_FV_1299_wt	1	2	33	98	2.7%	1.48 [0.36; 6.10]
PT_second_>13.25seconds_PT_ratio≤99_Creatinin≤83.67umol/l_age≤54y.o_height≤161cm_MTHFR_1298_wt_FV_1299_wt	2	4	32	96	5.2%	1.50 [0.54; 4.16]
PT_second_>13.25seconds_PT_ratio≤99_Creatinin>83.67umol/l_age≤54y.o_height>161cm_MTHFR_1298_wt_FV_1299_wt	2	3	32	97	3.9%	2.02 [0.86; 4.72]
PT_second_≤13.25seconds_PT_ratio>99_Creatinin>83.67umol/l_age≤54y.o_height>161cm_MTHFR_1298_hetero_FV_1299_wt	2	3	32	97	3.9%	2.02 [0.86; 4.72]
PT_second_>13.25seconds_PT_ratio>99_Creatinin≤83.67umol/l_age>54y.o_height≤161cm_MTHFR_1298_homo_FV_1299_wt	1	1	33	99	2.7%	2.97 [2.25; 3.91]
PT_second_>13.25seconds_PT_ratio≤99_Creatinin>83.67umol/l_age>54y.o_height>161cm_MTHFR_1298_hetero_FV_1299_hetero	1	1	33	99	2.7%	2.97 [2.25; 3.91]
PT_second_>13.25seconds_PT_ratio≤99_Creatinin≤83.67umol/l_age>54y.o_height≤161cm_MTHFR_1298_homo_FV_1299_wt	1	1	33	99	2.7%	2.97 [2.25; 3.91]
PT_second_>13.25seconds_PT_ratio≤99_Creatinin≤83.67umol/l_age≤54y.o_height>161cm_MTHFR_1298_homo_FV_1299_hetero	1	1	33	99	2.7%	2.97 [2.25; 3.91]
PT_second_>13.25seconds_PT_ratio≤99_Creatinin≤83.67umol/l_age≤54y.o_height>161cm_MTHFR_1298_wt_FV_1299_wt	1	1	33	99	2.7%	2.97 [2.25; 3.91]
PT_second_≤13.25seconds_PT_ratio>99_Creatinin>83.67umol/l_age≤54y.o_height≤161cm_MTHFR_1298_wt_FV_1299_wt	1	1	33	99	2.7%	2.97 [2.25; 3.91]
PT_second_≤13.25seconds_PT_ratio≤99_Creatinin>83.67umol/l_age>54y.o_height≤161cm_MTHFR_1298_hetero_FV_1299_wt	1	1	33	99	2.7%	2.97 [2.25; 3.91]
PT_second_≤13.25seconds_PT_ratio≤99_Creatinin≤83.67umol/l_age>54y.o_height≤161cm_MTHFR_1298_wt_FV_1299_wt	1	1	33	99	2.7%	2.97 [2.25; 3.91]
PT_second_≤13.25seconds_PT_ratio≤99_Creatinin≤83.67umol/l_age≤54y.o_height>161cm_MTHFR_1298_wt_FV_1299_wt	1	1	33	99	2.7%	2.97 [2.25; 3.91]
PT_second_≤13.25seconds_PT_ratio>99_Creatinin>83.67umol/l_age>54y.o_height>161cm_MTHFR_1298_wt_FV_1299_wt	2	2	32	98	3.9%	3.03 [2.29; 4.02]
PT_second_≤13.25seconds_PT_ratio≤99_Creatinin>83.67umol/l_age>54y.o_height>161cm_MTHFR_1298_hetero_FV_1299_wt	2	2	32	98	3.9%	3.03 [2.29; 4.02]
PT_second_>13.25seconds_PT_ratio≤99_Creatinin>83.67umol/l_age>54y.o_height≤161cm_MTHFR_1298_wt_FV_1299_wt	3	3	31	97	5.1%	3.10 [2.32; 4.13]
**Total (95% CI)**		**63**		**2537**	**100.0%**	**1.59 [1.28; 1.98]**
**Prediction interval**						**[2.64; 3.12]**

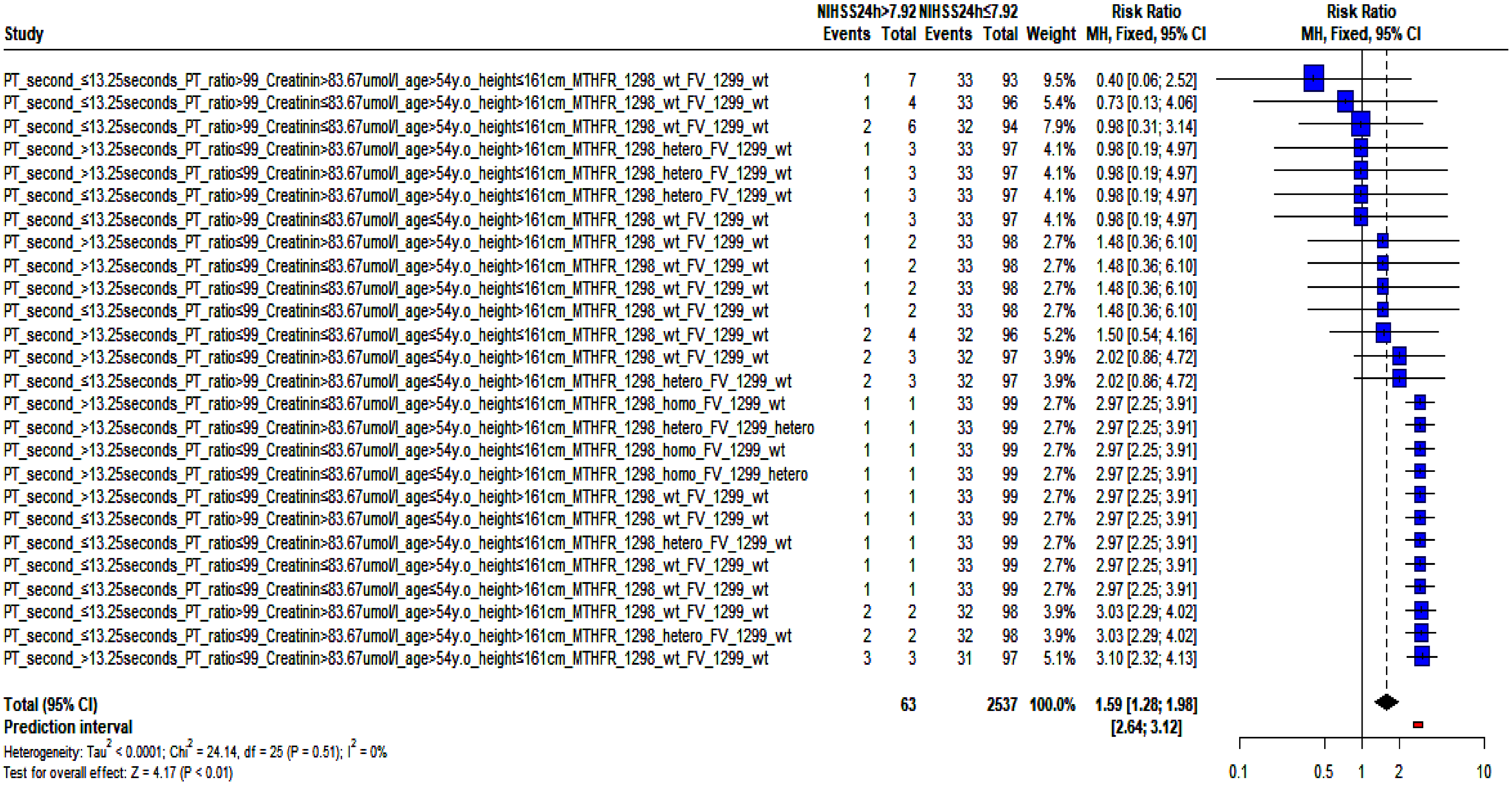

Heterogeneity: Tau^2 < 0.0001; Chi^2 = 24.14, df = 25 (P = 0.51); I^2 = 0%
Test for overall effect: Z = 4.17 (P < 0.01)

Figure S13. Forest plot of the risk of a Glasgow Coma Scale score >12.77 in cluster 11 (MTHFR-A1298C genotype and FV-H1299R genotype) with BMI.

Study	Glasgow>12.77 Events	Glasgow>12.77 Total	Glasgow≤12.77 Events	Glasgow≤12.77 Total	Weight	Risk Ratio MH, Fixed, 95% CI
BMI≤20.58_MTHFR_1298_hetero_FV_1299_wt	5	7	90	93	9.1%	0.74 [0.46; 1.18]
BMI≤20.58_MTHFR_1298_wt_FV_1299_wt	10	12	85	88	14.7%	0.86 [0.67; 1.11]
BMI>20.58_MTHFR_1298_hetero_FV_1299_wt	28	29	67	71	27.9%	1.02 [0.94; 1.12]
BMI>20.58_MTHFR_1298_hetero_FV_1299_hetero	1	1	94	99	2.7%	1.05 [1.01; 1.10]
BMI>20.58_MTHFR_1298_homo_FV_1299_hetero	1	1	94	99	2.7%	1.05 [1.01; 1.10]
BMI>20.58_MTHFR_1298_wt_FV_1299_hetero	2	2	93	98	4.0%	1.05 [1.01; 1.10]
BMI>20.58_MTHFR_1298_homo_FV_1299_wt	4	4	91	96	6.4%	1.05 [1.01; 1.10]
BMI>20.58_MTHFR_1298_wt_FV_1299_wt	44	44	51	56	32.6%	1.10 [1.01; 1.19]
**Total (95% CI)**		**100**		**700**	**100.0%**	**0.97 [0.90; 1.05]**
**Prediction interval**						**[1.02; 1.08]**

Heterogeneity: $Tau^2 < 0.0001$; $Chi^2 = 5.90$, df = 7 (P = 0.55); $I^2 = 0\%$

Test for overall effect: Z = -0.66 (P = 0.51)

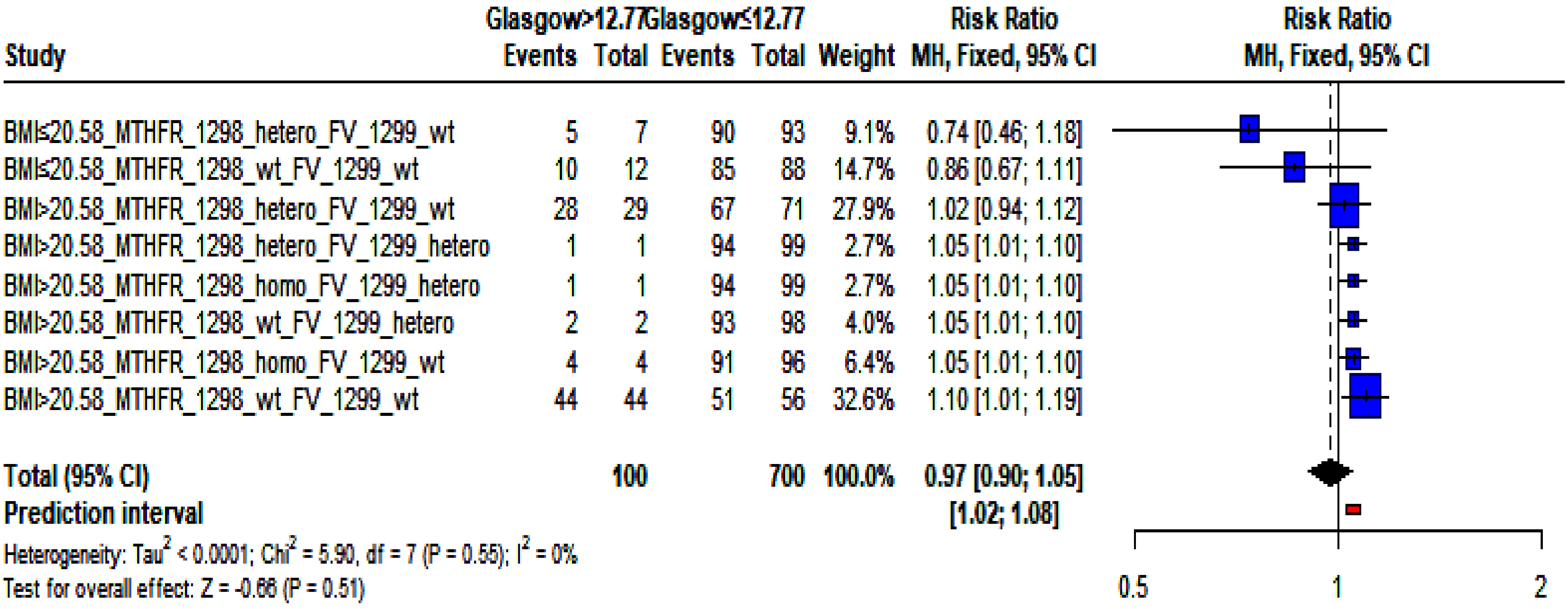

Figure S14. Forest plot of the risk of a modified Rankin scale score >2.86 in cluster 13 (FXIII Val34Leu genotype) with international normalized ratio (INR), prothrombin (PT) time and ratio, creatinine, and number of infarcts on computed tomography (CT).

Study	Rankin>2.86 Events	Rankin>2.86 Total	Rankin≤2.86 Events	Rankin≤2.86 Total	Weight	Risk Ratio MH, Fixed, 95% CI
INR>1.02_PT_second_>13.25seconds_PT_ratio≤99_Creatinin≤83.67umol/l_N_infarctsCT≤2_FXIII_Val34Leu_wt	2	12	24	88	31.2%	0.61 [0.16; 2.27]
INR≤1.02_PT_second_>13.25seconds_PT_ratio>99_Creatinin>83.67umol/l_N_infarctsCT≤2_FXIII_Val34Leu_wt	0	2	26	98	8.5%	0.74 [0.06; 9.06]
INR≤1.02_PT_second_≤13.25seconds_PT_ratio>99_Creatinin>83.67umol/l_N_infarctsCT>2_FXIII_Val34Leu_wt	2	7	24	93	18.2%	1.11 [0.33; 3.75]
INR>1.02_PT_second_>13.25seconds_PT_ratio≤99_Creatinin>83.67umol/l_N_infarctsCT≤2_FXIII_Val34Leu_homo	0	1	26	99	5.6%	1.25 [0.13; 12.32]
INR>1.02_PT_second_≤13.25seconds_PT_ratio≤99_Creatinin≤83.67umol/l_N_infarctsCT>2_FXIII_Val34Leu_wt	0	1	26	99	5.6%	1.25 [0.13; 12.32]
INR>1.02_PT_second_≤13.25seconds_PT_ratio≤99_Creatinin≤83.67umol/l_N_infarctsCT≤2_FXIII_Val34Leu_hetero	0	1	26	99	5.6%	1.25 [0.13; 12.32]
INR>1.02_PT_second_>13.25seconds_PT_ratio≤99_Creatinin≤83.67umol/l_N_infarctsCT>2_FXIII_Val34Leu_wt	1	2	25	98	5.4%	1.96 [0.47; 8.16]
INR>1.02_PT_second_>13.25seconds_PT_ratio≤99_Creatinin>83.67umol/l_N_infarctsCT>2_FXIII_Val34Leu_wt	4	5	22	95	11.9%	3.45 [1.95; 6.12]
INR≤1.02_PT_second_>13.25seconds_PT_ratio≤99_Creatinin>83.67umol/l_N_infarctsCT>2_FXIII_Val34Leu_wt	2	2	24	98	7.8%	4.02 [2.85; 5.67]
**Total (95% CI)**		**33**		**867**	**100.0%**	**1.39 [0.89; 2.16]**
**Prediction interval**						**[0.43; 8.74]**

Heterogeneity: $Tau^2 = 0.3214$; $Chi^2 = 14.44$, df = 8 (P = 0.07); $I^2 = 45\%$
Test for overall effect: Z = 1.44 (P = 0.15)

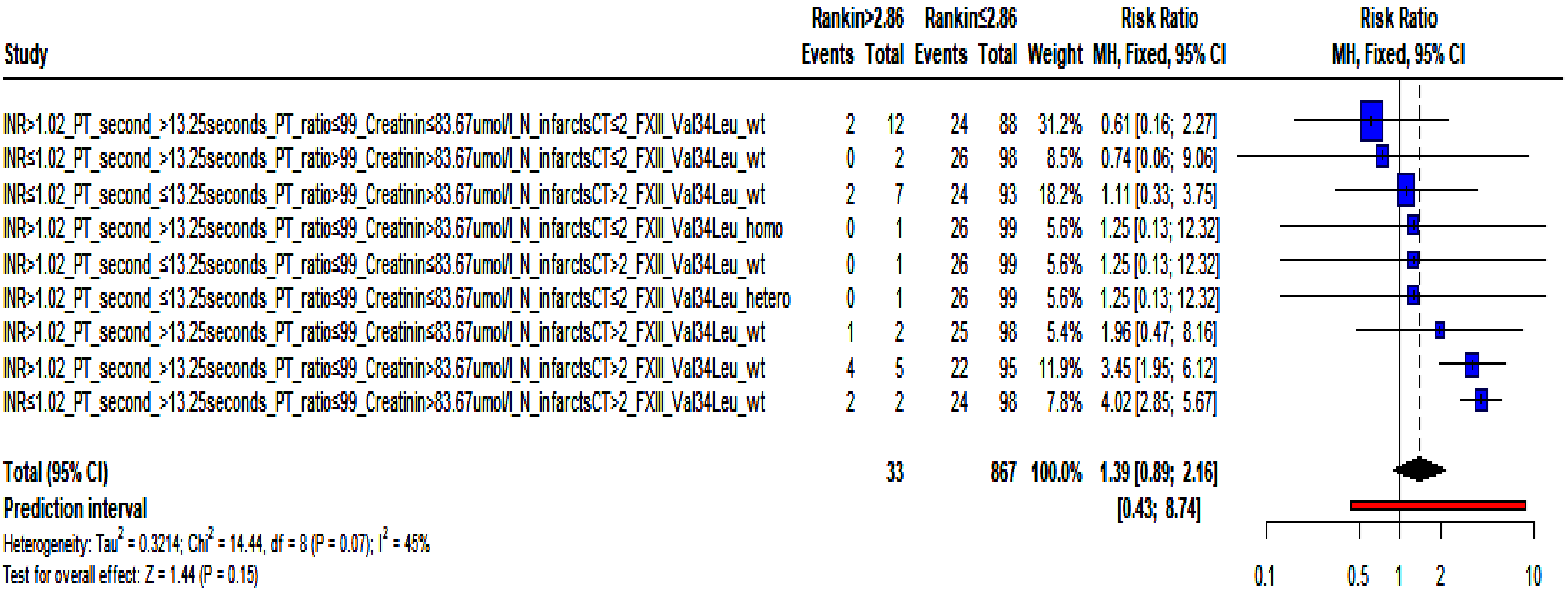

Figure S15. Forest plot of the risk of a National Institutes of Health Stroke Scale (NIHSS) score at admission >9.83 in cluster 13 (FXIII Val34Leu genotype) with prothrombin (PT) time and ratio, creatinine, patient age, and patient height.

Study	NIHSS0h>9.83 Events	NIHSS0h>9.83 Total	NIHSS0h≤9.83 Events	NIHSS0h≤9.83 Total	Weight	Risk Ratio MH, Fixed, 95% CI
PT_second_≤13.25seconds_PT_ratio>99_Creatinin≤83.67umol/l_age>54y.o_height≤161cm_FXIII_Val34Leu_wt	2	12	25	88	16.5%	0.59 [0.16; 2.17]
PT_second_≤13.25seconds_PT_ratio>99_Creatinin>83.67umol/l_age>54y.o_height≤161cm_FXIII_Val34Leu_wt	2	10	25	90	13.7%	0.72 [0.20; 2.60]
PT_second_>13.25seconds_PT_ratio≤99_Creatinin≤83.67umol/l_age>54y.o_height≤161cm_FXIII_Val34Leu_wt	1	4	26	96	5.7%	0.92 [0.16; 5.20]
PT_second_≤13.25seconds_PT_ratio>99_Creatinin≤83.67umol/l_age>54y.o_height>161cm_FXIII_Val34Leu_wt	1	4	26	96	5.7%	0.92 [0.16; 5.20]
PT_second_>13.25seconds_PT_ratio≤99_Creatinin>83.67umol/l_age≤54y.o_height>161cm_FXIII_Val34Leu_wt	1	3	26	97	4.3%	1.24 [0.24; 6.37]
PT_second_>13.25seconds_PT_ratio≤99_Creatinin≤83.67umol/l_age>54y.o_height>161cm_FXIII_Val34Leu_wt	1	3	26	97	4.3%	1.24 [0.24; 6.37]
PT_second_≤13.25seconds_PT_ratio>99_Creatinin>83.67umol/l_age≤54y.o_height>161cm_FXIII_Val34Leu_wt	2	6	25	94	8.2%	1.25 [0.38; 4.08]
PT_second_>13.25seconds_PT_ratio≤99_Creatinin>83.67umol/l_age>54y.o_height>161cm_FXIII_Val34Leu_wt	3	8	24	92	10.5%	1.44 [0.55; 3.75]
PT_second_≤13.25seconds_PT_ratio≤99_Creatinin≤83.67umol/l_age>54y.o_height>161cm_FXIII_Val34Leu_wt	1	2	26	98	2.9%	1.88 [0.45; 7.83]
PT_second_≤13.25seconds_PT_ratio≤99_Creatinin≤83.67umol/l_age≤54y.o_height>161cm_FXIII_Val34Leu_wt	1	2	26	98	2.9%	1.88 [0.45; 7.83]
PT_second_≤13.25seconds_PT_ratio≤99_Creatinin>83.67umol/l_age>54y.o_height>161cm_FXIII_Val34Leu_wt	3	5	24	95	6.6%	2.38 [1.07; 5.26]
PT_second_>13.25seconds_PT_ratio≤99_Creatinin≤83.67umol/l_age≤54y.o_height>161cm_FXIII_Val34Leu_wt	2	3	25	97	4.1%	2.59 [1.09; 6.16]
PT_second_≤13.25seconds_PT_ratio>99_Creatinin>83.67umol/l_age>54y.o_height>161cm_FXIII_Val34Leu_wt	2	3	25	97	4.1%	2.59 [1.09; 6.16]
PT_second_>13.25seconds_PT_ratio≤99_Creatinin>83.67umol/l_age>54y.o_height≤161cm_FXIII_Val34Leu_wt	4	6	23	94	7.6%	2.72 [1.40; 5.31]
PT_second_≤13.25seconds_PT_ratio≤99_Creatinin>83.67umol/l_age≤54y.o_height>161cm_FXIII_Val34Leu_wt	1	1	26	99	2.9%	3.75 [2.71; 5.20]
**Total (95% CI)**		**72**		**1428**	**100.0%**	**1.43 [1.05; 1.94]**
**Prediction interval**						**[0.81; 4.54]**

Heterogeneity: $Tau^2 = 0.1309$; $Chi^2 = 21.25$, df = 14 (P = 0.10); $I^2 = 34\%$
Test for overall effect: Z = 2.27 (P = 0.02)

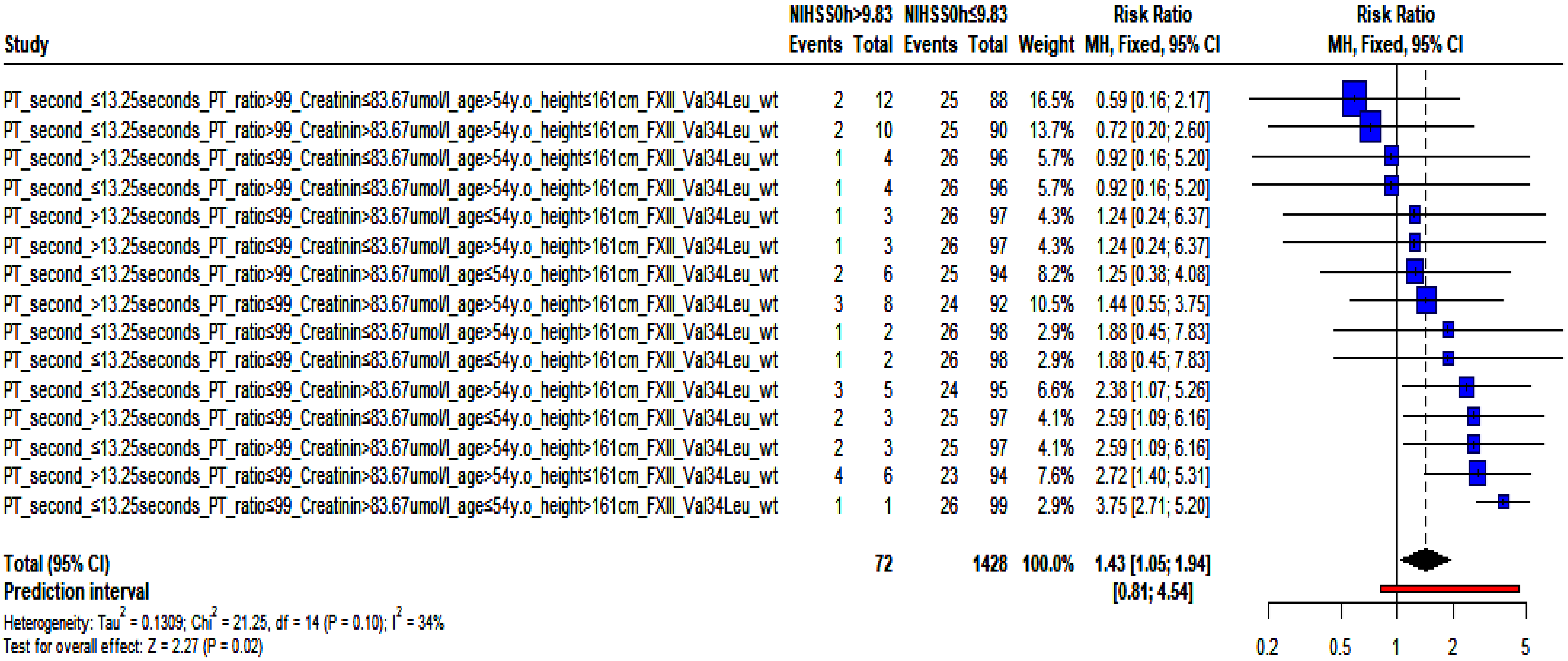

Figure S16. Forest plot of the risk of a National Institutes of Health Stroke Scale (NIHSS) score after 24 hours >7.92 in cluster 13 (FXIII Val34Leu genotype) with prothrombin (PT) time and ratio, creatinine, patient age, and patient height.

Study	NIHSS24h>7.92 Events	NIHSS24h>7.92 Total	NIHSS24h≤7.92 Events	NIHSS24h≤7.92 Total	Weight	Risk Ratio MH, Fixed, 95% CI
PT_second_≤13.25seconds_PT_ratio>99_Creatinin≤83.67umol/l_age>54y.o_height≤161cm_FXIII_Val34Leu_wt	2	12	32	88	13.3%	0.46 [0.13; 1.67]
PT_second_≤13.25seconds_PT_ratio>99_Creatinin≤83.67umol/l_age≤54y.o_height>161cm_FXIII_Val34Leu_wt	1	6	33	94	6.9%	0.47 [0.08; 2.90]
PT_second_≤13.25seconds_PT_ratio>99_Creatinin>83.67umol/l_age>54y.o_height≤161cm_FXIII_Val34Leu_wt	2	10	32	90	11.1%	0.56 [0.16; 2.00]
PT_second_≤13.25seconds_PT_ratio>99_Creatinin≤83.67umol/l_age>54y.o_height>161cm_FXIII_Val34Leu_wt	1	4	33	96	4.6%	0.73 [0.13; 4.06]
PT_second_≤13.25seconds_PT_ratio>99_Creatinin>83.67umol/l_age≤54y.o_height>161cm_FXIII_Val34Leu_wt	2	6	32	94	6.7%	0.98 [0.31; 3.14]
PT_second_>13.25seconds_PT_ratio≤99_Creatinin≤83.67umol/l_age>54y.o_height>161cm_FXIII_Val34Leu_wt	1	3	33	97	3.4%	0.98 [0.19; 4.97]
PT_second_>13.25seconds_PT_ratio≤99_Creatinin>83.67umol/l_age>54y.o_height>161cm_FXIII_Val34Leu_wt	3	8	31	92	8.6%	1.11 [0.43; 2.85]
PT_second_>13.25seconds_PT_ratio≤99_Creatinin≤83.67umol/l_age≤54y.o_height≤161cm_FXIII_Val34Leu_wt	2	5	32	95	5.5%	1.19 [0.39; 3.60]
PT_second_≤13.25seconds_PT_ratio≤99_Creatinin>83.67umol/l_age>54y.o_height>161cm_FXIII_Val34Leu_wt	2	5	32	95	5.5%	1.19 [0.39; 3.60]
PT_second_≤13.25seconds_PT_ratio>99_Creatinin>83.67umol/l_age≤54y.o_height≤161cm_FXIII_Val34Leu_wt	1	2	33	98	2.3%	1.48 [0.36; 6.10]
PT_second_≤13.25seconds_PT_ratio≤99_Creatinin>83.67umol/l_age>54y.o_height≤161cm_FXIII_Val34Leu_wt	1	2	33	98	2.3%	1.48 [0.36; 6.10]
PT_second_≤13.25seconds_PT_ratio≤99_Creatinin≤83.67umol/l_age>54y.o_height>161cm_FXIII_Val34Leu_wt	1	2	33	98	2.3%	1.48 [0.36; 6.10]
PT_second_≤13.25seconds_PT_ratio≤99_Creatinin≤83.67umol/l_age≤54y.o_height>161cm_FXIII_Val34Leu_wt	1	2	33	98	2.3%	1.48 [0.36; 6.10]
PT_second_>13.25seconds_PT_ratio≤99_Creatinin≤83.67umol/l_age>54y.o_height≤161cm_FXIII_Val34Leu_wt	2	4	32	96	4.4%	1.50 [0.54; 4.16]
PT_second_>13.25seconds_PT_ratio≤99_Creatinin>83.67umol/l_age≤54y.o_height>161cm_FXIII_Val34Leu_wt	2	3	32	97	3.3%	2.02 [0.86; 4.72]
PT_second_>13.25seconds_PT_ratio≤99_Creatinin≤83.67umol/l_age≤54y.o_height>161cm_FXIII_Val34Leu_wt	2	3	32	97	3.3%	2.02 [0.86; 4.72]
PT_second_≤13.25seconds_PT_ratio>99_Creatinin>83.67umol/l_age>54y.o_height>161cm_FXIII_Val34Leu_wt	2	3	32	97	3.3%	2.02 [0.86; 4.72]
PT_second_>13.25seconds_PT_ratio≤99_Creatinin>83.67umol/l_age>54y.o_height≤161cm_FXIII_Val34Leu_wt	4	6	30	94	6.2%	2.09 [1.10; 3.95]
PT_second_>13.25seconds_PT_ratio>99_Creatinin≤83.67umol/l_age>54y.o_height≤161cm_FXIII_Val34Leu_wt	1	1	33	99	2.3%	2.97 [2.25; 3.91]
PT_second_≤13.25seconds_PT_ratio≤99_Creatinin≤83.67umol/l_age>54y.o_height≤161cm_FXIII_Val34Leu_wt	1	1	33	99	2.3%	2.97 [2.25; 3.91]
**Total (95% CI)**		**88**		**1912**	**100.0%**	**1.15 [0.88; 1.50]**
**Prediction interval**						**[0.71; 3.70]**

Heterogeneity: $Tau^2 = 0.1354$; $Chi^2 = 32.49$, df = 19 (P = 0.03); $I^2 = 42\%$
Test for overall effect: Z = 1.05 (P = 0.30)

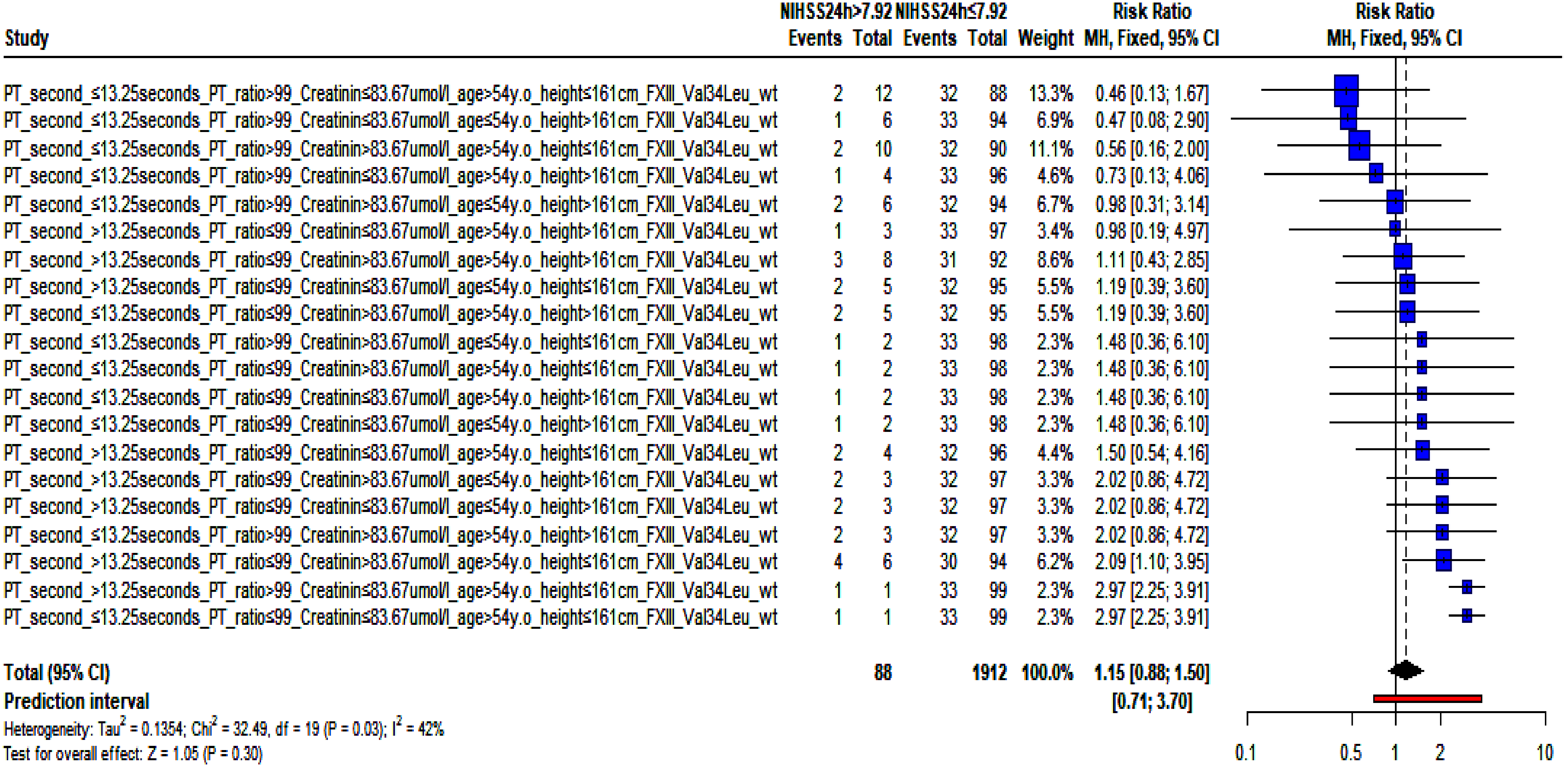

Figure S17. Forest plot of the risk of a Glasgow Coma Scale score >12.77 in cluster 13 (FXIII Val34Leu genotype) with BMI.

Study	Glasgow>12.77 Events	Glasgow>12.77 Total	Glasgow≤12.77 Events	Glasgow≤12.77 Total	Weight	Risk Ratio MH, Fixed, 95% CI
BMI≤20.58_FXIII_Val34Leu_wt	14	18	81	82	46.9%	0.79 [0.61; 1.01]
BMI>20.58_FXIII_Val34Leu_homo	1	1	94	99	6.0%	1.05 [1.01; 1.10]
BMI≤20.58_FXIII_Val34Leu_hetero	1	1	94	99	6.0%	1.05 [1.01; 1.10]
BMI>20.58_FXIII_Val34Leu_wt	79	80	16	20	41.2%	1.23 [0.99; 1.54]
**Total (95% CI)**		**100**		**300**	**100.0%**	**0.97 [0.83; 1.14]**
**Prediction interval**						**[0.98; 1.13]**

Heterogeneity: $Tau^2 < 0.0001$; $Chi^2 = 7.26$, df = 3 (P = 0.06); $I^2 = 59\%$
Test for overall effect: Z = -0.35 (P = 0.73)

Risk Ratio
MH, Fixed, 95% CI

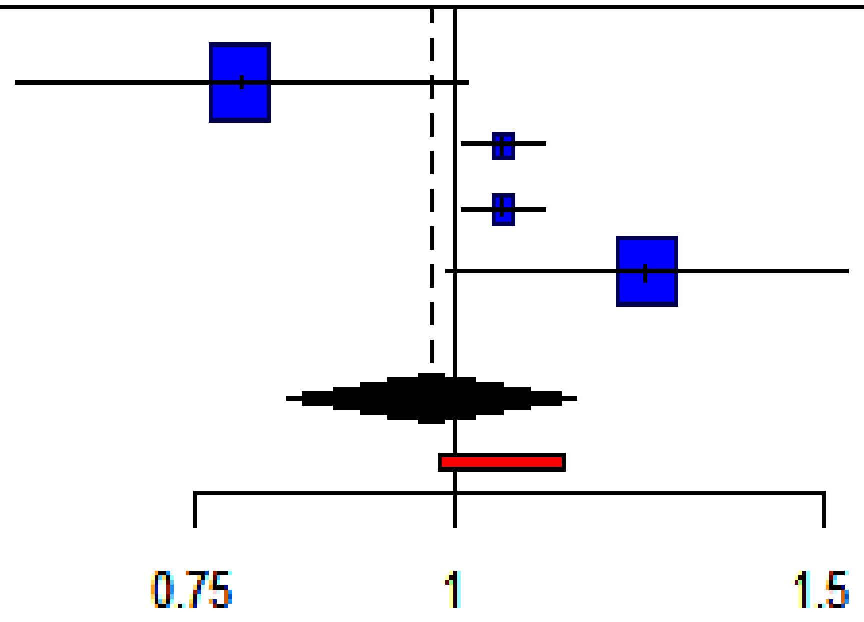

Supplement: Multimedia Appendix 2 [file bioinform_v5i1e56884_app2.pdf]
